# Targeting ABL kinases overcomes melanoma immune evasion following resistance to MAPK inhibition

**DOI:** 10.1038/s41467-026-76864-y

**Published:** 2026-08-21

**Authors:** Rakshamani Tripathi, Bhuvanesh Sukhlal Kalal, Shirin Soni, Chezney Boothe, Anastasia Lyon, Christina Meeks, Daheng He, Phil Fang Cheng, Mitchell P. Levesque, Jinpeng Liu, Chi Wang, Rina Plattner

**Affiliations:** 1 Department of Pharmacology and Nutritional Sciences, University of Kentucky College of Medicine, Lexington, KY USA; 2 Biostatistics and Bioinformatics Shared Resource Facility; Markey Cancer Center, University of Kentucky College of Medicine, Lexington, KY USA; 3 Department of Dermatology, University of Zurich Hospital and Faculty of Medicine, University of Zurich, Zurich, Switzerland

**Keywords:** Melanoma, Cancer microenvironment, Chemokines, Targeted therapies

## Abstract

MAPK inhibitors (MAPKi) are important treatment options for some patients with metastatic melanoma; however, resistance inevitably develops in the vast majority of cases (>70%). Here, using immune-proficient mouse models, single-cell RNA sequencing, flow cytometry, and datasets from patients treated with MAPKi, we demonstrate that ABL kinases (ABL1/2) promote MAPKi resistance not only by impacting intracellular signaling but also by driving secretion of chemokines that suppress the immune microenvironment. Targeting ABL1/2 reduces chemokine secretion by melanoma cells, prevents immunosuppressive myeloid-derived suppressor cell (MDSC) infiltration, and promotes accumulation of cytotoxic CD8^+^ T cells. Depletion of MDSCs prevents resistance, and depletion of CD8^+^ T cells or re-expression of chemokines blocks ABL inhibitors from preventing resistance. Importantly, CXCR2 receptor ligand expression and ABL1/2 activity are reduced in patients who respond to treatment but increased during resistance. Thus, the dual role of ABL1/2 in proliferation/survival and immune suppression makes them attractive targets for potential treatments.

## Abstract (web summary)

Although MAP kinase inhibitors (MAPKi) can initially reduce tumour burden in metastatic melanoma, resistance to treatment often occurs. Here, the authors demonstrate that concomitant treatment with ABL kinase inhibitors overcomes tumour resistance to MAPKi by promoting the accumulation of cytotoxic T lymphocytes and inhibiting the infiltration of immunosuppressive cells.

## Introduction

Melanoma is a deadly disease, particularly once it has metastasized to distant organ sites (35% 5-year survival; ACS, 2025). While immune-checkpoint inhibitors (ICI) have revolutionized treatment, only some patients respond, others cannot tolerate the adverse effects, and still others develop resistance^1^. Activation of the MAPK pathway plays a critical role in melanoma development and progression^2^. Inhibitors targeting BRAF and MEK (BRAFi, MEKi) are effective therapies for patients with *BRAF*-mutant melanomas, but are not usually curative^3^. MAPKi are not used for melanomas with *NRAS* mutations due to lack of benefit (BRAFi*)* or modest benefit due to intrinsic and acquired resistance (MEKi)^4^. Previously, we showed that ABL family non-receptor tyrosine kinases (ABL1, ABL2; ABL1/2), which promote the development of human leukemia, are activated during MAPKi resistance and drive ERK reactivation, proliferation, and survival in *BRAF*-mutant melanoma^5^, and cooperate with DDR1 (discoidin receptor 1) to induce intrinsic and acquired MEKi resistance in *NRAS*-mutant melanoma^6^. Targeting ABL1/2 (and DDR1) with the second-generation anti-leukemia drug, nilotinib, reverses resistance and prevents its onset in xenograft models^5,6^.

The immune system plays a major role in the response to ICI and targeted therapy^7^. Antigen stimulation of naive CD8^+^ cells causes activation followed by expansion and differentiation into effector cells and memory cells. Most effector cells then die, and the memory cells provide long-term protection against the antigen^8^. When T cells are continuously exposed to antigen, they become “exhausted” and have dysfunctional proliferation and cytotoxic functions^8^. Antigen stimulation also induces naive CD4^+^ T cells to differentiate into Th1 (T helper) cells, which assist CD8^+^ T cells in cytotoxic responses^9^. In naive patients/tumors, BRAFi/MEKi treatment induces cytotoxic CD8^+^ T cell infiltration that assists in drug efficacy^7^. However, during resistance, cytotoxic T cells are reduced, and the ones that remain convert to an exhausted state, which may occur as soon as 24 h after tumor antigen exposure^7,10^. In contrast to *BRAF*-mutant melanomas, MEKi reduces CD8^+^ T cell infiltration in *NRAS*-mutant melanoma^11^; thus, immune suppression may contribute to intrinsic resistance in this subtype.

Melanoma chemokine secretion induces tumor infiltration of pro-tumorigenic, immunosuppressive cells such as Tregs, myeloid-derived suppressor cells (MDSCs), and tumor-associated macrophages (TAMs) that suppress migration, proliferation, and cytotoxic activity of T cells, natural killer (NK) cells, and B cells^7,12,13^. While T cell function has been implicated in targeted therapy effectiveness, the role of immunosuppressive cells is less clear. MDSCs originate in the bone marrow during cancer, and other pathophysiological conditions^14,15^ and consist of polymorphonuclear (PMN-MDSC) MDSCs, which arise from the granulocytic (neutrophil) lineage, and M-MDSCs, which are from the monocytic lineage^16^. MDSCs arise from immature progenitors or directly from monocytes or neutrophils^15^. M-MDSCs can differentiate into PMN-MDSCs through transcriptional silencing of RB1^15,17^, and differentiate into TAMs in the hypoxic tumor microenvironment^15,16^. PMN-MDSCs require cell-cell contact to inhibit T cell function, whereas M-MDSCs secrete factors that inhibit T cell migration, proliferation, and function^16^. Chemokines secreted by tumor cells, fibroblasts, or immune cells drive MDSC infiltration by binding to receptors on the surface of MDSCs^15,16^. CCL2 binding to CCR2 receptors is a major stimulus for M-MDSC chemotaxis, while CCL5 binding to CCR5 maintains MDSCs in the tumor niche^15^. C-X-C-L chemokines that bind the CXCR2 receptor (e.g., CXCL1, 2, 5, 8) drive granulocyte (PMN-MDSC, neutrophil) chemotaxis^15^.

Here, we show that ABL1/2 induce melanoma cells to secrete chemokines that promote tumor infiltration by immunosuppressive MDSCs, thereby preventing cytotoxic T cell accumulation and function, which is required for MAPKi resistance. Thus, ABL kinases’ dual roles in promoting intracellular proliferation/survival signaling as well as extracellular secretion and immunosuppression provide additional rationale for targeting ABL1/2 in the clinic.

## Results

### ABL1/2 promote cytokine secretion from melanoma cells during MAPKi resistance

**Fig. 1:**
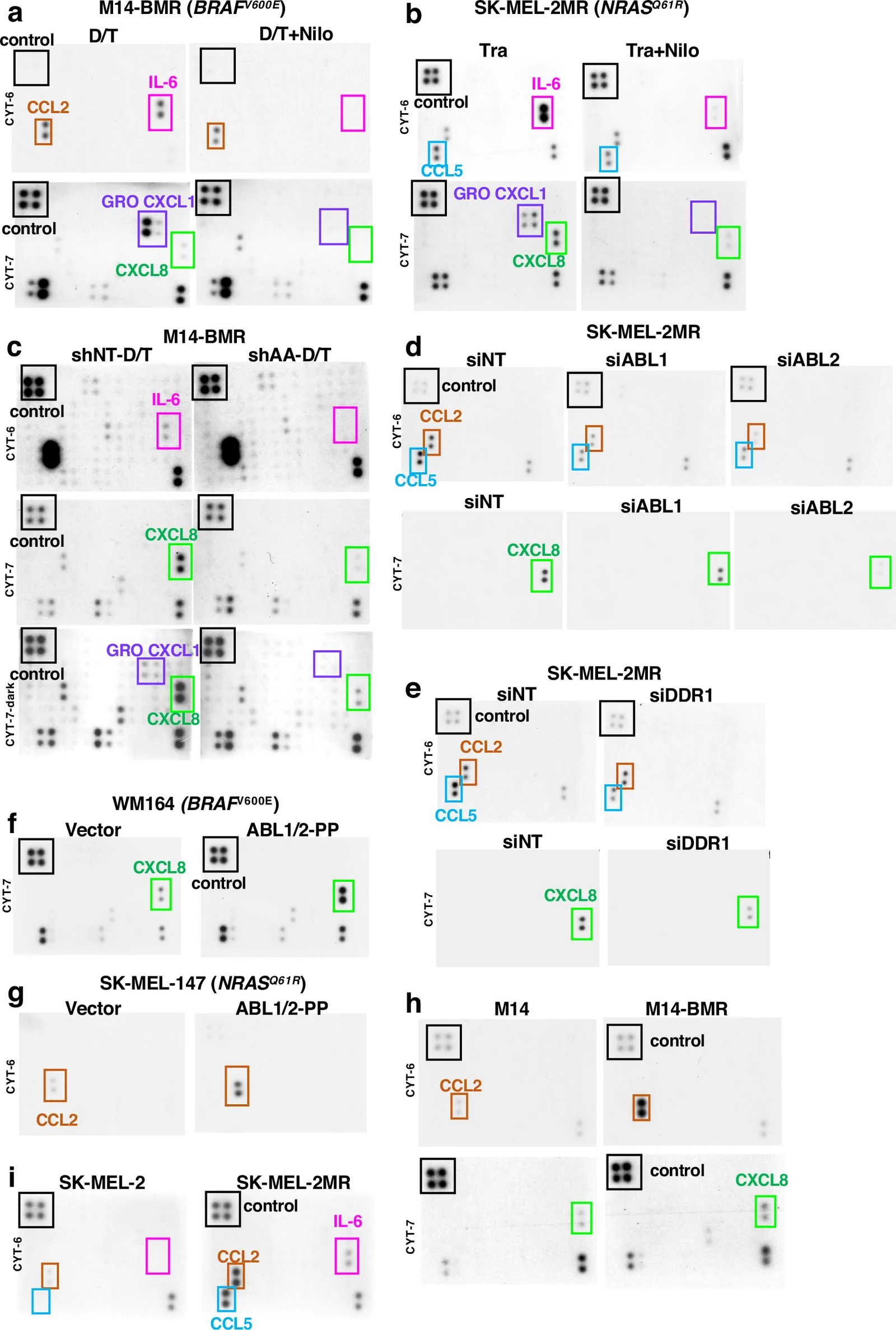
ABL1/2 and DDR1 promote melanoma cytokine secretion. Human cytokine arrays (C1000-CYT-6 and CYT-7 membranes; Raybiotech) were incubated with conditioned media from the serum-starved (0% serum, 12–16 h), drug-treated, or transfected cell lines described below. All drug-treatments occurred during serum-starvation unless otherwise noted. Resistant cells were maintained in dabrafenib/trametinib (D/T, 100 nM/20 nM, *BRAF*-mutant; BMR) or trametinib (Tra, 20 nM, *NRAS*-mutant MR), which is part of their growth media. Serum-starvation/nilotinib treatment times were optimized such that cell viability was >90% when conditioned media was harvested. **a**, **b** M14-BMR (*BRAF*-mutant) or SK-MEL-2MR (*NRAS*-mutant) resistant cells (cultured in D/T or trametinib, respectively) were treated with (vehicle-DMSO) or nilotinib (2.5μM; Nilo) during serum-starvation. **c** M14-BMR cells expressing non-targeting shRNA (shNT) or IPTG-inducible ABL1/2 shRNA were induced with IPTG (1 mM, 6 days), and serum-starved in the presence of D/T. **d**, **e** SK-MEL-2MR cells transfected with siRNAs or non-targeting control (siNT) were maintained in trametinib, and serum-starved 48 h after the transfection in the presence of trametinib. DDR1=discoidin domain receptor 1. *BRAF*-mutant WM164 or *NRAS*-mutant SK-MEL-147 melanoma cells engineered to express constitutively active ABL1 and ABL2 (PP)^5,6^ or vector were treated with D/T **f** or trametinib (**g**, 10 nM) for 8 h to induce resistance signaling followed by serum-starvation in the presence of D/T **f** or trametinib (**g**). **h**, **i** Conditioned media from parental lines was compared to their resistant counterparts: parental M14 versus M14-BMR, and SK-MEL-2 vs SK-MEL-2MR. For all subfigures, cytokine spots and control spots (black boxes) were quantitated with ImageJ (v1.54 g; Supplementary Fig. 1a–d, h, j; see Methods). Analysis of mouse cell lines is in Supplementary Fig. 3. Antibody array maps are shown in the Source Data File. n.s. non-significant.

Previously, we showed that ABL1 and ABL2 drive secretion of pro-invasive molecules (MMPs, cathepsins) that promote melanoma invasion and metastasis^18,19^. Since secretion of cytokines from melanoma cells plays a critical role in shaping the immune microenvironment, which can contribute to therapeutic resistance^7^, we examined whether ABL1/2 promote cytokine secretion in MAPKi-resistant melanoma cells. *BRAF*-mutant cell lines that developed BRAFi/MEKi resistance (dabrafenib/trametinib, D/T; BMR) or *NRAS*-mutant cell lines that acquired MEKi resistance (trametinib; MR) were obtained by culturing parental cells in increasing doses of drugs until resistant to 100 nM/20 nM D/T or 20 nM trametinib, and maintained in D/T or trametinib^5,6^. Resistant cells (in D/T or trametinib) were serum-starved (12–16 h), treated with vehicle or ABL1/2 inhibitor, nilotinib, and conditioned media used to screen human cytokine antibody membrane arrays (120 cytokines). Starvation times were adjusted to maintain high viability (>90% at the time of media collection). The largest effects observed following nilotinib treatment were reduced secretion of GROαβλ (CXCL1,2,3), CXCL1, CXCL8, and IL-6 (Fig. 1a, b and Supplementary Fig. 1a, b). CCL2 and CCL5 were modestly reduced in *BRAF*-mutant lines, whereas the effects were more pronounced in *NRAS*-mutant cells. Silencing ABL1/2 inhibited secretion of many of the same cytokines (Fig. 1c, d and Supplementary Fig. 1c–e, f-left). Under the conditions utilized (12–16 h starvation/nilotinib treatment, 72 h siRNA expression, 6 d IPTG shRNA induction), silencing or inhibiting ABL1/2 had little effect on the cell cycle (Supplementary Fig. 1g), indicating that the observed effects are not due to cell cycle arrest. Expression of constitutively active forms of ABL1/2 (PP)^20^ into parental cells lacking activated ABL1/2^5^ was also sufficient to increase cytokine secretion (Fig. 1f, g and Supplementary Fig. 1h, i). Since ABL1/2 and DDR1 cooperate to drive resistance in *NRAS*-mutant lines^6^, we examined the effect of silencing DDR1. Loss of DDR1 reduced secretion of some of the same cytokines (Fig. 1e and Supplementary Fig. 1d, f-right). Importantly, many of the cytokines regulated by ABL1/2 and DDR1 were increased in resistant cells (compared to parental; Fig. 1h, i and Supplementary Fig. 1j).

### ABL1/2 induce CXCL1, CXCL8 and CCL2 mRNA expression

**Fig. 2:**
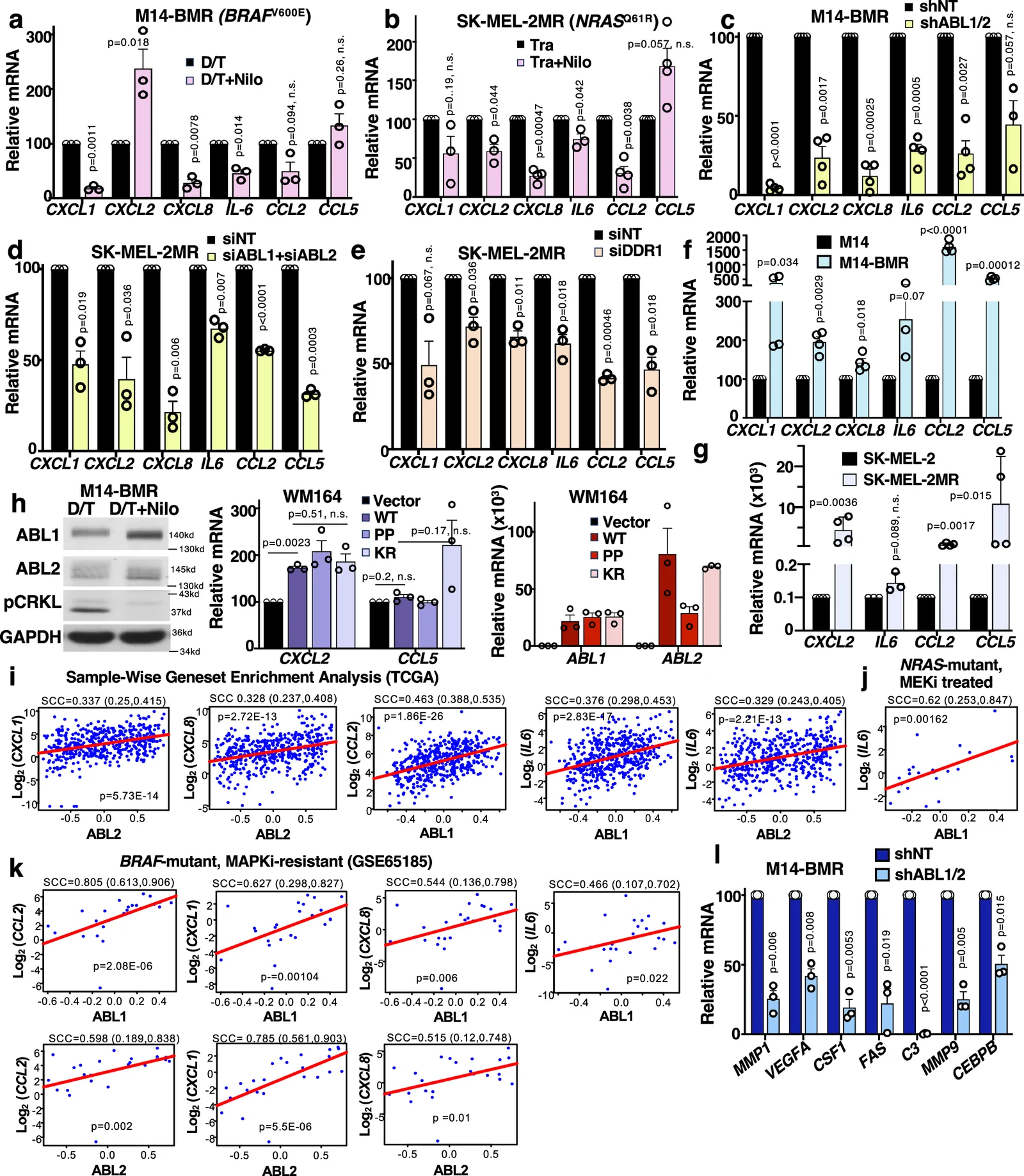
ABL1/2 and DDR1 promote cytokine mRNA expression. **a**–**g** Cytokine mRNA levels were assessed by RT-qPCR using treated/transfected cell lines (described below), and mRNA levels expressed relative to RPS13. For all graphs (**a**–**h**, **l**), mean±SEM is shown from ≥3 biological replicates (open dots), with each biological replicate performed in duplicate. *p*-values are from single-sample *t*-tests (2-tailed) for each individual pair of samples. **a**, **b** *BRAF*-mutant (BMR) or *NRAS*-mutant (MR) cell lines growing in dabrafenib/trametinib (D/T; 100nM/20 nM; *BRAF*-mutant) or trametinib (20 nM; *NRAS*-mutant) were treated with vehicle (DMSO) or nilotinib (2.5 μM; Nilo) for 20–24 h, and the indicated cytokine mRNAs quantitated. **c** BRAFi/MEKi-resistant M14-BMR cells expressing IPTG-inducible ABL1/2 or non-targeting (NT) shRNA were treated with IPTG for 6 d, and RNA used for RT-qPCR. **d**, **e** Resistant cells were transfected with non-targeting (NT), ABL1, ABL2, or DDR1 siRNAs (10 nM). **f**, **g** Comparison of cytokine mRNA expression in parental and resistant cell lines. **h-left** ABL1 and ABL2 expression in nilotinib-treated lysates from resistant cells. Blots are representative of 3 biological replicates (see Source File for other replicates). **h-middle and right** Cytokine mRNA levels were assessed by RT-qPCR in WM164 cells expressing vector, wild-type (WT), kinase inactive (KR), or constitutively active (PP) forms of ABL1 and ABL2, treated with D/T (5 nM/1 nM; 8 h) to induce resistance signaling. *p*-values are from two-tailed single-sample (comparison to vector) and two-sample *t*-tests (WT vs KR). Kinase activities were inferred using sample-wise gene set enrichment analysis in RNAseq from melanoma patient samples (*n* = 471, TCGA), **i**; *NRAS-*mutant, MEKi-treated melanoma patient samples from Nagler et al. (*n* = 23), **j;** or MAPKi-resistant patient samples harboring *BRAF*-mutant melanomas (*n* = 24), **k**. Kinase enrichment scores (“activities”) were determined using downstream target genesets sourced from the IPA Ingenuity Knowledge Base (Supplementary Dataset 1)^26^. Kinase enrichment scores were compared with cytokine mRNAs using Spearman’s correlation coefficient. Correlation coefficients (SCC), 95% confidence limits, and 2-tailed *p*-values are shown. Additional analyses are in Supplementary Fig. 1l. **l** Validation of genes in the IPA ABL1/2 activation genesets (Supplementary Dataset 1). Gene expression was assessed by RT-qPCR in cells expressing non-targeting (NT) or IPTG-inducible ABL1/2 shRNA following 6 d induction with IPTG. Tra trametinib, Nilo nilotinib, D/T dabrafenib/trametinib (BRAFi/MEKi). BMR BRAFi/MEKi-resistant, MR MEKi resistant.

In addition to promoting cytokine secretion, silencing or inhibiting ABL1/2 and/or DDR1 reduced *CXCL1, CXCL8, CCL2, IL-6* mRNAs (Fig. 2a–e and Supplementary Fig. 1e, f), Similar to serum-starved conditions, silencing ABL1/2 in serum conditions at the timepoints utilized (72 h after siRNA transfection, 6 d IPTG shRNA induction) had little effect on the cell cycle (Supplementary Fig. 1k). While IL-6 expression was reduced by nilotinib and si/shRNAs, the level of downregulation (20–50%) did not approximate the massive effects on secretion (99%), indicating that ABL1/2 likely also regulate IL-6 at the protein/secretion level. Many cytokine mRNAs regulated by ABL1/2 were also upregulated in resistant cells (compared to parental; Fig. 2f, g).

Interestingly, while *CCL5* and *CXCL2* mRNAs were reduced by sh/siRNAs, nilotinib increased *CCL5* and, in some cases (M14-BMR) *CXCL2* mRNAs (Fig. 2a–e). ABL1 signals in a kinase-independent manner in some cell contexts^19,21–24^. Interestingly, nilotinib increases ABL1 and ABL2 proteins, and due to the presence of nilotinib, ABL1/2 are kinase-inactive, as evidenced by loss of CRKL phosphorylation (ABL1/2 substrate; Fig. 2h, left panel). Expression of kinase-inactive ABL1 and ABL2 (KR) into cells lacking activated ABL1/2 increased *CXCL2* mRNA, similar to wild-type or constitutively active forms (Fig. 2h, middle and right panels.), indicating that ABL1/2 drive *CXCL2* mRNA expression in a kinase-independent manner. In contrast, expression of kinase-active or kinase-inactive ABL1/2 was not sufficient to increase *CCL5* mRNA (Fig. 2h), indicating that ABL1/2-independent pathways also likely contribute to *CCL5* mRNA expression.

### ABL1/2 activities correlate with cytokine expression in patient samples

ABL1/2 and DDR1 are tightly regulated kinases, and thus, their mRNA levels do not always reflect their activities. Moreover, antibodies recognizing the activated forms cross-react with receptor tyrosine kinases (RTKs) and, therefore, cannot be used for immunohistochemistry. Thus, to determine whether ABL1/2 and DDR1 activities correlate with cytokine expression in patient samples, we utilized an innovative bioinformatics approach. Sample-wise gene set enrichment analyses methods, such as those implemented in the Gene Set Variation Analysis (GSVA) package, transform gene expression data into pathway-centric scores at the single-sample level, where an enrichment score is computed to quantify pathway activities in each sample^25–27^. GSVA provides a powerful way to characterize the activation of kinases by assessing expression of their downstream targets using genesets from the Ingenuity Pathway Database (IPA; see Dataset 1)^25–27^. Based on the enrichment scores obtained from GSVA, we found significant correlations (using Spearman’s correlation coefficient; SCC) between ABL1, ABL2, and DDR1 activities and cytokine mRNA levels in: a) RNAseq data (TCGA) from 471 patients with melanoma (contain *BRAF*- and *NRAS*-mutant samples; Fig. 2i and Supplementary Fig. 1l)^26^; b) an *NRAS*-mutant melanoma dataset obtained from patients treated with MEKi (Fig. 2j, *n* = 23)^28^; and in *BRAF*-mutant melanomas from patients who developed resistance to MAPK inhibitors (Fig. 2k, *n* = 24)^29^. Importantly, genes in the ABL1 and ABL2 activation gene sets are downregulated in cells expressing an shRNA targeting ABL1 and ABL2, thereby validating the gene set (Fig. 2l). Thus, these data are consistent with our cell line data indicating a dependence of cytokine expression on ABL1/2 and/or DDR1 activities.

### Nilotinib prevents and reverses MAPKi resistance and cytokine secretion in mouse melanoma cells from genetically-engineered mouse models

Many of the cytokines whose secretion is regulated by ABL1/2 are known to promote infiltration of pro-tumorigenic immune cells. Thus, ABL1/2 may drive resistance, in part, by promoting immunosuppressive cytokine secretion. To test this hypothesis, first, we established parental/resistant mouse melanoma cell line pairs. Parental melanoma lines were derived previously from genetically engineered mouse models^30–32^. YUMM5.2 (*Braf*^*V600E/WT*^*/Trp53*^*−/−*^), YUMM1.7 (*Braf*^*V600E/WT*^*/Pten*^*−/−*^*/Cdk2n*^*−/−*^) and YUMM3.3 (*Braf*^*V600E/WT*^*/Cdk2n*^*−/−*^) cell lines were derived from *Braf*-mutant genetically engineered mouse models^30^, whereas 1007, 1014 (MaNras1^Q61K^-1007, MaNras2^Q61K^-1014), OSUMMER.12, and OSUMMER.13 [Tyr::CreER(T2); *LSL-Nras61R/R* (*TN61R/R*)] were established from *Nras*-mutant models^31,32^. BRAFi/MEKi (-BMR) resistant lines were established by growing parental cells in increasing concentrations of BRAFi/MEKi (dabrafenib/trametinib; D/T). *Nras*-mutant cell lines that developed resistance to MEKi also were established (-MR). Resistant lines were maintained in drugs. ABL1 and ABL2 are activated in BRAFi/MEKi-resistant human lines and are necessary and sufficient to drive resistance^5^, whereas ABL1/2 and DDR1 are activated in *NRAS*-mutant lines and cooperate to drive MEKi resistance^6^. ABL1/2 activities are also increased in mouse resistant cell lines, as assessed by phosphorylation of ABL1/2 substrate, CRKL, a reliable readout of ABL1 + ABL2 activities^18,33^, and DDR1 activity (pDDR1-pY792) is increased in some *Nras*-mutant resistant lines (Supplementary Fig. 2a)^6^. Similar to human cells^5,6^, MAPKi resistance was reversed by addition of nilotinib to MAPKi in the mouse lines (Supplementary Fig. 2b-d), and ABL-specific (GNF-5) and DDR1-specific (DDR-IN-1; DDR1i) inhibitors cooperate to reverse resistance in *Nras*-mutant lines (Supplementary Fig. 2e). Moreover, in resistant cells, ERK signaling (e.g. pERK, pFOSL1) and in some cases AKT are reactivated in the presence of BRAFi and/or MEKi, and addition of nilotinib blocks reactivation of these pathways (Supplementary Fig. 2f), similar to our findings in human cells^5,6^. To examine the contribution of ABL1/2 to cytokine secretion in the mouse lines, we screened mouse cytokine arrays. CXCL8 is not expressed in the mouse^34^, and thus, could not be examined. Moreover, we did not observe appreciable IL-6 expression in the mouse lines; however, nilotinib inhibited CXCL1, CXCL5, CCL2, and CCL5 secretion (Supplementary Fig. 3a–g). Since CXCL1 and CXCL5 (and CXCL8) bind to the CXCR2 receptor on the surface of immune cells, the effects on the immune system would be predicted to be similar in mouse and human cells. Similar to the human cell lines, mouse *Cxcl1* and *Ccl2* (but not *Ccl5)* mRNAs were reduced by nilotinib treatment (Supplementary Fig. 3h, i).

### Targeting ABL1/2 reverses and prevents MAPKi resistance in immune-competent mouse models

**Fig. 3:**
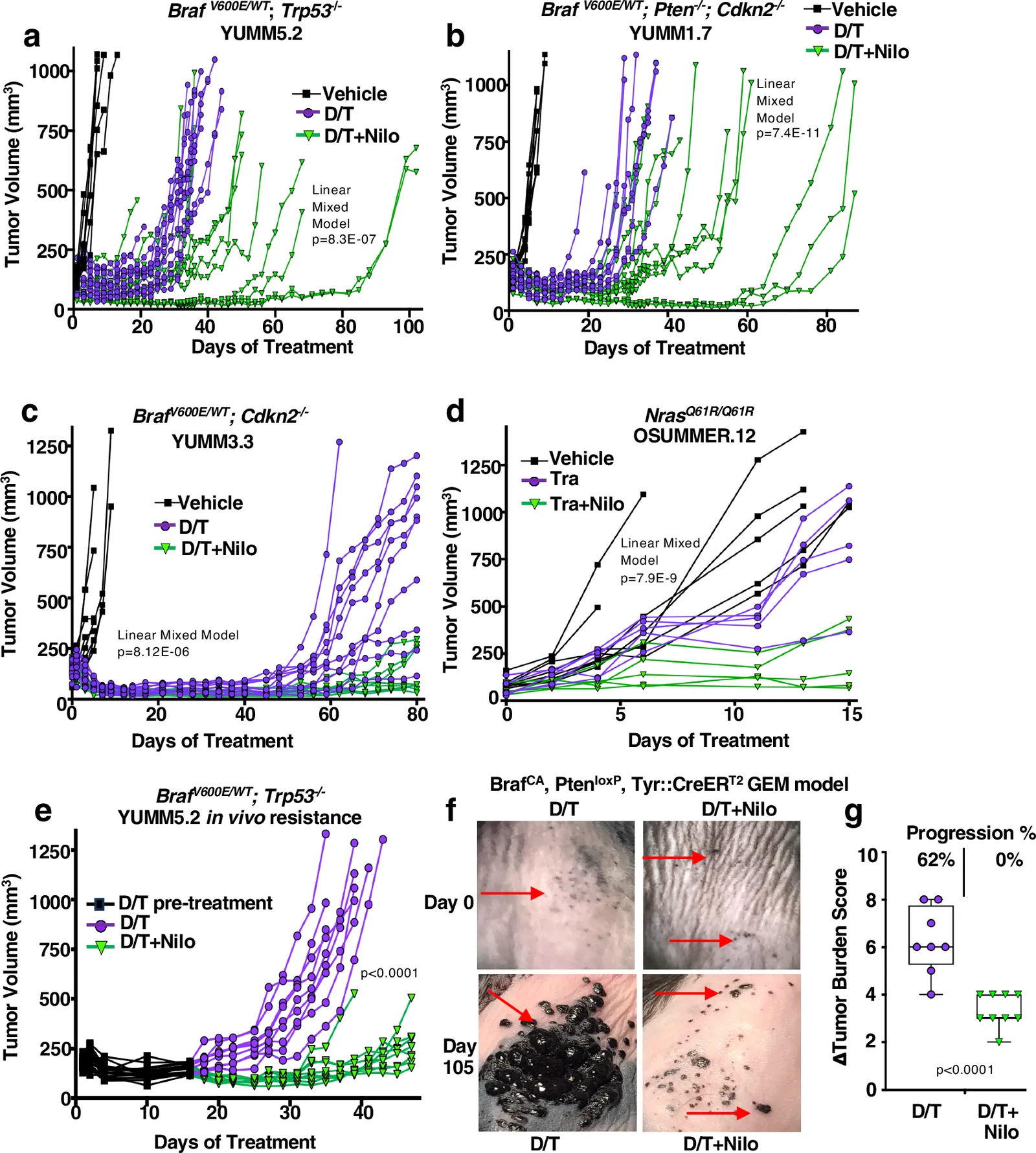
Targeting ABL1/2 prevents and reverses MAPKi resistance in immune-competent mouse models. **a**–**c** C57BL/6 J mice (a,b=male, c=female; 6–7 weeks of age) harboring syngeneic *Braf*-mutant melanomas (75–200 mm^3^)^30^ were randomized to vehicle, BRAFi/MEKi (D/T-dabrafenib-25mg/kg/d, trametinib-0.15 mg/kg/d) or D/T+nilotinib (84 mg/kg/d; Nilo). **d** Female (6–7 weeks old) mice harboring syngeneic *Nras*-mutant melanomas (OSUMMER.12)^32^ were treated with vehicle, trametinib (0.85 mg/kg/d), or trametinib+nilotinib (84 mg/kg/d) when tumors were evident. The number of animals used is as follows. **a, c:** Vehicle (Veh), *n* = 8/group; D/T and D/T+Nilo, *n* = 12/group. **b:** Vehicle, *n* = 8; D/T, *n* = 10, D/T+Nilo, *n* = 12, **d:** *n* = 5/group. Individual mouse tumor growth curves are shown. Mice were euthanized when tumors reached ≈1000–1500 mm^3^ or if they developed an ulceration. *p*-values are from two-tailed linear-mixed model analyses comparing D/T or trametinib to D/T+nilotinib or trametinib+nilotinib. **e** C57BL/6 J male mice (6–7 weeks old) harboring YUMM5.2 parental tumors (*n* = 18) were treated with D/T until tumors regressed and then regrew (black line) and developed resistance, at which time mice were randomized to continue D/T or begin D/T+nilotinib treatment (*n* = 9/group). Mean±SEM tumor volumes are shown; *p*-value=two-sided, two-sample *t*-test. **f**, **g** Male (9) and female (15) *Braf*/*Pten* mice (*Braf* [CA], *Pten* [loxP], Tyr::CreER [T2]; 7–8 weeks old) were treated with 4-hydroxytamoxifen (4HT) topically on their back for 4 days to induce activation of *Braf* (V600E) and loss of *Pten* in melanocytes. When melanomas were visible (≅21 days, day 0), mice were randomized to D/T (*n* = 8) or D/T+nilotinib (*n* = 9). **g** Tumor sizes from **f** were scored on a scale of 1–9 on day 1 and day 105 and graphed as fold change in tumor burden. Box plots show the median (center line), interquartile range (box; 25th–75th percentiles), and whiskers extend to the minimum and maximum values. Datapoints are individual mice. *p*-value is from a two-sample, two-tailed *t*-test. Percentages of mice with tumor progression, defined as having large, thick, 3-dimensional tumors, are also shown. Supporting information for this figure can be found in Supplementary Fig. 4 (Kaplan–Meier curves) and Supplementary Fig. 5 (toxicity, animal weights). Genotypes and cell line names are indicated at the top of each graph.

Since ABL1/2 drive secretion of cytokines known to impact immune infiltration, we tested whether nilotinib prevents the onset of MAPKi resistance in immune-competent mouse models. Syngeneic models were established by injecting parental *Braf*- and *Nras*-mutant mouse melanoma cells into C57BL/6J mice, and mice treated with BRAFi/MEKi (D/T; *Braf*-mutant lines) or trametinib (MEKi; *Nras*-mutant lines) in the absence or presence of nilotinib when tumors were established (100–200 mm^3^). Importantly, nilotinib prevented MAPKi resistance from developing and increased survival in all syngeneic mouse models (Fig. 3a–d and Supplementary Fig. 4). Interestingly, OSUMMER.12 tumors were intrinsically resistant to trametinib (vehicle and trametinib curves were similar), which was efficiently reversed by addition of nilotinib (Fig. 3d). Nilotinib also reversed resistance that developed, in vivo (Fig. 3e). Finally, we tested the efficacy of nilotinib in a genetically engineered mouse model of *Braf*-mutant melanoma (*Braf/Pten*)^35^. Topical treatment of 4-hydroxytamoxifen causes replacement of the wild-type *Braf* allele with constitutively active *Braf* ^V600E^ and deletion of *Pten* in melanocytes, inducing melanoma development in ≈3 weeks^35^. Once melanomas were visible (treatment Day 0), male (9) and female (15) mice were randomized to D/T or D/T+nilotinib. Melanomas from D/T-treated mice initially regressed, but later developed resistance and progressed into large 3-dimensional tumors (Fig. 3f, g). In contrast, melanomas from D/T+nilotinib-treated mice either disappeared or remained small and flat (Fig. 3f, g). Importantly, no overt toxicity was noted in any of the mouse models (Supplementary Fig. 5). Thus, nilotinib effectively prevents and reverses resistance in immune-competent animal models.

### ABL1/2 inhibition prevents early Ly6C^hi^ and late Ly6G^+^ immune cell infiltration

**Fig. 4:**
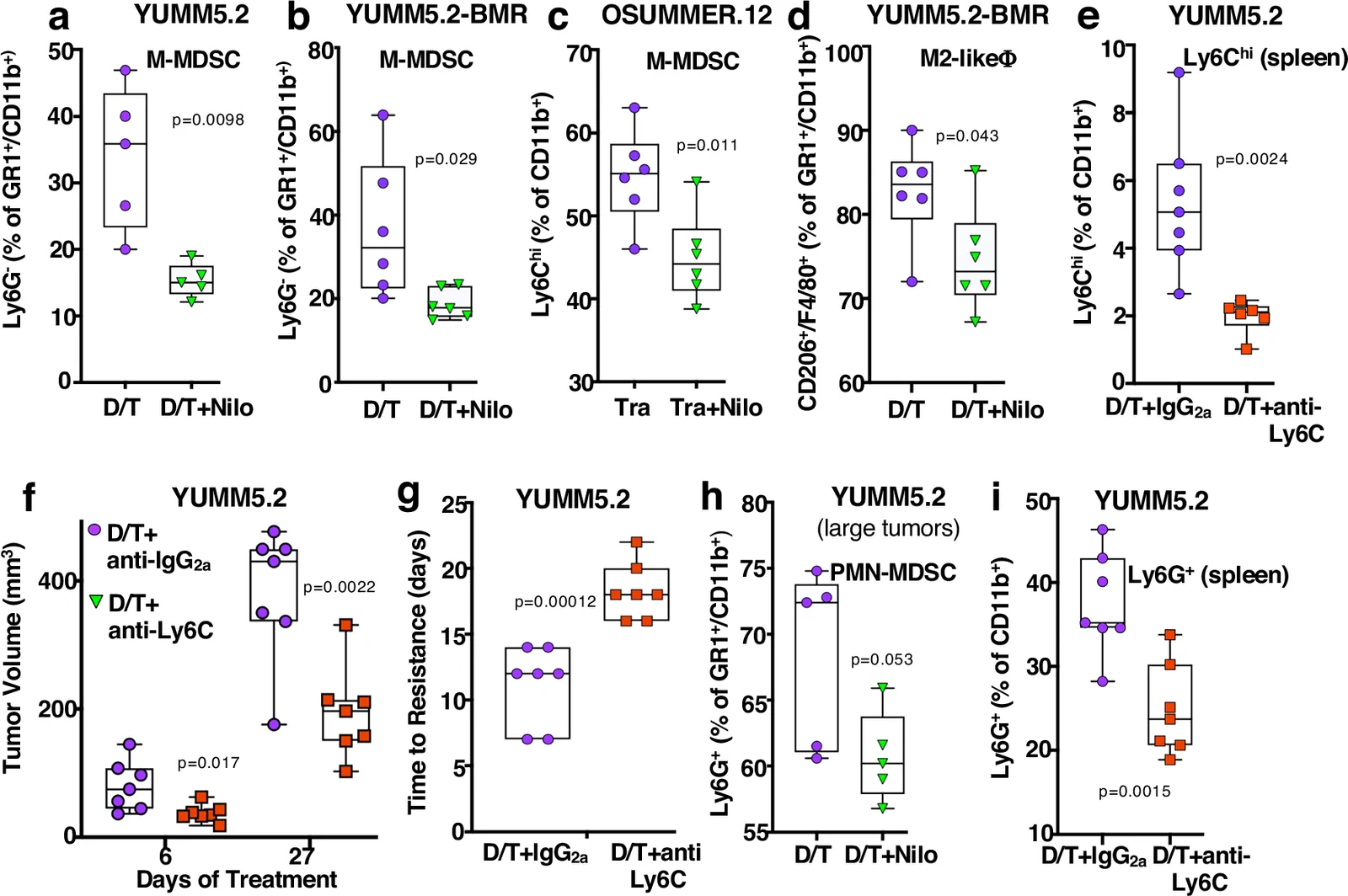
ABL1/2 drive MAPKi resistance by inducing cytokine secretion, which promotes MDSC infiltration. Male C57BL/6J mice (6–7 weeks of age) harboring small (100–150 mm^3^) tumors derived from **a** parental *Braf *-mutant YUMM5.2 cells, **b, d** BRAFi/MEKi-resistant YUMM5.2-BMR cells, or **c** female C57BL/6J mice harboring *Nras *-mutant OSUMMER.12 cells were randomized to D/T (*Braf*-mutant) or trametinib (*Nras*-mutant; Tra) or D/T+nilotinib (Nilo) or Tra+nilotinib for 4 days. Immune cells were isolated from the dissociated tumors and subjected to antibody staining and flow cytometric analysis. Gating strategies are in Supplementary Fig. 6a (YUMM5.2) and 6b (OSUMMER.12). **a**–**c** Monocytic cells and **d** M2-like macrophages (**Φ**) are shown. All percentages are a percent of the parent noted on the *Y*-axis. (**e**–**g, i**) Mice with YUMM5.2 tumors, treated with D/T+nilotinib, were injected with Ly6C or IgG_2a_ antibody every 3 days beginning 3 days prior to tumor cell injection. **e**, **i** Dissociated spleens were subjected to antibody staining/flow cytometry at the study endpoint. Gating strategy is in Supplementary Fig. 7i. **f** Tumor volumes 6 and 24 days after initiation of D/T treatment are shown. *p*-values are from two-tailed, two-sample *t*-tests. Tumor growth curves are shown in Supplementary Fig. 7f. **g** The time to develop resistance was estimated by recording the day at which tumors were at their smallest sizes (tumor growth accelerated after this day). *p*-values are from two-tailed, two-sample *t*-tests. **h** Ly6G^+^ cell tumor infiltration in mice harboring larger, more established YUMM5.2 parental tumors (≅350 mm^3^) treated with D/T or D/T+nilotinib. Tumor infiltrating lymphocytes were analyzed by flow cytometry. Gating strategy is in Supplementary Fig. 6a. Box plots show the median (center line), interquartile range (box; 25th–75th percentiles), and whiskers extend to the minimum and maximum values. Datapoints are individual mice. *p*-values are from two-sample *t*-tests (2-tailed). Representative flow cytometry dot plots are shown in Supplementary Fig. 7a–e, g, h. BMR BRAFi/MEKi-resistant, MR MEKi resistant, Nilo nilotinib, D/T dabrafenib/trametinib (BRAFi/MEKi).

Since ABL1/2 inhibition reverses and prevents resistance, and reduces secretion of cytokines known to promote an immunosuppressive tumor microenvironment, we examined whether nilotinib reduces tumor infiltration of immunosuppressive cells. In the presence of MAPKi, nilotinib treatment (4 days) of mice harboring small tumors reduced infiltration of GR1^+^/CD11b^+^/Ly6G^-^ (aka CD11b^+^/Ly6C^hi^ cells; inflammatory monocytes or M-MDSCs) as assessed by flow cytometry, and reduced M2-like macrophages in a BRAFi/MEKi resistance model (Fig. 4a–d and Supplementary Figs. 6 and 7a–d). Since nilotinib prevents resistance, the GR1^+^/CD11b^+^/Ly6G^−^ (CD11b^+^/Ly6C^hi^) population impacted by nilotinib is likely immunosuppressive (e.g., M-MDSCs). To confirm this hypothesis, we assessed the effect of depleting Ly6C^+^ cells on the onset of BRAFi/MEKi resistance. Depletion of Ly6C^+^ or Ly6G^+^ cells is notoriously difficult and is only partially effective, or the effects are transient due to rapid MDSC regeneration from the bone marrow and reduced expression of cell surface markers on immature cells, which leads to escape from depletion^36^. Indeed, depletion with Ly6C antibody only reduced Ly6C^hi^ cells by 50%; however, tumor growth in the presence of BRAFi/MEKi was reduced, and the time it took for tumors to develop resistance was significantly increased (Fig. 4e–g and Supplementary Fig. 7e, f).

In larger, more established tumors, nilotinib treatment reduced intratumoral granulocytic GR1^+^/CD11b^+^/Ly6G^+^ cells rather than M-MDSCs (Fig. 4h and Supplementary Fig. 7g; N1 neutrophils or PMN-MDSCs). Granulocytic cytotoxic N1 neutrophils initially infiltrate tumors, but later are replaced with immunosuppressive cells that promote tumor progression/growth (e.g., PMN-MDSCs)^14,15^. Interestingly, Ly6C depletion reduced the Ly6G^+^ population by ≅30% (Fig. 4i and Supplementary Fig. 7h, i), perhaps due to loss of conversion of Ly6C^hi^ M-MDSCs to PMN-MDSCs^15,17^, or reduced recruitment of Ly6G^+^ cells by Ly6C^hi^ cells^37^. Nonetheless, our data indicate that MDSCs promote BRAFi/MEKi resistance.

### ABL1/2 drive BRAFi/MEKi resistance by upregulating CXCL1, CCL2, and CCL5

**Fig. 5:**
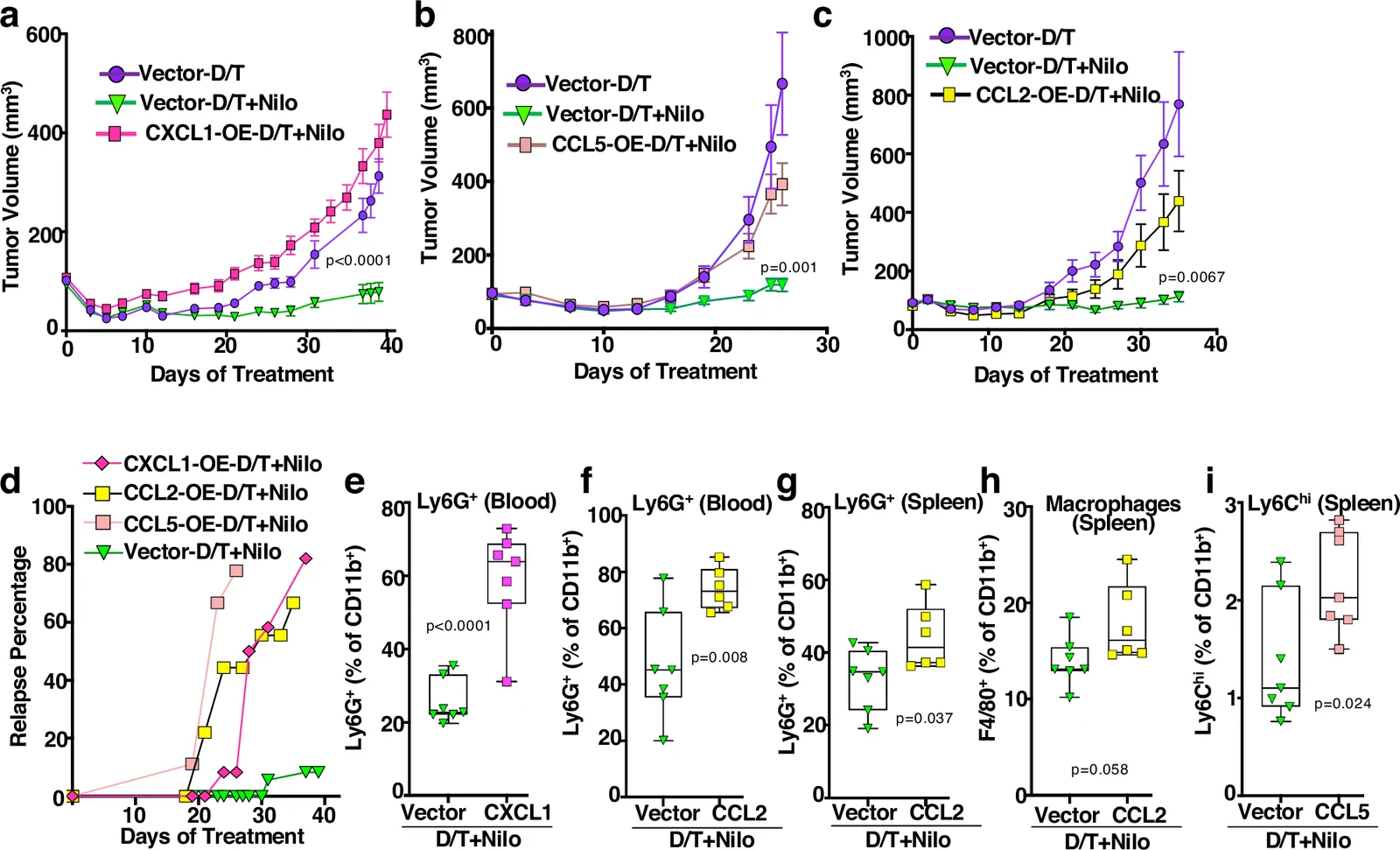
Exogenous CXCL1, CCL2, or CCL5 expression in melanoma cells blocks the ability of nilotinib to prevent the onset of BRAFi/MEKi resistance. **a**–**c** YUMM5.2 cells engineered to express vector or CXCL1, CCL2, or CCL5 (OE) were implanted into male C57BL/6J mice (6–7 weeks of age), and mice treated with D/T alone or D/T+nilotinib when tumors were established (100 mm^3^). Average tumor volumes over time are shown (Mean ± SEM). Number of animals utilized is as follows. **a** D/T, *n* = 7, D/T+Nilo-vector, *n* = 9, D/T+Nilo-CXCL1, *n* = 12. **b**, **c** *n* = 9/group. Individual tumor growth curves are shown in Supplementary Fig. 8d–f. **d** Percentages of mice that developed D/T resistance (relapse) defined as mice harboring tumors ≥200 mm^3^. **e**–**i** Antibody staining and subsequent flow cytometric analysis on peripheral blood and/or spleen from mice in **a**–**c** at the study endpoints. Percentages shown are a percent of the parent noted on the *Y*-axis. *p*-values are from two-sample, two-tailed *t*-tests. Representative flow cytometry dot plots are shown in Supplementary Fig. 8g, i–k. Additional immune populations are shown in Supplementary Figs. 8l–n, 9, and 10. Gating strategies are shown in Supplementary Fig. 8h, (blood) and Supplementary Figs. 7i and 10a (spleen). Box-and-whisker plots show the median (center line), interquartile range (box; 25th–75th percentiles), and whiskers extend to minimum and maximum values. Datapoints are individual mice. *p*-values are from two-sample *t*-tests (2-tailed). BMR BRAFi/MEKi-resistant, MR MEKi resistant, Nilo nilotinib, D/T dabrafenib/trametinib (BRAFi/MEKi). n.s. non-significant.

To determine whether ABL1/2 promote resistance and MDSC infiltration by driving chemokine/cytokine secretion, we tested whether constitutive, exogenous expression of CXCL1, CCL2, or CCL5 in the melanoma cells blocks nilotinib from preventing MAPKi resistance and reducing MDSCs. Notably, CXCL1, CXCL2, and CXCL5 all bind the CXCR2 receptor, and so, are predicted to have similar effects as CXCL1 alone. We found it difficult to express *Cxcl1* and were only able to obtain cells with low level overexpression (OE), whereas *Ccl2* and *Ccl5* expression was not counter-selected (Supplementary Fig. 8a, b). In the resulting stable cell lines, *Cxcl1*, *Ccl2*, and *Ccl5* expression only slightly increased the growth of the melanoma cells in vitro (Supplementary Fig. 8c), and did not prevent BRAFi/MEKi from inducing tumor regression (Fig. 5a–c, and Supplementary Fig. 8d–f; days 1–10). However, *Cxcl1, Ccl5*, and to a lesser extent, *Ccl2* expression blocked nilotinib from preventing BRAFi/MEKi resistance (Fig. 5a–d and Supplementary Fig. 8d–f). Tumors in the D/T+nilotinib control (vector) group were very small or non-existent (Fig. 5a–d). Thus, it wasn’t possible to perform flow cytometric analyses at the study endpoint, so instead we analyzed immune changes in the blood/spleen, which are indicators of MDSC migration and expansion^38,39^. In mice treated with DT+nilotinib, *Cxcl1* expression induced circulating CD11b^+^/Ly6G^+^ cells (Fig. 5e and Supplementary Fig. 8g, h; PMN-MDSCs), consistent with its known role in promoting granulocyte chemotaxis^15^. *Ccl2* expression also increased CD11b^+^/Ly6G^+^ immune cells in the blood and spleen, and increased splenic macrophages (Fig. 5f–h and Supplementary Figs. 8h–k and 7i). Ly6C^hi^ cells were either decreased or not significantly changed in mice harboring melanomas overexpressing *Cxcl1* or *Ccl2* (Supplementary Fig. 8l–n). In contrast to *Cxcl1* and *Ccl2*, *Ccl5* expression had no effect on CD11b^+^ cells in the blood (Ly6C^+^ or Ly6G^+^) and didn’t alter splenic Ly6G^+^ cells (Supplementary Fig. 9a–c), but instead increased splenic Ly6C^hi^ cells (Fig. 5i and Supplementary Fig. 9d), implicating CCL5 in M-MDSC chemotaxis and/or expansion. Thus, CXCL1 and CCL2 appear to primarily mediate Ly6G^+^ (PMN-MDSC) chemotaxis/expansion, whereas CCL5 increases Ly6C^hi^ cells (M-MDSC). Overexpression of *Ccl2* or *Ccl5* had no effect on splenic macrophages, NK cells, or B cells (Supplementary Figs. 9e and 10a–f), but *Ccl2* expression increased Tregs (Supplementary Fig. 10g). Thus, CCL2’s ability to rescue nilotinib’s effects may be mediated not only by effects on PMN-MDSCs but also via Tregs. Taken together, we show that nilotinib prevents MAPKi resistance by decreasing cytokine (CXCL1, CCL2, CCL5) expression, which reduces pro-tumorigenic immune cells.

### Nilotinib increases cytotoxic immune cell infiltration

**Fig. 6:**
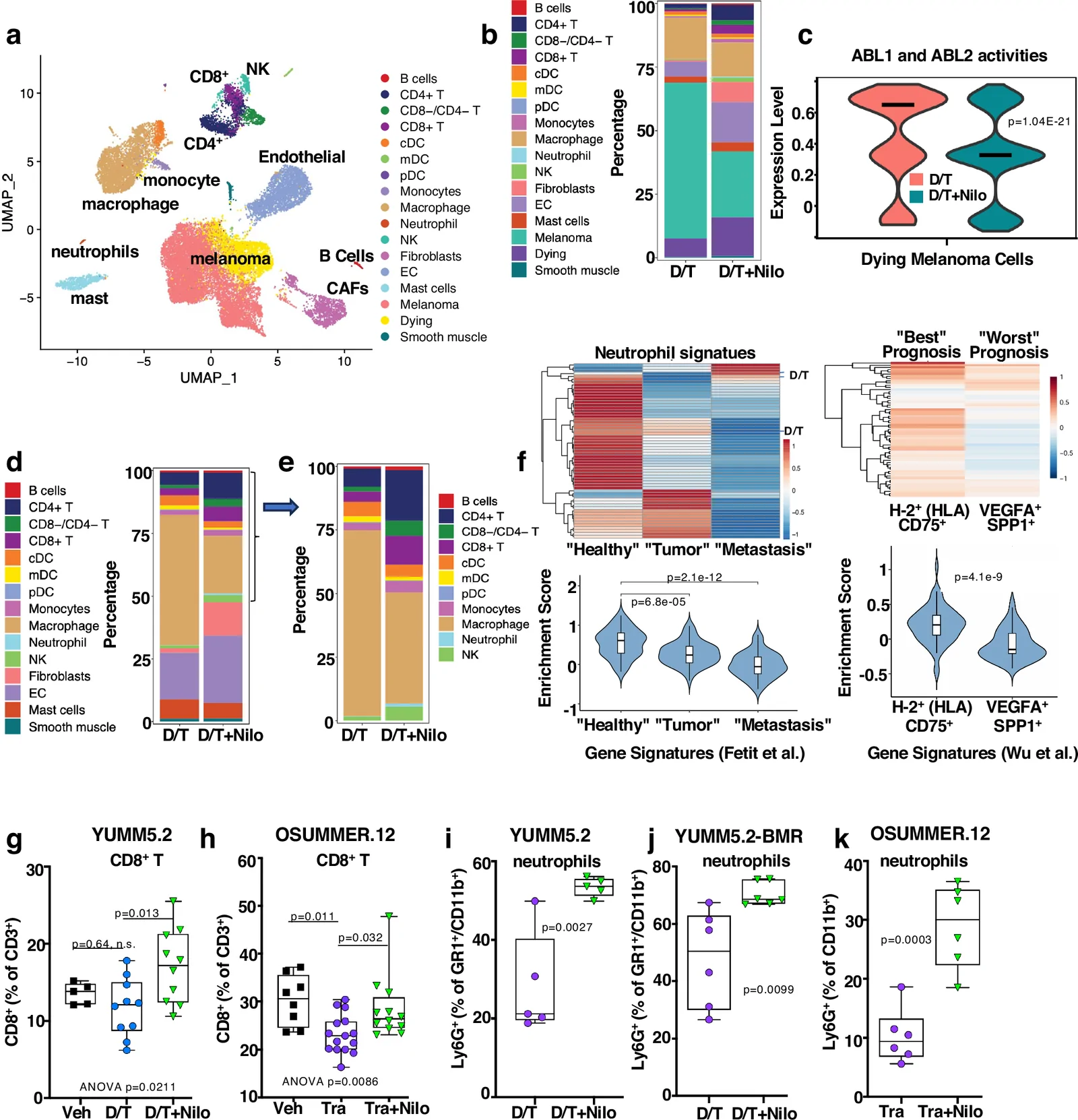
Nilotinib promotes cytotoxic CD8^+^ T cell infiltration. Male C57BL/6J mice (6–7 weeks old) harboring *Braf*^V600E^ parental YUMM5.2 tumors (50–100 mm^3^) were treated with D/T or D/T+nilotinib (see Fig. 3) for 4 days. Dissociated tumors were subjected to single-cell RNA sequencing (D/T = 10,251 cells; D/T+nilotinib=15,947 cells). Unsupervised cell cluster analysis is in Supplementary Fig. 11a, b. **a** UMAP showing identified cell types (includes both treatment groups). Genes that identify the clusters are in Supplementary Fig. 11c and Supplementary Dataset 2. **b** Quantitation of cell types in each group. **c** Gene module score analysis was used to estimate the combined activities of ABL1/2 (using the intersection of the genesets) in the dying melanoma population (Supplementary Fig. 11d, e). *p*-value is from 2-tailed Wilcoxon rank-sum test. **d** Cell distribution percentages normalized to tumor size. **e** Immune cell distribution. **f** Neutrophils were analyzed for gene expression signatures marking healthy (cytotoxic) neutrophils, tumor-associated, or metastasis-associated (pro-tumorigenic) neutrophils (left panels)^41^, and those associated with “best” and “worst” prognoses (right panels)^42^. Gene signature lists are in Supplementary Fig. 11f. Rows correspond to individual neutrophils, and columns represent curated gene signatures. Values in each cell are module scores that have been scaled per row (Z-score) to highlight the dominant signature for each individual neutrophil. Violin plots display the distribution of module scores across the cell populations. The center line indicates the median, boxes represent the interquartile range (IQR; 25th–75th percentiles), and whiskers extend to the most extreme values within 1.5 × IQR of the lower and upper quartiles. *p*-values are from two-tailed Wilcoxon rank-sum test and Bonferroni adjustment for multiple comparisons. **g**–**k** Infiltrating immune cells isolated from treated mice were subjected to antibody staining and flow cytometry. For **g**, **h**, values are from mice from two independent, pooled experiments. *p*-values are 2-tailed, two-sample *t*-tests. Box-and-whisker plots show the median (center line), interquartile range (box; 25th–75th percentiles), and whiskers extend to minimum to maximum values. Flow cytometry gating strategies are in Supplementary Fig. 6a (**g**, **i**, **j**) and Supplementary 6b (**h**, **k**). Representative dot plots are in Supplementary Figs. 12a, b, i–k. Other immune populations are in Supplementary Fig. 12c–h.

MDSCs play critical roles in suppressing the immune system, inhibiting chemotaxis, proliferation, and expansion of cytotoxic cells^15^. To test whether ABL1/2 impact cytotoxic cell infiltration, we first assessed the global effects of nilotinib on the immune microenvironment during resistance using single-cell RNA sequencing (scRNA-seq). Mice harboring small parental YUMM5.2 tumors were randomized to D/T or D/T+nilotinib treatment (4 days). Tumors were dissociated and subjected to scRNA-seq (using >10–15 K cells per treatment group). Unsupervised clustering analysis identified 31 unique cell clusters (Supplementary Fig. 11a, b), and the clusters were subsequently annotated into immune populations, melanoma cells, fibroblasts, and endothelial cells based on known marker genes (Fig. 6a, b). The top 5 markers that identify each cluster are in Supplementary Fig. 11c and Supplementary Dataset 2. The melanoma cells clustered into two distinct populations, one of which appeared to be dying, as the population had higher mitochondrial gene expression, lower overall gene expression, and activation of apoptotic signaling (Supplementary Fig. 11d, e and Supplementary Datasets 1, 3). Geneset-based module score analysis using the curated ABL1/2 gene set (intersection of ABL1 and ABL2 genes; Supplementary Dataset 1) demonstrated that dying melanoma cells from D/T+nilotinib-treated mice had reduced ABL1/2 activities compared with cells from mice treated with D/T alone (Fig. 6c). The distribution of non-malignant cell types, and specifically the immune populations, is shown in Fig. 6d, e. Nilotinib treatment increased cytotoxic immune cells, including CD4^+^, CD8^+^, λδT, and NK cells, and decreased immunosuppressive myeloid populations (e.g., tumor-associated macrophages-TAMs; Fig. 6e and Supplementary Dataset 3). Dendritic cells (DCs) were not significantly changed between treatment groups. MDSCs could not be separated from other myeloid populations due to the similarity in expression profiles among the myeloid cells, and published MDSC markers from a breast cancer model^40^ were not expressed in immune cells from this melanoma model. Interestingly, the neutrophil population was increased by nilotinib treatment (Fig. 6e). To determine whether this population is likely cytotoxic or protumorigenic (e.g., PMN-MDSCs), we subclassified the neutrophils based on previously reported gene signatures (Supplementary Fig. 11f and Supplementary Dataset 3)^41,42^. The majority of neutrophils, all but three of which came from D/T+nilotinib-treated animals, had a signature associated with classical (N1) neutrophils from healthy/normal tissue with cytotoxic function; HLA^+^ signature associated with the “best” prognostic outcome in human patients (Fig. 6f, Supplementary Fig. 11f, and Supplementary Dataset 3)^41,42^; and expressed markers for activated neutrophils (e.g., *Cebpb, Slpi*, Supplementary Dataset 3)^43^. Thus, scRNA-seq shows that nilotinib treatment increases cytotoxic T cells, NK cells, and potentially N1 neutrophils.

To confirm the scRNA-seq data, we performed antibody staining and flow cytometry (Supplementary Fig. 6). Four days of BRAFi/MEKi treatment in the *Braf*-mutant YUMM5.2 model did not alter tumor infiltration of cytotoxic CD8^+^ T cells (Fig. 6g, and Supplementary Fig. 12a). In contrast, MEKi treatment (4 d) reduced CD8^+^ T cells in mice harboring *Nras*-mutant melanomas (Fig. 6h and Supplementary Fig. 12b). Importantly, addition of nilotinib to the BRAFi/MEKi (*Braf*-mutant) or MEKi (*Nras*-mutant) regimen increased infiltration of cytotoxic CD8^+^ T cells (Fig. 6g, h and Supplementary 12a, b) but did not change B or NK cell infiltration (Supplementary Fig. 12c–h). A granulocytic GR1^+^/CD11b^+^/Ly6G^+^ population was upregulated by nilotinib treatment in small tumors (Fig. 6i–k and Supplementary Fig. 12i–k), indicating that this population may be cytotoxic (e.g., N1 neutrophils), which is consistent with the scRNAseq data demonstrating that nilotinib increases infiltration of neutrophils that express markers of activated neutrophils from healthy/normal tissue and a signature associated with the “best” prognosis (Fig. 6e, f, Supplementary Fig. 11f, and Supplementary Dataset 3)^41,42^. Thus, flow cytometry and scRNA-seq demonstrate that cytotoxic immune cells are increased in mice treated with nilotinib (in the presence of MAPKi).

### Nilotinib impacts infiltration of T cell subsets and CD8^+^ T cell transcriptional signatures

**Fig. 7:**
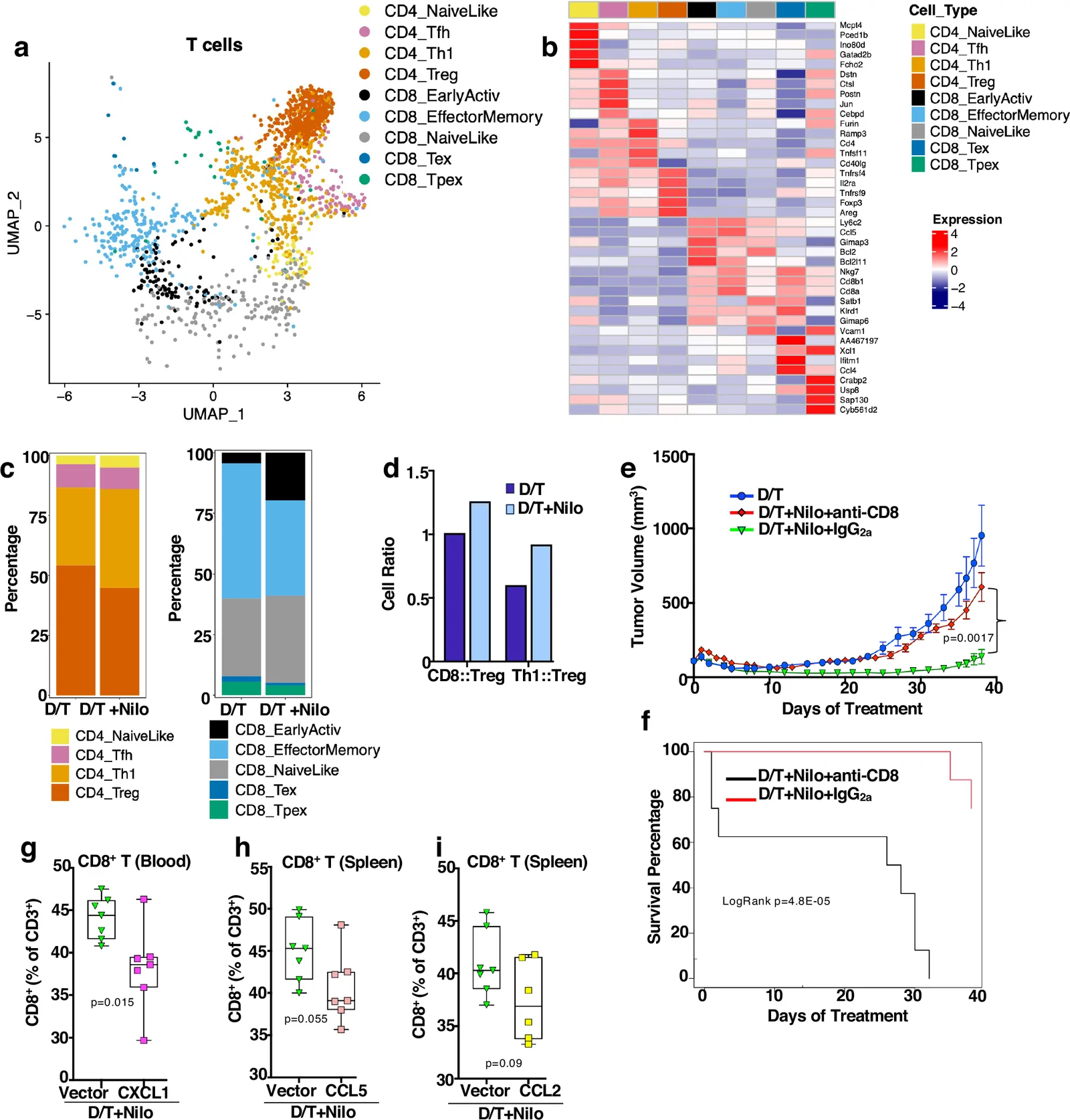
Prevention of MAPKi resistance by nilotinib requires cytotoxic CD8^+^ T cell infiltration. **a**–**c** T cell populations from single-cell RNA-seq (Fig. 6) were reclustered to identify CD4^+^ and CD8^+^ immune states^44^. EarlyActiv=early activated T cells; Tex=exhausted T cells; Tpex=precursers to exhausted T cells. Treg=T Regulatory Cells; Th1=T helper; Tfh=T follicular helper. Supplementary Fig. 13a shows the markers used for reclustering. **d** The ratio of Tregs to CD4^+^ and CD8^+^ T cells were plotted. Top genes and pathways changed in CD8^+^ T cells are shown in Supplementary Figs. 14,15. **e**, **f** Male C57BL/6J mice (6–7 weeks old) harboring YUMM5.2 parental tumors (100 mm^3^) were treated with D/T or D/T+nilotinib (*n* = 8/group) as described in Fig. 3. Mice treated with D/T+nilotinib were injected with anti-CD8 antibody or IgG control (200μg; i.p.; every 3–7 days beginning 3 days prior to tumor cell injection). D/T-treated animals injected with vehicle (PBS) were included as a control to be certain that tumors from mice treated with nilotinib+IgG were behaving as expected. Average (Mean±SEM) tumor growth curves are shown. Growth curves from individual mice are shown in Supplementary Fig. 16a. *n* = 8 mice/group. *p*-value is from a two-sample, two-tailed *t*-test at the endpoint. **f** Survival analysis using mice from the experiment in (**e**). Percentage of mice whose tumors did not reach the tumor doubling endpoint was used as the threshold. *p*-value is from a two-tailed LogRank test. The time to develop resistance was tabulated, and compared between groups in Supplementary Fig. 16b. CD8 depletion in an *Nras *-mutant model is shown in Supplementary Fig. 16c. **g**–**i** Antibody and flow cytometric analysis of CD8^+^ T cells in spleen or blood of mice harboring YUMM5.2 tumors exogenously expressing CXCL1, CCL2, or CCL5. Flow gating strategy is shown in Supplementary Fig. 7i (spleen) and Supplementary Fig. 8h (blood). Box-and-whisker plots show the median (center line), interquartile range (box; 25th–75th percentiles), and whiskers extend to the minimum and maximum values. Data points are individual mice. Representative flow cytometry dot plots are shown in Supplementary Fig. 16d–f. *p*-values are from two-tailed, two-sample *t*-tests.

To further examine the impact of nilotinib on T cell populations, we reanalyzed our single-cell RNA sequencing data, teasing out the types of T cells that are altered by nilotinib treatment using an algorithm described by Carmona and colleagues^44^. Various CD4^+^ and CD8^+^ states were identified (Fig. 7a–c and Supplementary Dataset 4)^44^. The top 5 genes that “mark” each T cell population are shown in Supplementary Fig. 13a, and expression of previously identified markers is in Supplementary Fig. 13b^44^. Early activated (effector) CD8^+^ T cells were increased in tumors from D/T+nilotinib-treated mice as compared to tumors from mice treated with D/T alone, and there were fewer CD8^+^ memory cells (Fig. 7c and Supplementary Dataset 5). Thus, nilotinib may prolong CD8-mediated effector function and delay death and differentiation^8^. Indeed, while not statistically significant due to small numbers, reduced exhausted populations also were observed in D/T+nilotinib tumors (Fig. 7c; 2-fold T_ex_ reduction, Supplementary Dataset 5). D/T+nilotinib tumors also had more CD4^+^ cytotoxic T helper cells (Th1)^9^, and reduced immunosuppressive Tregs (Fig. 7c and Supplementary Dataset 5)^13^. CD8:Treg and Th1:Treg ratios also were increased in tumors from nilotinib-treated animals (Fig. 7d), which is consistent with published data showing that low CD8^+^:Treg ratios are associated with melanoma progression and resistance to targeted therapy^7^.

To further dissect whether the CD8^+^ T cells are different in tumors from mice treated with nilotinib, we performed differential expression analyses and Gene Set Enrichment Analyses (GSEA). The top upregulated and downregulated genes changed by nilotinib treatment are shown in the heatmaps in Supplementary Fig. 14 (Supplementary Dataset 5). CD8^+^ T cells in nilotinib-treated animals had elevated expression of genes encoding growth factors involved in cell cycle progression (e.g., *Fos, Fosb, Jun, Klf4, Egr1*)^45,46^ and T cell activation and function (e.g., *Dusp1*, *s100a6*, *Fn1*; blue boxes; Supplementary Figs. 13c and 14a)^47–49^. Genes involved in T cell migration also were upregulated (e.g., *Ccn1*)^50^, as well as other genes known to play critical roles in cell migration of other cell types (extracellular matrix proteins-collagen, proteases-cathepsins, *Timp*s, *Dcn*, green boxes), but which have unknown/unclear roles in T cells (Supplementary Figs. 13c and 14a and Supplementary Dataset 5). Importantly, a re-examination of the T cell clustering data showed no contamination of CD8^+^ T cells with fibroblasts, endothelial, or smooth muscle populations, which are well-separated from T cell clusters (Fig. 6a). The majority of downregulated genes in tumor-infiltrating CD8^+^ T cells from mice treated with nilotinib were ribosomal genes (Supplementary Fig. 14b). These data are consistent with T cells being at peak expansion, where the majority of the cells are no longer dividing, and have decreased protein synthesis accompanied by reduced ribosomal gene expression^51^. Consistent with the above data, Gene Set Enrichment Analyses (GSEA) showed enrichment of pathways involved in proliferation (*MAPK, TNFA, RTK, KRAS* signaling), ameboid migration (integrin and non-integrin interactions, ECM formation/degradation, MET-induced motility), and repression of rRNA processing and translation elongation machinery in melanomas from nilotinib-treated animals (Supplementary Fig. 15 and Supplementary Dataset 6). In summary, nilotinib treatment (in the presence of MAPKi) alters the expression signature of intratumoral CD8^+^ T cells towards a more proliferative and migratory phenotype.

### CD8^+^ T cell infiltration is required for nilotinib to prevent MAPKi resistance

Since nilotinib increases intratumoral CD8^+^ effector T cells and potentially reduces exhausted populations, we tested whether CD8^+^ T cell infiltration is required for nilotinib to prevent the onset of BRAFi/MEKi resistance. CD8^+^ T cells were depleted from C57BL/6 J mice harboring YUMM5.2 tumors and treated with D/T+nilotinib. CD8^+^ T cell depletion, induced by injection of neutralizing antibody, blocked the ability of nilotinib to prevent resistance as compared to mice injected with IgG_2a_ (Fig. 7e, f and Supplementary Fig. 16a, b). However, the time to develop resistance was slightly longer in D/T+nilotinib+anti-CD8 animals as compared to animals administered D/T (Supplementary Fig. 16b). CD8^+^ T cells also were required for nilotinib to efficiently prevent MEKi resistance in a *Nras*-mutant model (Supplementary Fig. 16c). Thus, nilotinib’s ability to prevent resistance is highly dependent on CD8^+^ T cell function. To test whether the CCL/CXCL-MDSC axis is responsible for suppressing T cell infiltration during D/T+nilotinib treatment, we examined the effect of *Cxcl1/Ccl2/Ccl5* expression on circulating and splenic CD8^+^ T cells. Importantly, the number of circulating CD8^+^ T cells was reduced in melanomas expressing exogenous *Cxcl1* in mice treated with D/T+nilotinib, and there was a trend toward reduced splenic T cells in mice harboring melanomas expressing exogenous *Ccl2* and *Ccl5* (Fig. 7g–i and Supplementary Figs. 7i, 8h, and 16d–f). Taken together these data indicate that ABL1/2- driven chemokine secretion from melanoma cells promotes MDSC infiltration, which suppresses migration/expansion of cytotoxic CD8^+^ T cells.

### CXCL1 expression and ABL1/2 activities are elevated during BRAFi/MEKi resistance in patients

**Fig. 8:**
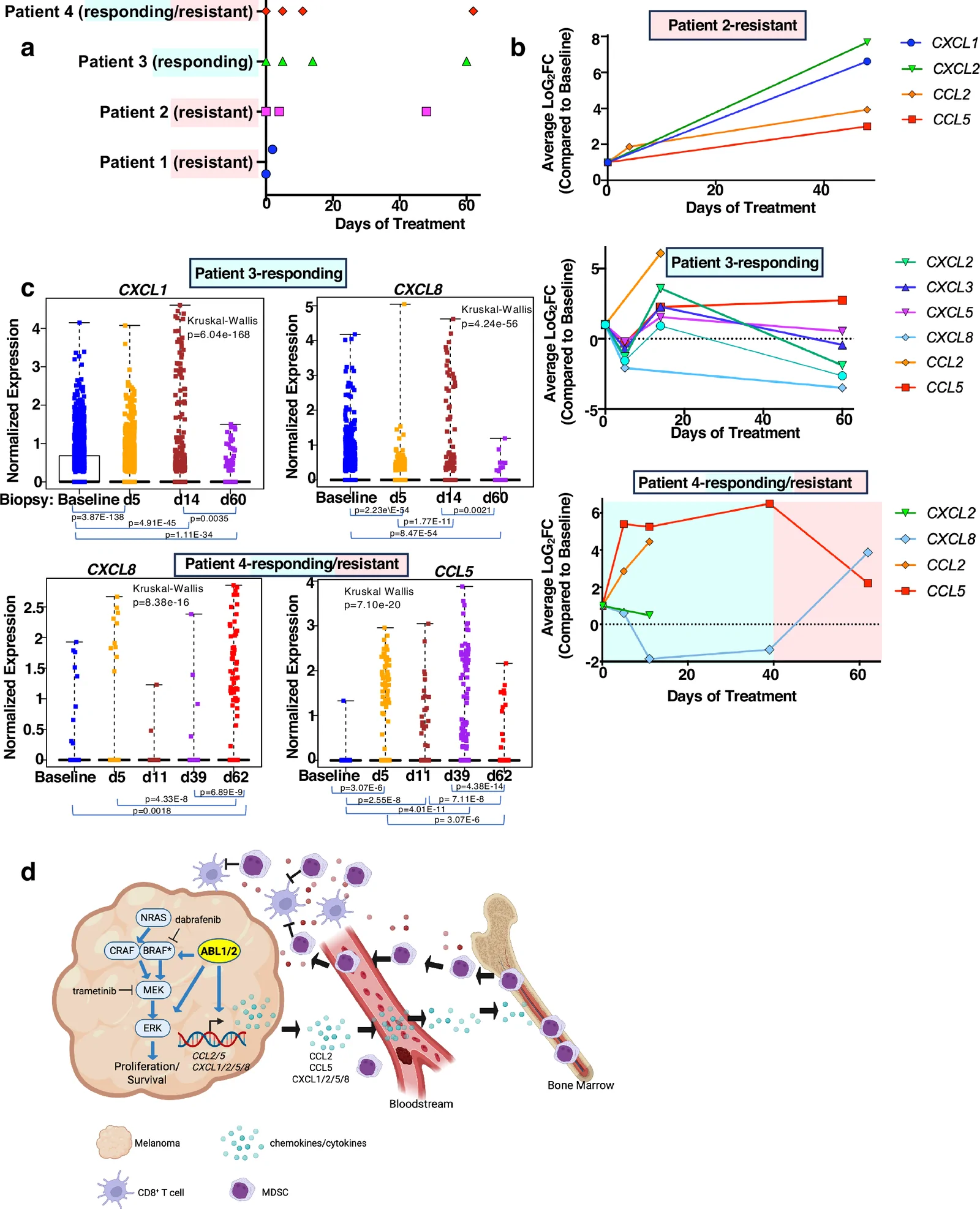
CXCR2 ligand expression is upregulated in melanomas from patients that develop resistance, reduced during treatment response, and elevated during resistance. **a**–**c** Re-analysis of single-cell RNA sequencing data (melanoma cells only) from Vasilevska et al. Patient Cohort 1. **a** Summary of repeated biopsies performed on each patient. **b** Differential expression analysis comparing post-treatment samples to baseline samples within each patient. Average log_2_FC values were plotted. **c** Box plots of gene expression in repeated biopsies. Box plots show the median (center line), interquartile range (box; 25th–75th percentiles), and whiskers extend to the minimum and maximum values. Individual points represent single cells. Overall differences in cytokine expression across timepoints were assessed with two-tailed, Kruskal–Wallis tests. Pairwise comparisons between timepoints were performed by two-tailed Wilcoxon rank-sum tests and adjusted with Holm’s method. **d** Summary model created in Biorender.com [Plattner, R. (2026) https://BioRender.com/414v3et]. In response to MAPKi, ABL1/2 drive re-activation of ERK signaling in melanoma cells and promote expression and secretion of chemokines. Secreted chemokines enter the bloodstream and travel to the bone marrow where they induce the migration of MDSCs that inhibit T cell migration, proliferation, and function, thereby contributing to melanoma cell growth and MAPKi resistance.

To determine whether the pathway we identified in vitro and in vivo also occurs in human patients, we first assessed how *CXCL1/2/8* and *CCL2/5* expression changes in melanoma patients during the course of BRAFi/MEKi treatment. Single-cell RNA sequencing (>45 K total cells) from melanomas from a previously described cohort of patients was reanalyzed, examining only the melanoma population^52^. Cohort 1 contains 4 patients who had repeated biopsies prior to and after BRAFi/MEKi treatment (Fig. 8a)^52^. Patient 1 had a resistant tumor that rapidly progressed (see Supplementary Table in reference)^52^. Patient 2 had a resistant nodule^52^. Patient 3’s tumor was slow to respond initially, but later had a complete response^52^. Patient 4 initially had a complete response but later progressed and was retreated with BRAFi/MEKi, which induced a partial response followed by rapid resistance^52^. Baseline mRNA expression of CXCR2 ligands was lower in melanoma cells from patients who developed resistance (Patients 1, 2) as compared to the responding patient (Patient 3), whereas baseline *CCL5* expression was elevated (Supplementary Fig. 17a and Supplementary Dataset 7). During BRAFi/MEKi treatment, CXCR2 ligand expression (CXCL1, CXCL8) was further reduced in melanomas from patients responding to treatment (Patient 3, Patient 4-d0-40), whereas *CCL5* expression remained constant (Fig. 8b, c and Supplementary Dataset 7). In contrast, during resistance (Patient 2, Patient 4-d40-60), CXCR2 ligand expression increased, whereas *CCL5* expression decreased (Patient 4) or was modestly elevated (Patient 2; Fig. 8b, c and Supplementary Dataset 7). Thus, baseline CXCR2 ligand expression was low in patients who eventually developed resistance, downregulated during treatment response, and upregulated during resistance. In contrast, baseline *CCL5* expression was elevated in patients who eventually developed resistance, upregulated during treatment response, and downregulated during resistance. Therefore, low expression of CXCR2 ligand mRNAs in conjunction with high *CCL5* mRNA may signal patients that will develop MAPKi resistance.

To determine whether ABL1/2 and DDR1 activities are modulated during treatment response and resistance, we first examined *ABL1/2* and *DDR1* mRNA expression in melanoma cells from the scRNA-seq data from Cohort 1. Since mRNA expression does not always indicate increased activity for highly regulated kinases, we did not expect to observe a change in mRNA expression. However, we did observe reduced *ABL2* mRNA during treatment response in a responding patient (Patient 3), and increased mRNA during resistance (Patient 2, Patient 4 d40-60; Supplementary Fig. 17b and Supplementary Dataset 7). In order to assess the activation status of nilotinib targets (ABL1, ABL2, DDR1) during resistance, which is more relevant than mRNA expression, we performed sample-wise gene set enrichment analysis. DDR1 activity was elevated during resistance in Patient 2, whereas ABL1 and/or ABL2 activities were reduced during response to treatment (Patient 3, Patient 4-d0-d40), and elevated during resistance (Patient 4-d40-60; Supplementary Fig. 17c and Supplementary Dataset 7). Thus, data with patient samples corroborates in vitro^5^ and in vivo data by demonstrating that ABL1/2 and DDR1 are activated during resistance, and this results in upregulation of chemokines that promote immune suppression during resistance.

## Discussion

Here, we demonstrate that ABL1/2 not only promote melanoma proliferation, survival, ERK reactivation, and protease secretion^5,6,19^, but also drive secretion of immunosuppressive chemokines from melanoma cells, which increases MDSC (and macrophage) tumor infiltration, resulting in reduced accumulation of cytotoxic CD8^+^ T cells during MAPKi resistance (Fig. 8d). Agents targeting ABL1/2, thus, have dual mechanisms for reversing and preventing resistance.

The role of immunosuppressive cells during MAPKi resistance is understudied. CCL2/CCL5 are thought to drive M-MDSC tumor infiltration, whereas CXCL signaling is thought to increase PMN-MDSCs^15,53^. We found that CXCL1 re-expression efficiently blocks nilotinib from preventing BRAFi/MEKi resistance, and increases circulating Ly6G^+^ cells (PMN-MDSCs) and reduces CD8^+^ T cells. These data are consistent with FACS data in larger established tumors showing that nilotinib reduces Ly6G^+^ cell infiltration. Moreover, consistent with our animal data, CXCR2 ligand mRNAs are reduced during BRAFi/MEKi treatment in patients and upregulated during resistance. Since nilotinib treatment also reduces immunosuppressive Ly6C^hi^ cells (M-MDSCs) in early tumors, we expected that ABL1/2 also drive resistance, in part, by promoting CCL2/CCL5 expression/secretion. Indeed, constitutive CCL2 or CCL5 expression also blocks nilotinib from preventing BRAFi/MEKi resistance, and CCL5 re-expression increases splenic Ly6C^hi^ cells. However, CCL2 increases circulating and splenic Ly6G^+^ cells similar to CXCL1 rather than increasing Ly6C^hi^ cells, and also increases splenic Tregs. While CCL2 is most known for its involvement in M-MDSC infiltration^54^, our data are consistent with colorectal cancer data showing that CCL2 drives PMN-MDSC chemotaxis/expansion^55^.

Single-cell RNA sequencing and flow cytometric analyses both demonstrate that nilotinib increases melanoma infiltration of cytotoxic CD8^+^ T cells. Nilotinib also changes the transcriptional profile of the CD8^+^ cells in the tumor microenvironment as genes/pathways involved in T cell activation, proliferation, and amoeboid migration are upregulated. Significantly, depletion studies demonstrate that CD8^+^ T cell infiltration is critical for nilotinib to prevent the onset of BRAFi/MEKi resistance in immune-competent mouse models. These studies contrast with other data showing that ABL1 activity is required for T cell development, proliferation, expansion, and function, in vitro and in vivo^56–59^. These opposing results are likely due to the former studies being performed in non-cancer mouse models which lack tumor-secreted chemokines that promote immunosuppressive cell infiltration. Here, we show that nilotinib treatment does not prevent T cell function in melanoma, but instead, enhances their infiltration by preventing melanoma chemokine secretion and subsequent MDSC infiltration. Future experiments will focus on further elucidating T cell and MDSC functions impacted by ABL1/2-driven cytokine secretion.

While T cell depletion blocks nilotinib from preventing resistance, the rescue was not 100% complete, indicating that other cytotoxic immune populations also contribute to nilotinib’s effects. Indeed, nilotinib increases infiltration of neutrophils with a “healthy” N1-like signature and “best” prognostic signature in small early tumors, although further experimentation is needed to confirm their cytotoxic function. Later, once tumors are more established, the granulocytic population, which is likely now immunosuppressive (PMN-MDSC), is reduced by nilotinib treatment. Interestingly, our single-cell RNA-seq data indicates that nilotinib also increases cytotoxic NK cell infiltration, but this finding could not be independently verified by flow cytometry. This discordant result may be due to differences in tumor dissociation methods, loss of NK cells during antibody staining, or alternatively, NK cells that are expanded by nilotinib treatment might not yet be mature enough to express the NKp46 marker^60^.

We and others showed that ABL1/2 promote expression, activation, and/or nuclear translocation of transcription factors^18,19,61^. Thus, future experiments will focus on identifying the mechanism by which ABL1/2 drive cytokine expression. Interestingly, mRNA expression of *CXCL2* in M14-BMR cells is driven by ABL1/2 in a kinase-independent manner, which is consistent with other literature indicating that ABL1/2 can signal independent of their kinase activities in some cell contexts^19,21–24^.

In summary, ABL1/2 not only induce pro-proliferative and pro-survival signals in MAPKi-resistant melanoma cells, but ABL1/2 also drive melanoma secretion of chemokines that drive immune suppression. We show in patients and animal models that CXCR2 ligands have a critical role in resistance to BRAFi/MEKi downstream of ABL1/2, and CCL2 or CCL5 also contribute to ABL1/2-driven immune suppression. While the CCL2/CCR2 axis has been implicated in promoting MDSC infiltration during BRAFi resistance^62^, and genetic or pharmacological targeting of CXCR2 reduces MDSC infiltration and enhances the efficacy of anti-PD-1 therapy^63,64^, our study also shows involvement of CXCL1 and CCL5 axes in BRAFi/MEKi resistance. Moreover, these data demonstrate that ABL1/2 serve as key nodes, driving targeted therapy resistance by promoting chemokine secretion and MDSC infiltration, and subsequently suppressing accumulation of cytotoxic CD8^+^ T cells. These findings are clinically significant as they demonstrate that ABL1/2 inhibition with nilotinib may be highly effective in reversing or preventing resistance due to their dual roles in overcoming immune evasion in addition to inhibiting melanoma proliferation and survival.

### Limitations of this Study

For CXCL/CCL overexpression studies, it was not possible to perform flow cytometric analyses at the endpoint, as tumors in the control animals were very small or non-existent. While evaluating MDSCs in the blood/spleen may be reflective of MDSC expansion, we recognize that MDSCs in these compartments may not behave the same as tumor-derived MDSCs.

## Methods

Catalog numbers and commercial sources for all reagents, cell lines, as well as computer programs described below can be found in Supplementary Table 1. Antibody dilutions for western blotting can also be found in Supplementary Table 1.

### Cell lines and reagents

Cell lines were grown in the following media: M14, DMEM/HEPES/10%FBS/10μg/ml insulin; YUMM lines, DMEM-F12/NEAA (non-essential amino acids)/10% FBS; OSUMMER lines, DMEM/5% FBS; MaNras -1007/1014, Ham’s F-12/HEPES/10% FBS; SK-MEL-2, RPMI 1640/10% FBS; SK-MEL-147, DMEM/10% FBS; WM164, L-15 (20%), MCDB153 (80%), insulin (5μg/ml), CaCl_2_ (168 mM), 2% FBS; and 293T in DMEM/10% FBS. *BRAF*-mutant BRAFi/MEKi resistant lines (BMR) were obtained by growing parental lines in increasing doses of dabrafenib/trametinib until resistant to 100 nM/20 nM. *NRAS*-mutant MEKi-resistant lines (MR) lines were similarly established until they were resistant to 20 nM trametinib. All resistant cell lines were maintained in drug-containing media.

#### Chemokine-overexpressing cell lines

Stable YUMM5.2 cell lines expressing CCL2, CCL5, or CXCL1 were obtained by lentiviral infection. Lv218-CCL2 (IRES2-mCherry-IRES-puro), Lv213-CCL5 (IRES-Neo), and Lv-220-CXCL1 (IRES-hygro) (see Supplementary Table 1) were transfected into 293T cells along with pRSV-Rev, pMD2.G, and pMDLg/pRRE plasmids at a 4:2:1:1 ratio using the calcium phosphate method. Briefly, 62 μl calcium chloride (2 M) was mixed with 16 μg of DNA, and distilled water (to 438 μL), 2X Hepes-Buffered Saline, pH 7.05 (0.5 ml) was bubbled into the calcium chloride/DNA, and the mixture was added dropwise to a 70% confluent 60 mm dish containing 0.1 mM chloroquine. After 5 h, the media was replaced with fresh medium and incubated for 48 h. Viral supernatants were filtered (0.45 μm) and used to infect YUMM5.2 cells using 8μg/ml polybrene for 16 h, followed by selection in puromycin (3 μg/ml), hygromycin (150 Units/ml), or G418 (350μg/ml). Surviving cells were pooled and expanded.

#### IPTG-inducible ABL1/2 shRNA

Cells expressing IPTG-inducible shRNA targeting *ABL1* and *ABL*2 (see Supplementary Table 1) were obtained following lentiviral infection and selection with puromycin (0.5 μg/ml). Clones were picked, expanded, and screened for knockdown by Western blot. Inducible shRNA-expressing cells were treated with IPTG (1 mM; 6 days) prior to screening.

#### Stable cell line expressing constitutively active ABL1 and ABL2

Due to toxicity of high level ABL1/2 overexpression, an inducible system was utilized. Constitutively active *ABL1* and *ABL2* (PP) were excised from their vectors (pSGT-*ABL1*-PP, BamH1^20^; PK1-*ABL2*-PP, EcoR1/XhoI^65^), blunted, and cloned into the Swa I site of the PiggyBac cumate-inducible transposable vector. Vector or *ABL1* and *ABL2* constructs were stably transfected into *NRAS*-mutant SK-MEL-147 or *BRAF*-mutant WM164 cells using Lipofectamine 2000, selected with puromycin (1 μg/ml), and clones pooled. Expression was observed in the absence (leaky) of cumate, but due to low level overexpression, no toxicity of ABL1/2 was noted.

### siRNA expression

siRNAs (see Supplementary Table 1) were transfected at the indicated concentrations (5–20 nM) using Lipofectamine 2000. Cells were lysed and RNA extracted for RT-qPCR 72 h after the transfection, or cells were serum-starved (0% serum) 16–24 h, and conditioned media used for cytokine arrays (see below).

### RT-qPCR

RNA was extracted from cells using RNAeasy kit (Qiagen). Primer sequences are in Supplementary Table 1. Primer-BLAST (National Center for Biotechnology Information website) was used to rule out homology to other genes. RNA was DNase-treated (ThermoFisher) and complementary DNA synthesized (50 ng; iScript, Bio-Rad) according to manufacturer’s instructions (see Supplementary Table 1). cDNA was amplified using SYBR green and gene-specific primers (500 nM; 40 cycles, 60 °C annealing temperature, CFX96, BioRad; Supplementary Table 1). Results were analyzed with CFX Manager 3.0, normalizing to the RPS13 reference gene.

### Western Blotting

Cells were lysed in RIPA buffer (50 mM HEPES pH7.0, 150 mM NaCl, 10% glycerol, 1% triton-X-100, 1.5 mM MgCl_2_, 1 mM EGTA, and fresh inhibitors--aprotinin/leupeptin/pepstatin-10μg/ml, 1 mM PMSF, 25 mM NaF, 1 mM sodium orthovandate). Equal amounts of cell lysate (BCA protein assay) were run on 8% SDS-PAGE gels and transferred to nitrocellulose. Antibodies and dilutions utilized for western blots are listed in Supplementary Table 1, and used according to manufacturer’s protocols. Cytokine western blotting involved resolution on tricine gels due to the small size of the proteins. Briefly, 15% acrylamide resolving gels incorporating 8% glycerol were run using Tris/Tricine buffer in the upper reservoir and Tris buffer in the lower reservoir.

### Single-Cell RNA sequencing

C57BL/6J mice harboring small YUMM5.2 parental tumors, treated for 4 days with D/T or D/T+nilotinib, were used for the study. Tumors from each group (*n* = 5) were pooled, dissociated using a MACS dissociator (mouse melanoma setting), red blood cells lysed, and dead cells removed (Miltenyi kits). Viability was assessed by Trypan Blue staining and counting on Countess II FL (Invitrogen). Library preparation, sequencing, and analysis were performed by the Markey Cancer Center Oncogenomics Shared Resource (OG SR). Live cells (D/T = 10,251; D/T+nilotinib = 15,947) were targeted for Gel Beads-in-emulsion (GEMs) formation on the 10X Genomics Chromium Controller. Library preparation was conducted according to the manufacturer’s instructions using Chromium Next GEM Single Cell 3’ Gene Expression Kit v3.1. Single-indexed, paired-end single cell libraries were sequenced on an Illumina HiSeq2500. Sequencing data were transferred to the Biostatistics and Bioinformatics Shared Resource (BB SR) for subsequent downstream data analyses (see below).

### Cell viability

#### Short-term

Cells were plated in quadruplicate in 96-well plates, drug-treated the following day, and harvested 72 h later with CellTiter Glo (see manufacturer’s instructions, Supplementary Table 1).

#### Long-term

Clonogenic assays were performed by plating cells (500–2000) in 6-well dishes in the presence of trametinib (or D/T), treating with DMSO (vehicle) or drugs (nilotinib, DDR-IN-1) for 7 days, cells washed, and grown in trametinib (or D/T) alone until colonies were evident. Plates were washed with PBS, fixed in 2% paraformaldehyde, stained with 0.05% crystal violet (in 50% methanol), washed with water, and allowed to dry. Plates were imaged on a Cytiva/Amersham 600 RGB Imager.

### Cell cycle analysis

Cells were plated and serum-starved (identical confluency and conditions as used for cytokine arrays) or left in serum in the same conditions and density used for RT-qPCR. Cells were trypsinized, fixed in 70% ethanol, rinsed in PBS, and resuspended in propidium iodide/RNase staining solution (Supplementary Table 1). Cells were subjected to flow cytometry using a BD Symphony A3 Flow Cytometer, and data recorded using FACSDiva software (V9.1). Cell cycle distribution was determined using the Watson model in FlowJo_v10.10.0 software. Since the percentages in FlowJo cell cycle analysis can slightly exceed 100% due to mathematical modeling constraints, specifically the use of Gaussian distributions to fit peaks for G0/G1, S, and G2/M phases, the numbers were converted to reflect a portion of 100%.

### ELISA

YUMM5.2 cells expressing vector or CXCL1 were plated at 80% confluency. The next day, cells were serum-starved (0% serum) for 20–24 h, media spun down, filtered through a 0.2μM filter, diluted 1:2, and CXCL1 concentration determined using an R&D DuoSet ELISA kit using the Duo Ancillary Reagent kit (see Supplementary Table 1), following the manufacturer’s instructions.

### In vivo studies

#### Syngeneic mouse models

C57BL/6J mice (JAX Stock: #000664, 5–6 weeks of age, Bar Harbor, MN) were injected subcutaneously with mouse syngeneic cell lines [*Braf*-mutant: YUMM5.2 (4 × 10^5^), YUMM1.7 (4 × 10^5^), YUMM3.3 (3 × 10^5^); *Nras-*mutant: OSUMMER.12 (1 × 10^5^)]^30,32^. In order to prevent rejection, male mice were utilized for lines established from male models (YUMM5.2, YUMM1.7) and female mice used for lines established from female mouse models (YUMM3.3, OSUMMER.12)^30,32^. When tumors reached 100–200 mm^3^, mice were randomized to treatment groups. Dabrafenib (20 mg/kg/d) and trametinib (0.1–0.15 mg/kg/d) were administered by oral gavage once daily, whereas, nilotinib was administered by oral gavage twice daily (84 mg/kg/d). Drugs were dissolved in the following vehicle: DMSO:PEG400:Vitamin E TPGS: Poloxamer 407 (Pluronic F127): PBS-1X at a 5:25:10:1:59 ratio. Doses of dabrafenib and trametinib (BRAFi, MEKi) for each experiment are indicated in the figure legends. For single-cell RNA sequencing and flow cytometric experiments, mice were drug-treated for 4 days.

#### Genetically engineered mouse model

Fifteen female and nine male *Braf/Pten*   mice (B6.Cg-Tg(Tyr Cre/ERT2) 13Bos *Braf* ^*tm1Mmcm*^ *Pten*^*tm1Hwu*^/BosJ; JAX Stock: #13590, 6–7 weeks old) were treated topically on their back with 4-hydroxytamoxifen (1–2 μl of 5 mM)^35^. When melanomas were visible (≅21 days), mice were randomized to dabrafenib/trametinib (D/T) or D/T+nilotinib (D/T: 6 female, 5 male; D/T+nilotinib: 9 female, 4 male), and treated as described above. Mice were euthanized when tumors developed D/T resistance and became 3-dimensional (105 days of treatment).

For all models, mice were monitored daily, tumors were measured with digital linear calipers at the indicated time points (every 1–3 days), and volumes calculated (LxW^2^/2). Mice were euthanized by carbon dioxide asphyxiation followed by cervical dislocation when tumor volumes reached a maximum size of 1500 mm^3^ (approved by the University of Kentucky Institutional Animal Care and Use Committee (IACUC; animal welfare committee) or at a prior point if tumors became ulcerated (humane endpoints; adhered to for all studies). All animals were housed in a pathogen-free/germ free/GF environment, with experimental and control animals co-housed. Experiments were approved by the University of Kentucky animal ethics/welfare (IACUC) committee under protocol #2020-3426 with the most recent approval of 3/20/2026.

### Cytokine array screening

Human and mouse cytokine membrane arrays (C1000) containing antibodies to 120 or 96 cytokines, respectively, were purchased from RayBiotech. Conditioned media from serum-starved (0% serum), drug-treated cells (12–16 h; conditioned media from equal cell numbers) were incubated with the array overnight at 4 °C as described in the manufacturer’s directions (see Supplementary Table 1). Following ECL, the membranes to be compared (e.g., D/T versus D/T+nilotinib) were placed together in a cassette and exposed to film for various exposures ranging from 10 s to 5 min. All film exposures were in the linear range for ECL (<20’), and membranes to be compared were exposed to film simultaneously. Duplicate cytokine spots were quantitated with Image J and divided by quantitated control spots. The corresponding data were then normalized to the vehicle, vector, or non-targeting control and expressed as a percentage of the control.

### Antibody staining/flow cytometric analysis

Melanomas from C57BL/6J mice harboring *Braf*-mutant YUMM5.2 or *Nras*-mutant OSUMMER.12 tumors (see above), treated with D/T or trametinib versus D/T+nilotinib or trametinib+nilotinib for 4 days, were harvested from euthanized mice, tumors fragmented, dissociated in digestion cocktail (Type IV collagenase-33.3 mg/ml, haluronidase-3.3 mg/ml, Type I DNase-0.33 mg/ml) for 1 h at 37 °C, and passed through 70 μM nylon mesh. Single cell suspensions were washed with PBS, cells counted (TC20, BioRad), incubated in Fc blocking antibody (CD16/CD32 antibody cocktail) and fixable live/dead stain (see Supplementary Table 1) for 15’ at 4 °C. Antibody cocktails were added (see Supplementary Table 1 and Supplementary Figs. 6, 7i, 8h, and 10a) and incubated for 45’. Following washing, cells were fixed for 20’ at 4 °C, resuspended in FACS buffer, and analyzed on BD Symphony (YUMM5.2) or Cytek Aurora (OSUMMER.12, blood and spleen samples), gating 0.1–1 × 10^6^ live events or all events. Gating strategies and antibody tables are shown in Supplementary Figs. 6, 7i, 8h, and 10a. Peripheral blood was removed from some animals by cardiac puncture in the presence of 0.5 M EDTA. Red blood cells were removed (Miltenyi kit) and lymphocytes stained as above using the gating strategy in Supplementary Fig. 8h. Mechanically dissociated splenocytes from other experiments were stained using the gating strategies in Supplementary Figs. 7i and 10a. All data were recorded using SpectroFlo 3.3.0 and analyzed using FlowJo_v10.10.0 software.

### In vivo depletion

Anti-CD8, anti-Ly6C, or IgG_2a_ isotype control (200 μg; i.p.; Supplementary Table 1) were injected 3 days prior to injection of melanoma cells and every 3rd day throughout the study, except for the anti-CD8 experiments where the antibodies were injected every 3 days for the first two weeks and every 7 days thereafter.

### Patient dataset

The single-cell RNA sequencing dataset obtained from Cohort 1, previously described in Vasilevska et al.^52^, was reanalyzed for this manuscript. Fine-needle biopsies obtained from patients prior to and during BRAFi/MEKi treatment were subjected to single-cell RNA sequencing. Tumors/patients were classified as “responding” (Patient 3) if their melanoma disappeared during therapy, and termed “resistant” (Patients 1, 2) if the tumor did not completely regress. For Patient 4, the tumor disappeared, and then returned later after subsequent treatment.

### Statistical analyses

#### In vitro and in vivo studies

One-Way Analysis of Variance (ANOVA; >2 comparisons), two-sample (comparisons between two groups), or one-sample *t*-tests (comparisons to normalized controls) were used to analyze in vitro experiments (using GraphPad Prism or Vassar Website). The Holm’s method for multiple comparisons adjustment was used for one- and two-sample tests, and Tukey’s multiple comparisons test used for one-way ANOVAs. In vivo studies were analyzed with the linear mixed model (SAS, v9.4) to compare log-transformed tumor volumes between treatment groups. For survival analyses, Kaplan-Meier and Log-Rank tests (R, v4.3.1) were used to compare time to tumor doubling. In all cases, two-tailed *p* values were reported (*p* < 0.05 was considered statistically significant).

#### Sample-wise gene set enrichment analysis

The following datasets were utilized for the analyses. 1) TCGA melanoma database (*n* = 471); 2) samples from patients with *NRAS*-mutant melanomas treated with MEKi (*n* = 23; GSE198776)^28^; and 3) samples from patients with *BRAF*-mutant melanomas treated with MAPKi (GSE65185)^29^, using only samples from patients demonstrating resistance to the MAPKi regimen, and removing samples from patients who were not treated with MAPKi or were sensitive to the regimen. Moreover, replicates from the same patients were removed by taking an average, leaving a final sample size of *n* = 24. Sample-wise gene set enrichment analyses methods, such as those implemented in Gene Set Variation Analysis (GSVA v1.50.0) package, transform gene expression data into pathway-centric scores at the single-sample level, where an enrichment score is computed to quantify the pathway activity for each pathway in each sample. Using GSVA and the Ingenuity Pathway Analysis (IPA) database (genes and their associated downstream targets integrated from published data; Supplementary Dataset 1)^26^, we calculated enrichment scores to quantify kinase activities in individual melanomas based on RNA-seq data, where a high positive enrichment score indicates that the target gene set (kinase activity) is highly upregulated within a particular sample. The calculated enrichment scores were then used for Spearman’s correlation analyses to assess the correlations between ABL kinase activities and cytokine mRNA expression. Some of the targets in the IPA genelists utilized for performing the single-sample enrichment analysis (see Supplementary Dataset 1) were validated by RT-qPCR using M14-BMR cells engineered to express either non-targeting shRNA (shNT) or shRNA targeting ABL1 and ABL2 (see Supplementary Table 1).

#### Single-cell RNA sequencing (scRNA-seq)

Single-cell gene expression libraries were mapped to the mm10 mouse reference (10X Genomics pre-built reference, mm10-2020-A) using Cell Ranger (v6.0.0, 10X Genomics). For each scRNA-seq library, the Cell Ranger-filtered matrix was subjected to QC filtering. Based on the distribution of library complexity, low quality cells with ≤200 expressed genes, ≤500 unique molecular barcodes, or ≥ 20% mitochondrial transcripts were removed. Doublets were detected and filtered using scDblFinder (v1.14.0)^66^ with default parameters. Cells that passed the QC from D/T and D/T+nilotinib groups were integrated using Seurat (4.4.0) SCT-based integration workflow^67^. Each dataset was individually normalized and scaled using the SCTransform function^68^. The top 2000 most variable features across datasets were subsequently used to identify integration anchors and merge the datasets into an integrated object. The shared nearest neighbor (SNN) graph was constructed based on the first 50 principal components. Cell clusters were then identified by applying unsupervised clustering using FindClusters function with Louvain algorithm (resolution 0.5), resulting in 31 clusters. The Uniform Manifold Approximation and Projection (UMAP) on the 50 principal components was generated for a two-dimensional embedding of the integrated scRNA-seq datasets. Cluster marker genes and differential gene expression analyses between the two experimental groups was performed using a Wilcoxon rank-sum test. The cell types were annotated according to expression of canonical markers in Supplementary Fig. 11c. Malignant melanoma cells were identified based on widespread chromosomal amplification and deletion patterns inferred using InferCNV (1.20.0) (Supplementary Fig. 11d). Dying melanoma cells were defined as melanoma cells with copy number variations, and an increased apoptosis-like state as indicated by elevated activity of apoptosis-related pathways (Supplementary Fig. 11e). Gene signatures from healthy, tumor-associated, or metastasis-associated neutrophils were obtained from Fetit et al.^41^. Signatures for HLA-DR^+^CD74^+^ and VEGFA^+^SPP1^+^ neutrophils^42^ were derived from the human pan-cancer neutrophil atlas, defined as the top 20 marker genes of each cluster. Human gene symbols were converted to mouse orthologs using babelgene (https://github.com/igordot/babelgene). To quantify the activity of specific gene signatures, we calculated the module scores at the single cell level using the AddModuleScore function. Gene set enrichment analysis was performed using the fgsea (1.20.0) R package^69^ based on the ranked gene list from the differential analysis. The two-sample binomial test was used to compare the distribution of cell clusters between the 2 groups. T cell subtype annotation. To assess differences in T cell states between D/T and D/T+nilotinib treatments, we projected our scRNA-seq data onto a mouse tumor-infiltrating lymphocyte (TIL) reference atlas using the ProjecTILs R package (v3.0)^44^. The reference atlas defines nine distinct T cell states based on murine tumor scRNA-seq data. A normalized expression matrix was used as input, and non-T cells were excluded prior to projection. Query cells were aligned to the reference UMAP embedding using the make.projection function with default parameters, and T cell states were assigned via cellstate. predict using a nearest-neighbor classifier. Projected cells were visualized on the reference UMAP using plot.projection. T cell annotations were further validated by examining the expression of markers defined by the projectTILs reference atlas.

#### Analysis of scRNA-seq from patient samples from Vasilevska et al (GSE229908)^52^

For each patient, pairwise differential expression analysis was performed for each post-treatment biopsy and baseline biopsy on the tumor cells using the findMarkers function from scran^70^. Gene Set Variation Analysis (GSVA, v1.50.0) enrichment scores were computed based on the normalized scRNA-seq expression of the downstream target gene sets of ABL1, ABL2, and DDR1 within each patient, treating each malignant cell as a sample. Box plots of the computed GSVA enrichment scores were generated to compare the activities of ABL1, ABL2, and DDR1 among the timepoints. Overall differences in GSVA enrichment scores for ABL1, ABL2, and DDR1 across timepoints were assessed using non-parametric Kruskal–Wallis tests. When the overall test was significant, pairwise comparisons between timepoints were performed using non-parametric Wilcoxon rank-sum tests. Resulting *p*-values were adjusted for multiple comparisons using Holm’s method.

### Reporting summary

Further information on research design is available in the Nature Portfolio Reporting Summary linked to this article.

## Supplementary information

Supplementary Information

Transparent Peer Review file

Reporting Summary

Description of Additional Supplementary Files

Supplementary Data 1

Supplementary Data 2

Supplementary Data 3

Supplementary Data 4

Supplementary Data 5

Supplementary Data 6

Supplementary Data 7

## Source data

Source data

## Data Availability

Single-cell RNA sequencing analyses generated in this manuscript are included as Supplementary Material. The raw single-cell RNA sequencing files have been deposited to Gene Expression Omnibus (GEO) under accession number GSE325262. TCGA-SKCM gene expression data were obtained from GDC portal in Nov. 2019, and the corresponding clinical data were downloaded from cBioPortal. MEKi-treated *NRAS*-mutant melanomas (Nagler et al.) were obtained from Adi Nagel and Yardena Samuels in Dec. 2023. The data were originally deposited as part of a larger RNA sequencing project by collaborator, Dr. Phil Cheng (GSE198776) [https://www.ncbi.nlm.nih.gov/geo/query/acc.cgi?acc=GSE198776]. Data from patients with *BRAF*-mutant, MAPKi-resistant tumors from GSE65185 data were downloaded (GEO) in 2019. Data from Vasilevska et al., provided by authors, Drs. Cheng and Levesque, have been deposited in GEO under accession number GSE229908. Source data are provided with this paper.
