# Supplementary material for: Targeting ABL kinases overcomes melanoma immune evasion following resistance to MAPK inhibition
Source: Nat Commun. 2026 Aug 21;17:9995. doi: 10.1038/s41467-026-76864-y (PMC13590603; doi:10.1038/s41467-026-76864-y)
Supplement: Supplementary file 1 [file 41467_2026_76864_MOESM1_ESM.pdf]

SUPPLEMENTARY MATERIALS

Targeting ABL kinases overcomes melanoma immune evasion following resistance to MAPK inhibition

Rakshamani Tripathi,[1] Bhuvanesh Sukhlal Kalal,[1] Shirin Soni,[1] Chezney Boothe,[1] Anastasia Lyon,[1] Christina Meeks,[1] Daheng He,[2] Phil Fang Cheng,[3] Mitchell P Levesque,[3] Jinpeng Liu,[2] Chi Wang,[2] and Rina Plattner[1*]

This PDF file includes:

Supplementary Figures

Supplementary Table

Legends for Supplementary Dataset Files

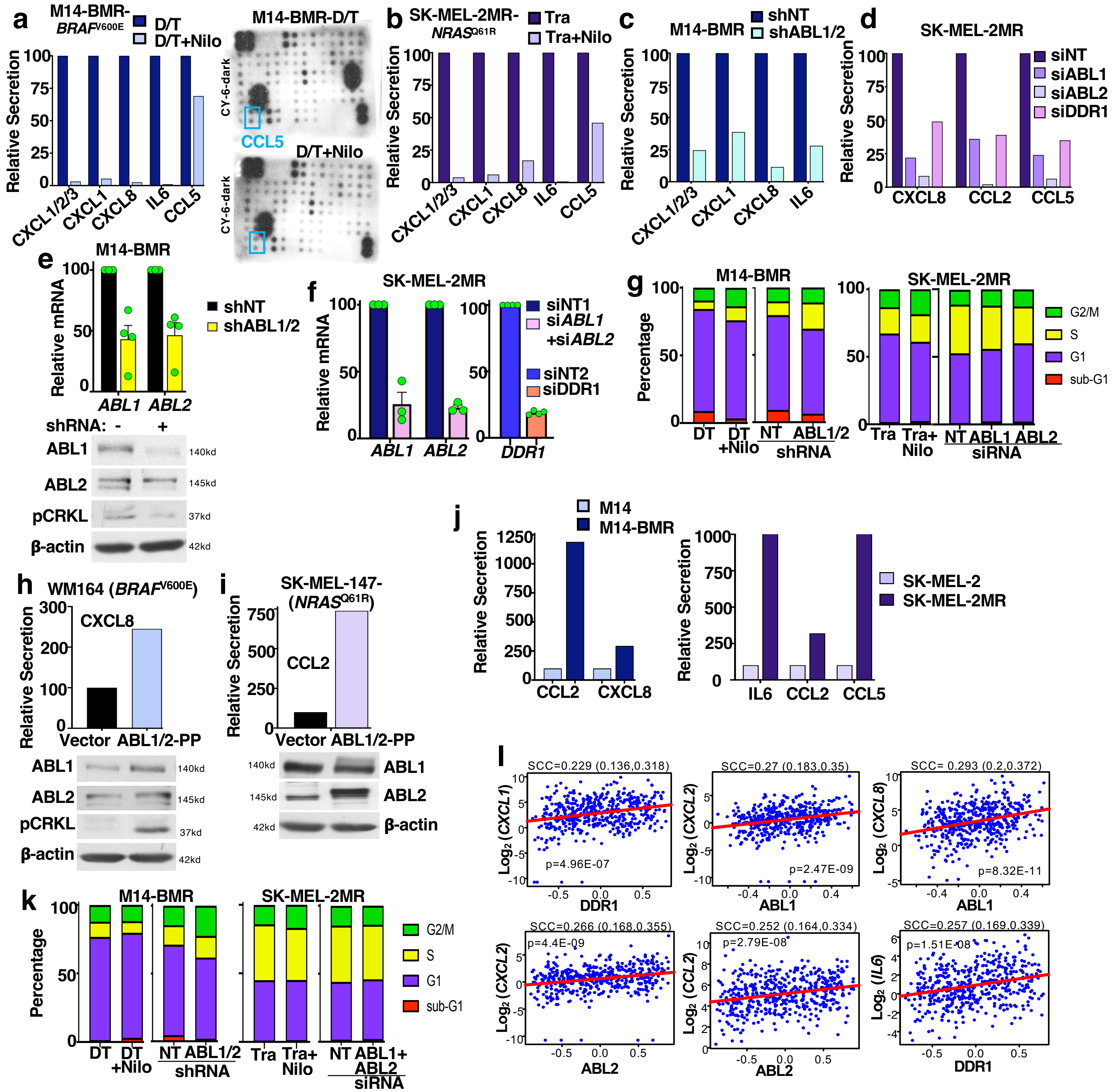

Supplementary Figure 1. ABL1/2 and DDR1 kinases promote cytokine secretion and expression. Supporting data for Figs. 1,2. **(a-left, b-d, h-top, i-top, j)** Graphs: ImageJ64 quantitation of secreted cytokines from membrane arrays shown in Fig. 1. Spots were quantitated with ImageJ64 and expressed relative to control spots. **(a-right)** Dark exposure of the cytokine array-CYT6 from Fig. 1a. **(e,f, h-bottom, i-bottom)** Supporting data for Fig. 1c-e; 2c-e. RT-qPCR and western blots showing knockdown efficiency of shRNAs and siRNAs and expression of ABL1/2-PP by western blot. Graphs are mean±SEM for n=3 independent experiments (biological replicates). siRNAs were utilized at 10nM. pCRKL (ABL1/2 substrate) is a readout of ABL1+ABL2 activity. For h,i, cells were in serum-starved conditions. **(g,k)** Cell cycle (propidium iodide staining) analysis of treated cells in serum-starved **(g)** or serum conditions **(k)**. Results are representative of 3 independent experiments (see Source File). **(l)** Supporting data for Fig. 2i. Sample-wise geneset enrichment analysis of RNA sequencing data from the melanoma TCGA database (n=471). Kinase enrichment scores ("activities") were correlated with cytokine mRNAs using Spearman's Correlation Coefficient. Correlation coefficients (SCC), 95% confidence limits, and 2-tailed p-values are shown. D/T=dabrafenib/trametinib (BRAFi/MEKi); BMR=BRAFi/MEKi-resistant; MR=MEKi resistant; Nilo=nilotinib; NT=non-targeting; PP=constitutively active ABL1/2; DDR1=Discoidin Domain Receptor 1.

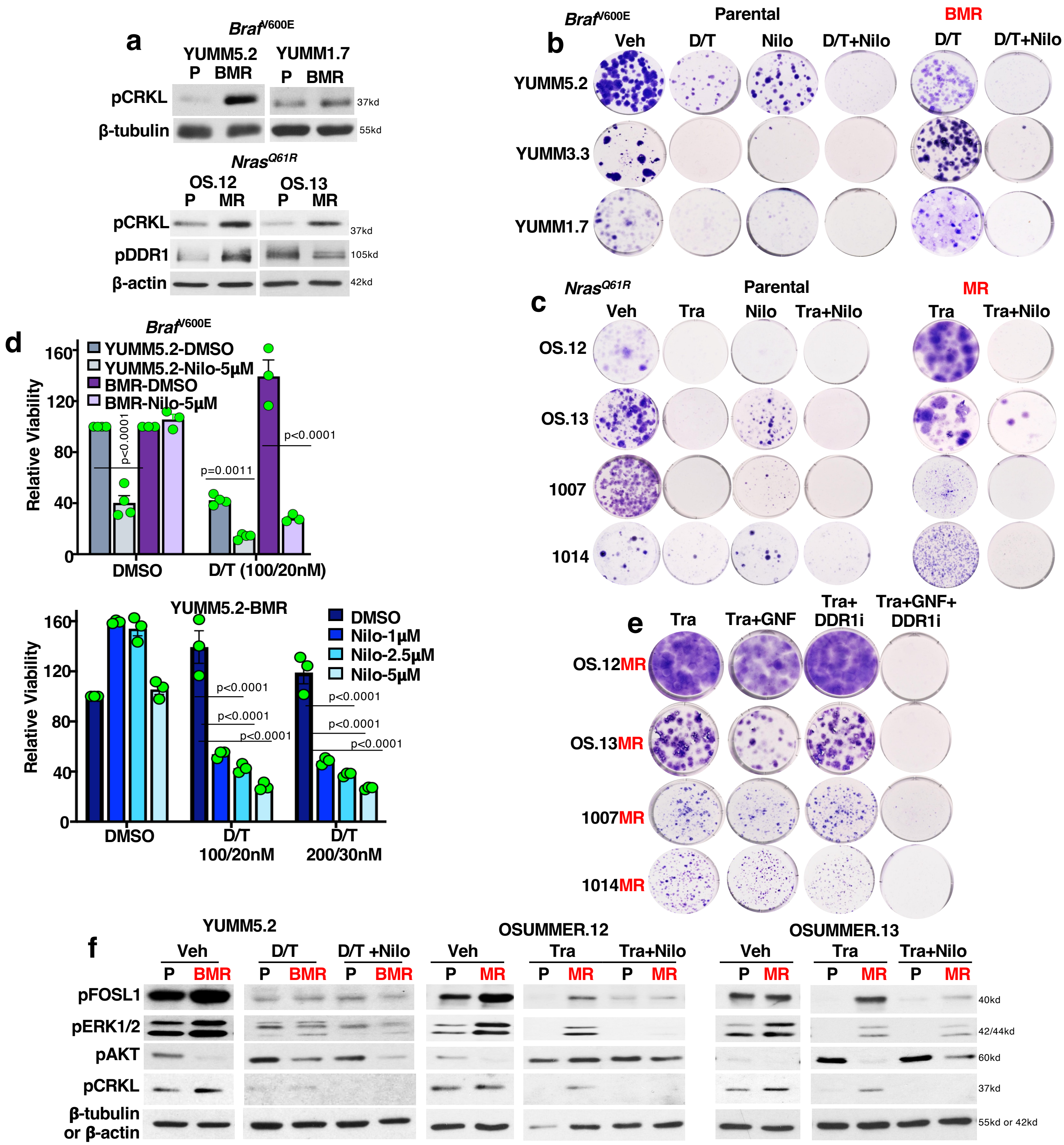

Supplementary Figure 2. Targeting ABL1/2 and/or DDR1 reverses MAPKi resistance. Mouse melanoma cells (parental; P) that acquired resistance to BRAFi/MEKi (dabrafenib/trametinib-D/T; BMR, *Braf*-mutant) or MEKi (trametinib-Tra; MR, *Nras*-mutant) were obtained by growing in increasing drug doses. **(a)** Lysates from parental/resistant pairs were blotted. Phosphorylation CRKL, is a reliable readout of ABL1+ABL2 activities. Blots are representative of 3 independent experiments (see Source File). **(b,c,e)** Clonogenic assays. Resistant cells were maintained in D/T (100-150/20-30nM) or Tra (20nM), and parental/resistant cells treated -/+ nilotinib (Nilo; 0.5µM for 1007, 2.5µM for the rest of the lines) for 7d, washed, incubated without drugs until colonies formed, and stained with crystal violet. For **(e)**, GNF-5 (12.5µM; allosteric ABL1/2 inhibitor) was included for the length of the assay. DDR1i=DDR-IN-1=2.5µM. Additional biological replicates (n=2-4) are in the Source File. **(d)** Cell Viability (CellTiter Glo-72h; mean±SEM for 3-4 biological replicates performed in triplicate) assays. p-values are from two-way ANOVAs with Tukey multiple comparisons tests. **(f)** Resistant lines off D/T or Tra for 2d, were treated (24h), and lysates blotted. Blots are representative of n=2 (see Source File). Veh=vehicle

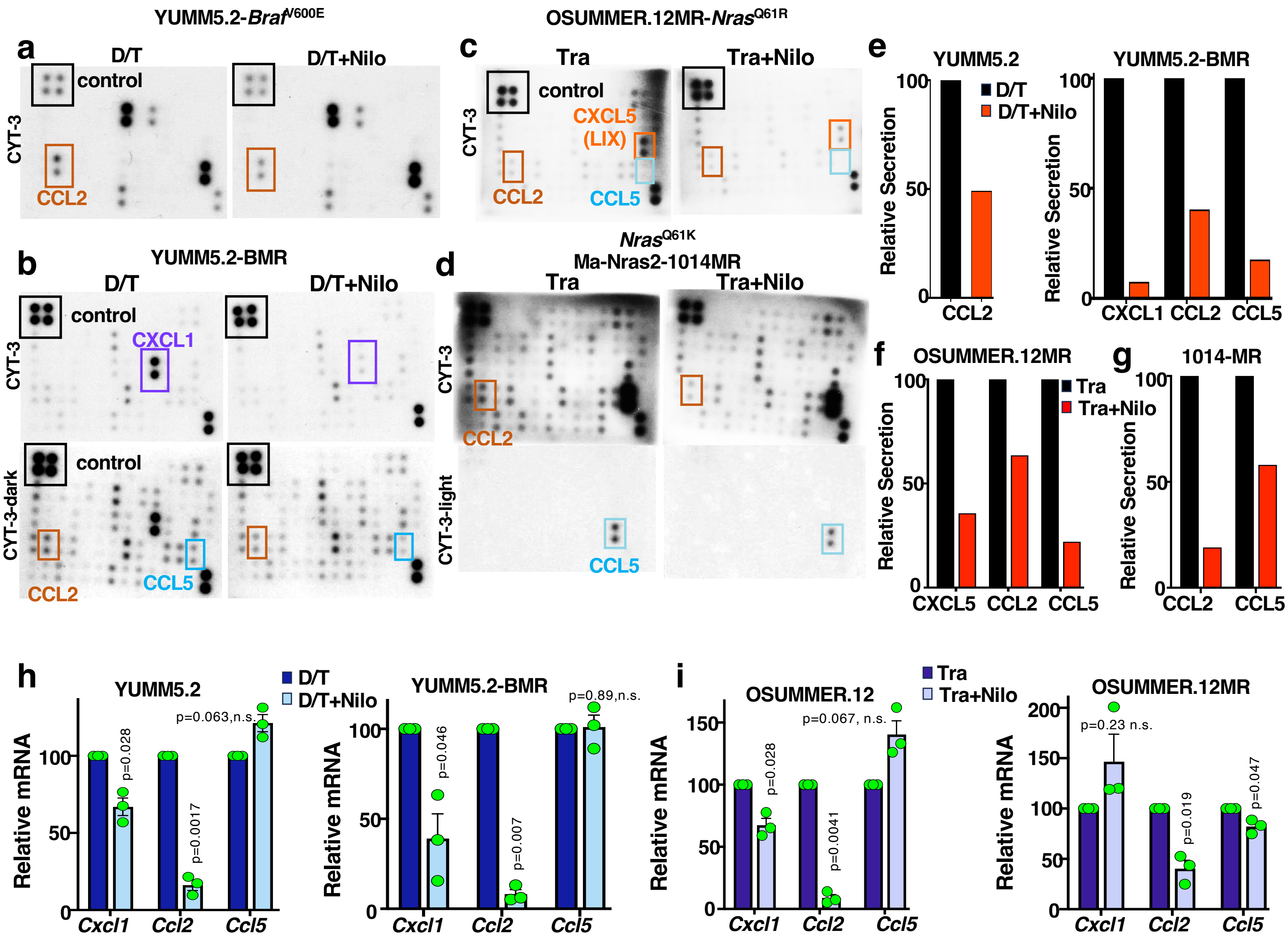

Supplementary Figure 3. Nilotinib reduces cytokine secretion and mRNA expression in mouse melanoma cells. (a-d) Mouse cytokine arrays were incubated with conditioned media from serum-starved, nilotinib (or vehicle)-treated (12-16h) mouse melanoma cells. *Braf*-mutant, BRAFi/MEKi resistant lines (BMR) and *Nras*-mutant MEKi-resistant lines (MR) were maintained in dabrafenib/trametinib-D/T (*Braf*-mutant) or trametinib (*Nras*-mutant) prior to and during serum-starvation. **(a-d)** Comparison of resistant lines -/+ nilotinib treatment (nilotinib added during serum-starvation). **(e-g)** ImageJ64 quantitation of mouse cytokine arrays from **a-d**. **(h,i)** Cytokine mRNA expression assessed by RT-qPCR. Values are relative to RPS13 and normalized to vehicle-treated. Mean±SEM for three independent experiments (biological replicates), performed in triplicate. p-values are 2-tailed values from single-sample t-tests. Each set of tests is relative to its own control, which was set to 100%. BMR=BRAFi/MEKi-resistant line; MR=MEKi resistant line. Nilo=nilotinib. n.s.=non-significant

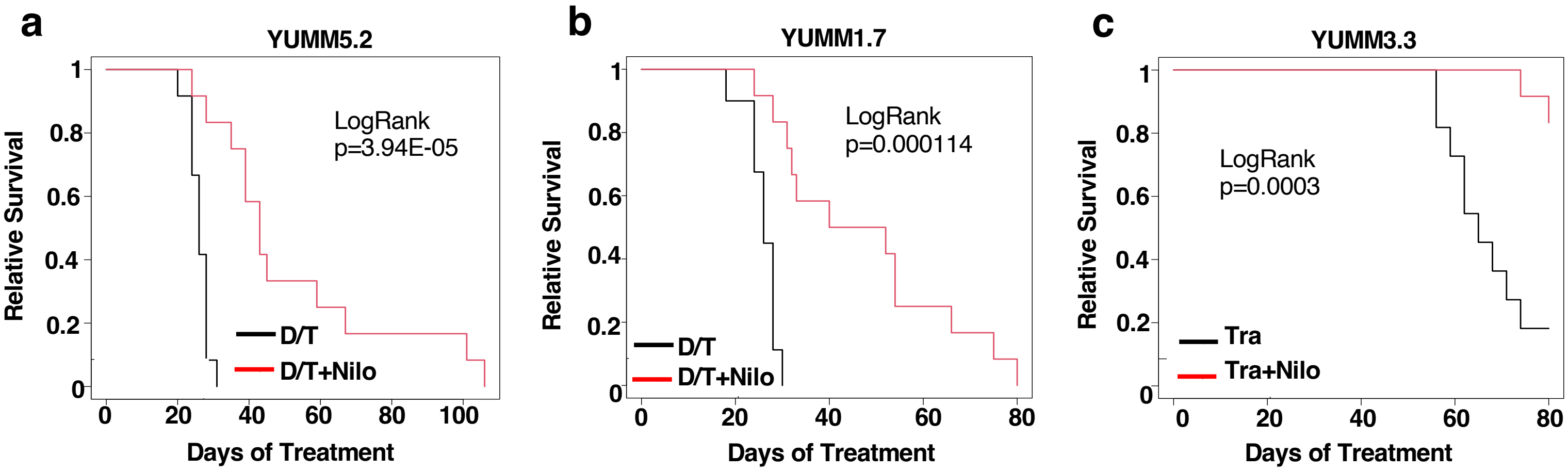

Supplementary Figure 4. Nilotinib increases survival for mice harboring syngeneic melanomas. Supporting data for **Fig. 3.** C57BL/6 mice harboring syngeneic *Braf*-mutant melanomas (100-200mm^3) were treated with dabrafenib/trametinib (BRAFi/MEKi, D/T; 25mg/kg/d, 0.15mg/kg/d) or trametinib (Tra, 0.85mg/kg/d) in the absence or presence of nilotinib (Nilo; 84mg/kg/d). Kaplan-Meier survival analysis using tumor doubling as the threshold. **a,c**: n=12/group. **b:** D/T, n=7; D/T+Nilo, n=9. p-values are two-tailed Log Rank Tests. Male mice were used for YUMM5.2 and YUMM1.7 and female for YUMM3.3 in order to keep sex consistent with the cell line of origin.

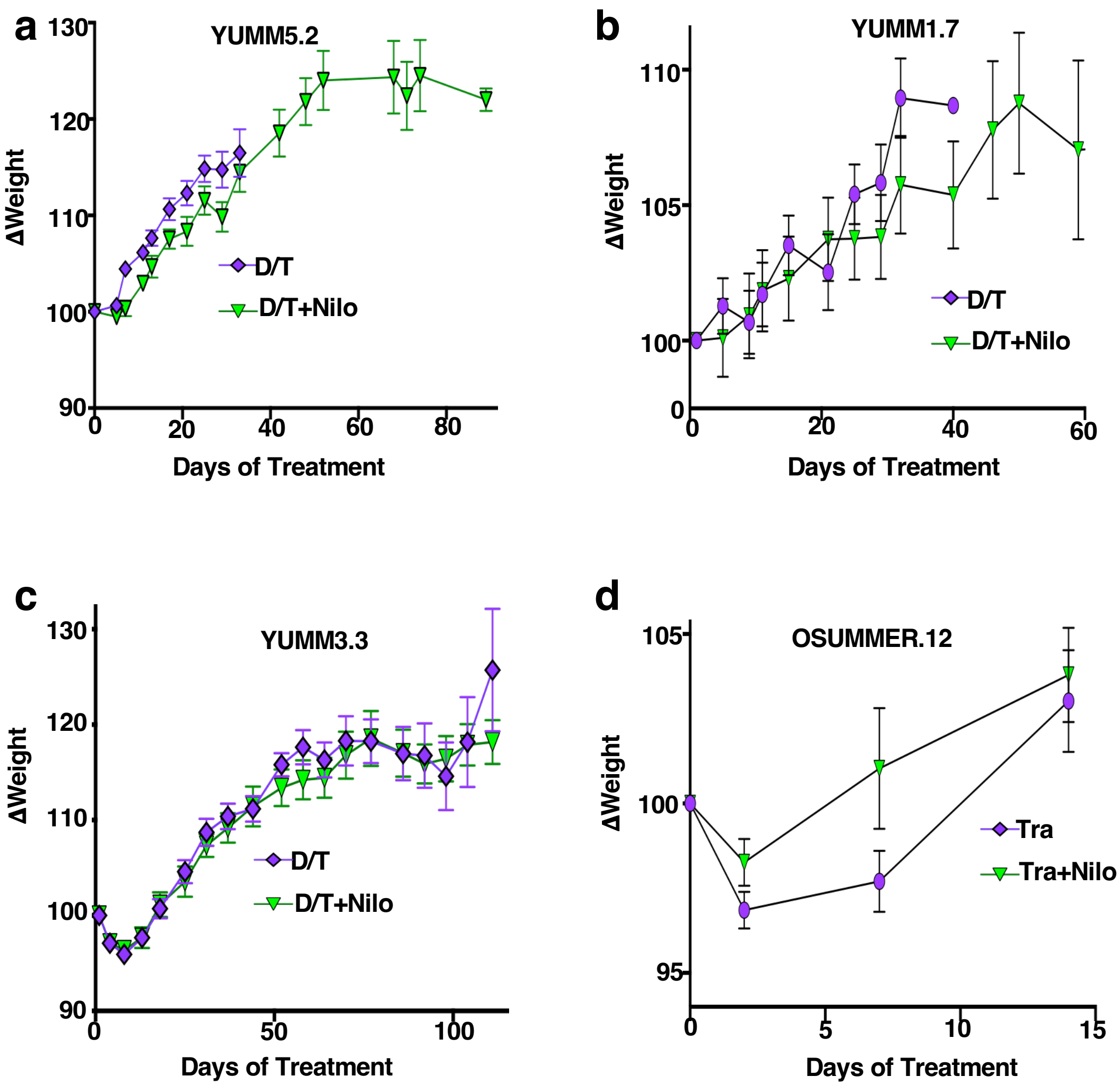

Supplementary Figure 5. Nilotinib treatment does not cause a reduction in the weight of mice harboring syngeneic melanomas. Supporting data for Fig. 3. Animal weights were measured over time and expressed as a percentage of baseline for syngeneic models in Fig. 3. Graphs are mean±SEM for all mice in the experiment. **a,c:** c=12/group. **b:** D/T, n=7; D/T+Nilo, n=9; **d:** n=5/group. D=dabrafenib, T=trametinib, Nilo=nilotinib. YUMM5.2, 1.7, 3.3 harbor *Braf*^{V600E}, while OSUMMER.12 contains an *Nras* mutation (Q61R). Male mice were used for YUMM5.2 and YUMM1.7 and female for YUMM3.3 and OSUMMER.12 in order to keep sex consistent with the cell line of origin.

a

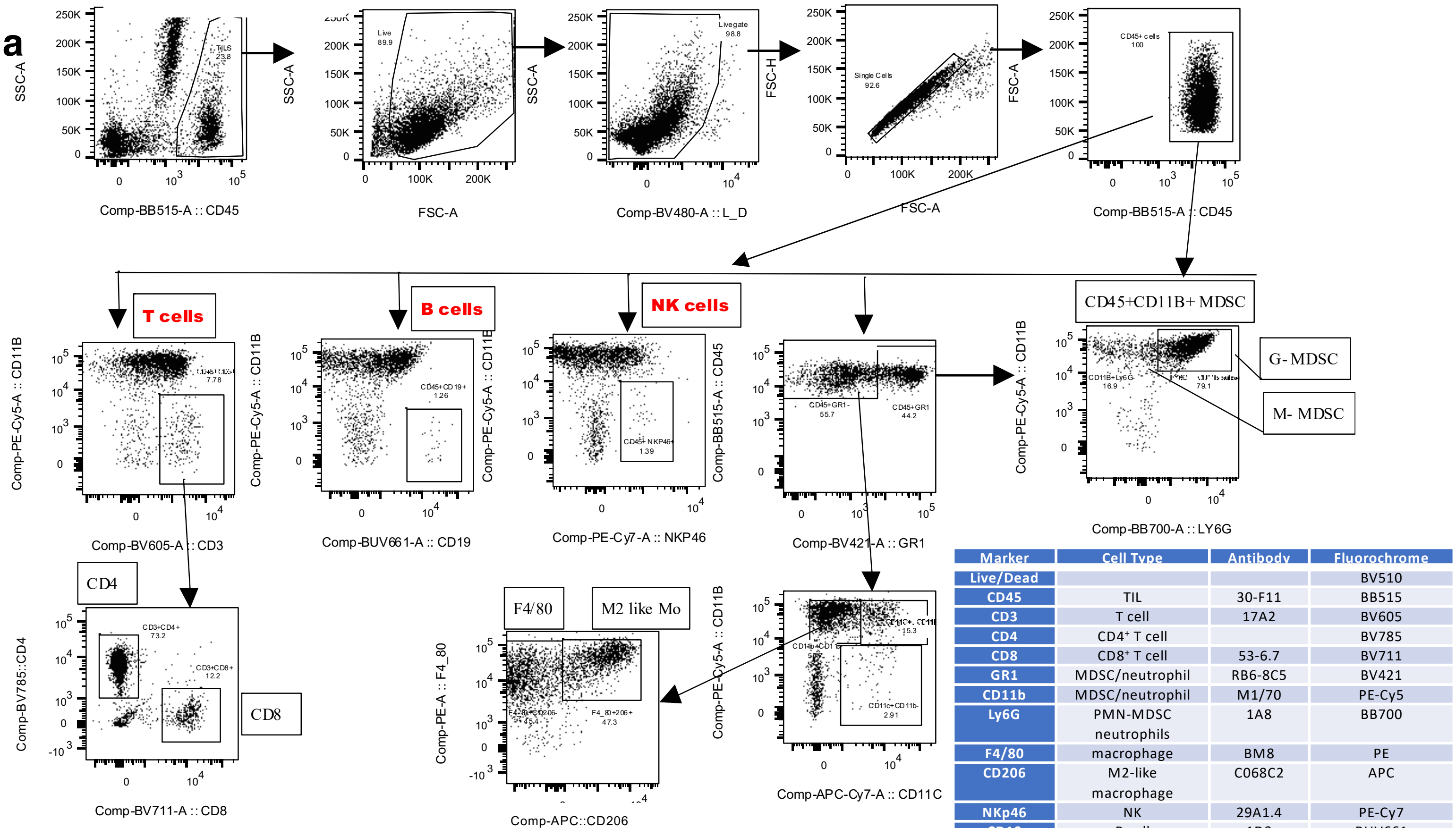

Marker	Cell Type	Antibody	Fluorochrome
Live/Dead			BV510
CD45	TIL	30-F11	BB515
CD3	T cell	17A2	BV605
CD4	CD4⁺ T cell		BV785
CD8	CD8⁺ T cell	53-6.7	BV711
GR1	MDSC/neutrophil	RB6-8C5	BV421
CD11b	MDSC/neutrophil	M1/70	PE-Cy5
Ly6G	PMN-MDSC neutrophils	1A8	BB700
F4/80	macrophage	BM8	PE
CD206	M2-like macrophage	C068C2	APC
NKp46	NK	29A1.4	PE-Cy7
CD19	B cell	1D3	BUV661

b

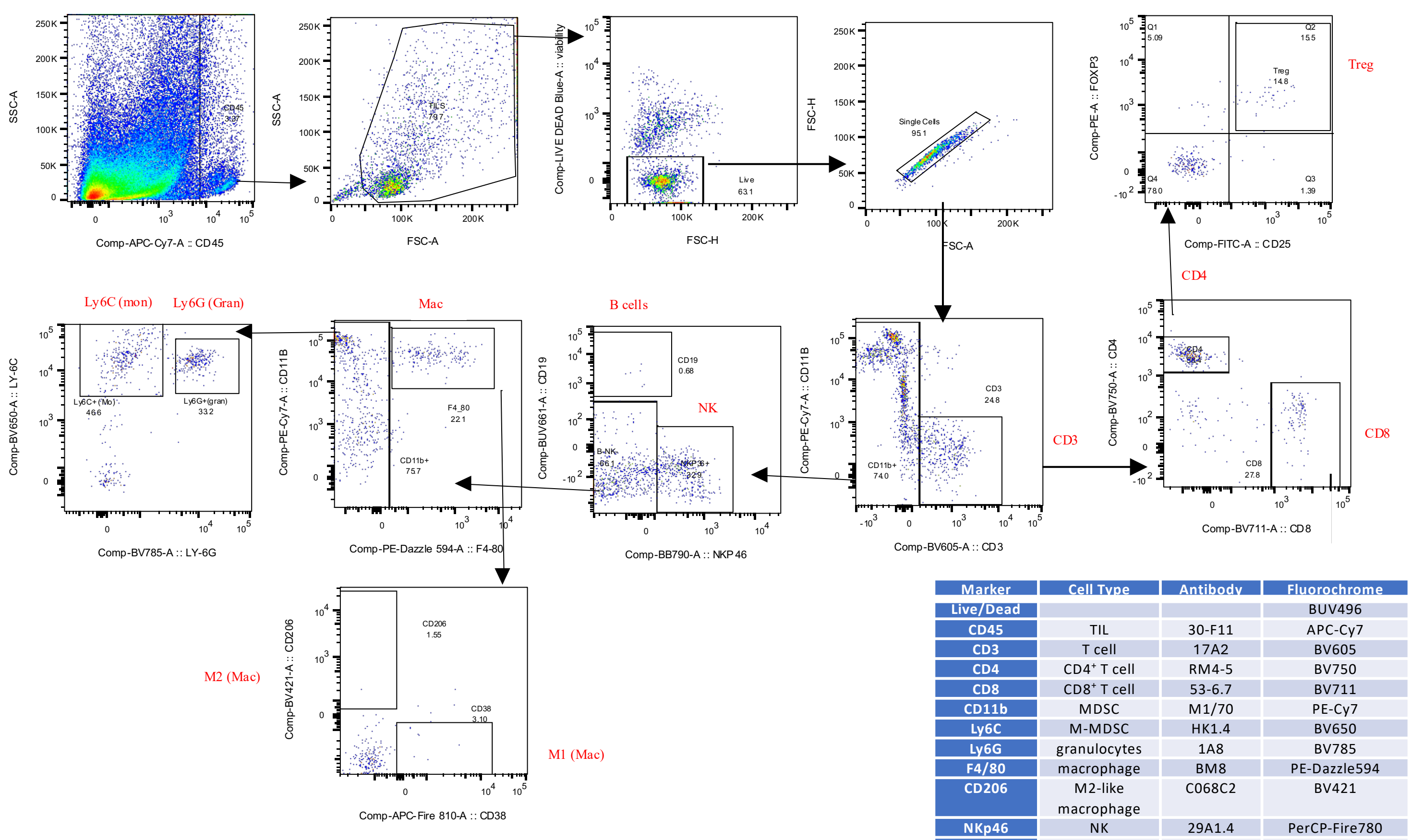

Marker	Cell Type	Antibody	Fluorochrome
Live/Dead			BUV496
CD45	TIL	30-F11	APC-Cy7
CD3	T cell	17A2	BV605
CD4	CD4⁺ T cell	RM4-5	BV750
CD8	CD8⁺ T cell	53-6.7	BV711
CD11b	MDSC	M1/70	PE-Cy7
Ly6C	M-MDSC	HK1.4	BV650
Ly6G	granulocytes	1A8	BV785
F4/80	macrophage	BM8	PE-Dazzle594
CD206	M2-like macrophage	C068C2	BV421
NKp46	NK	29A1.4	PerCP-Fire780
CD19	B cell	6D5	BUV661

Supplementary Figure 6. Flow Cytometry Gating Strategies. Supporting data for Figs. 4, 6. **(a)** Antibody staining and gating strategy for Symphony analysis of YUMM5.2 tumors (Figs. 4a,b,d,h; 6g,i,j, and Supplementary Figs. 7a,b,d,g; 12a,c,d,f,g,i,j). **(b)** Staining and gating strategy for Cytek Aurora analysis for OSUMMER.12 tumors (Figs. 4c, 6h,k and Supplementary Fig. 7c, 12b,e,h,k). Tables indicate the antibodies and fluorochromes used for staining.

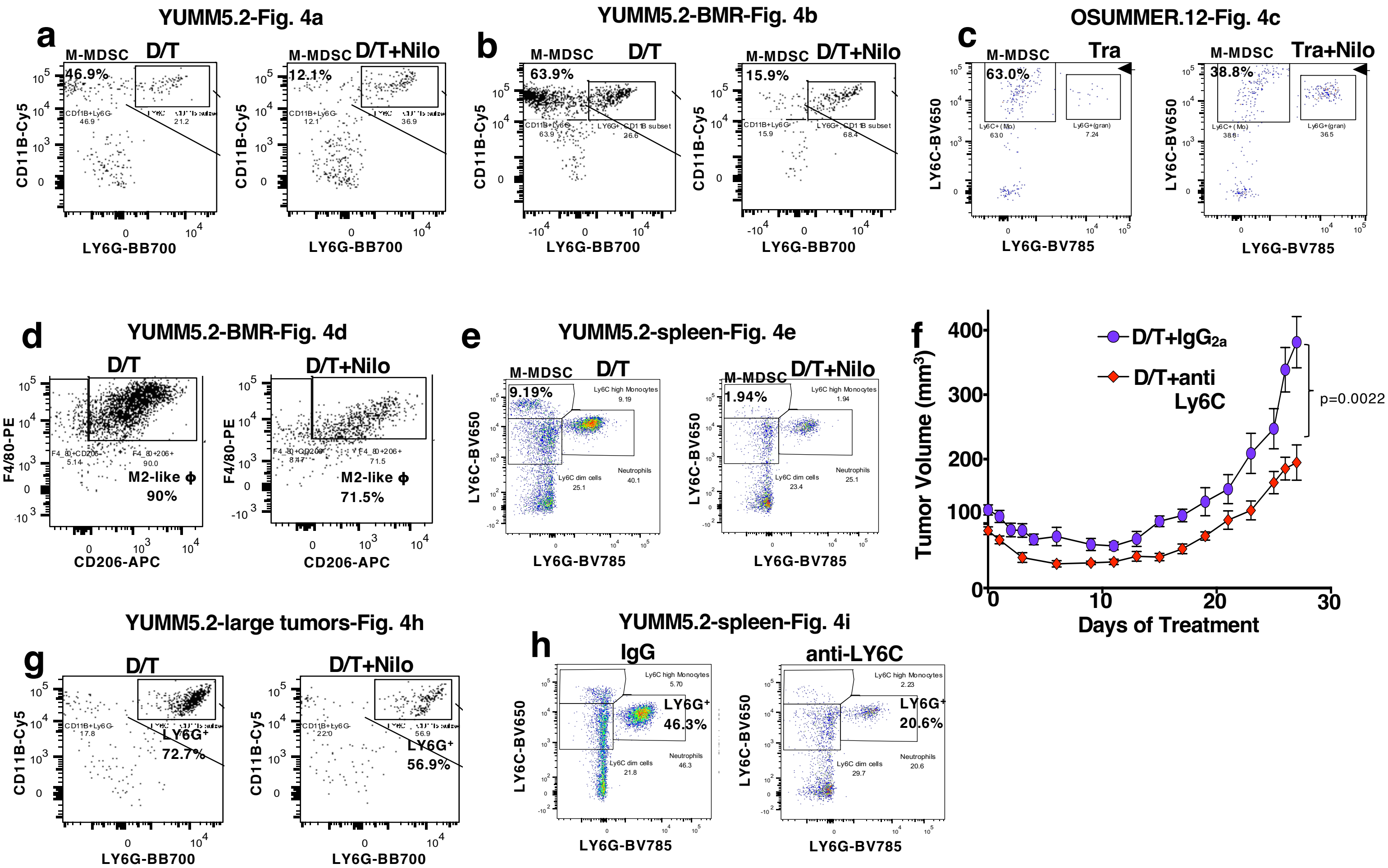

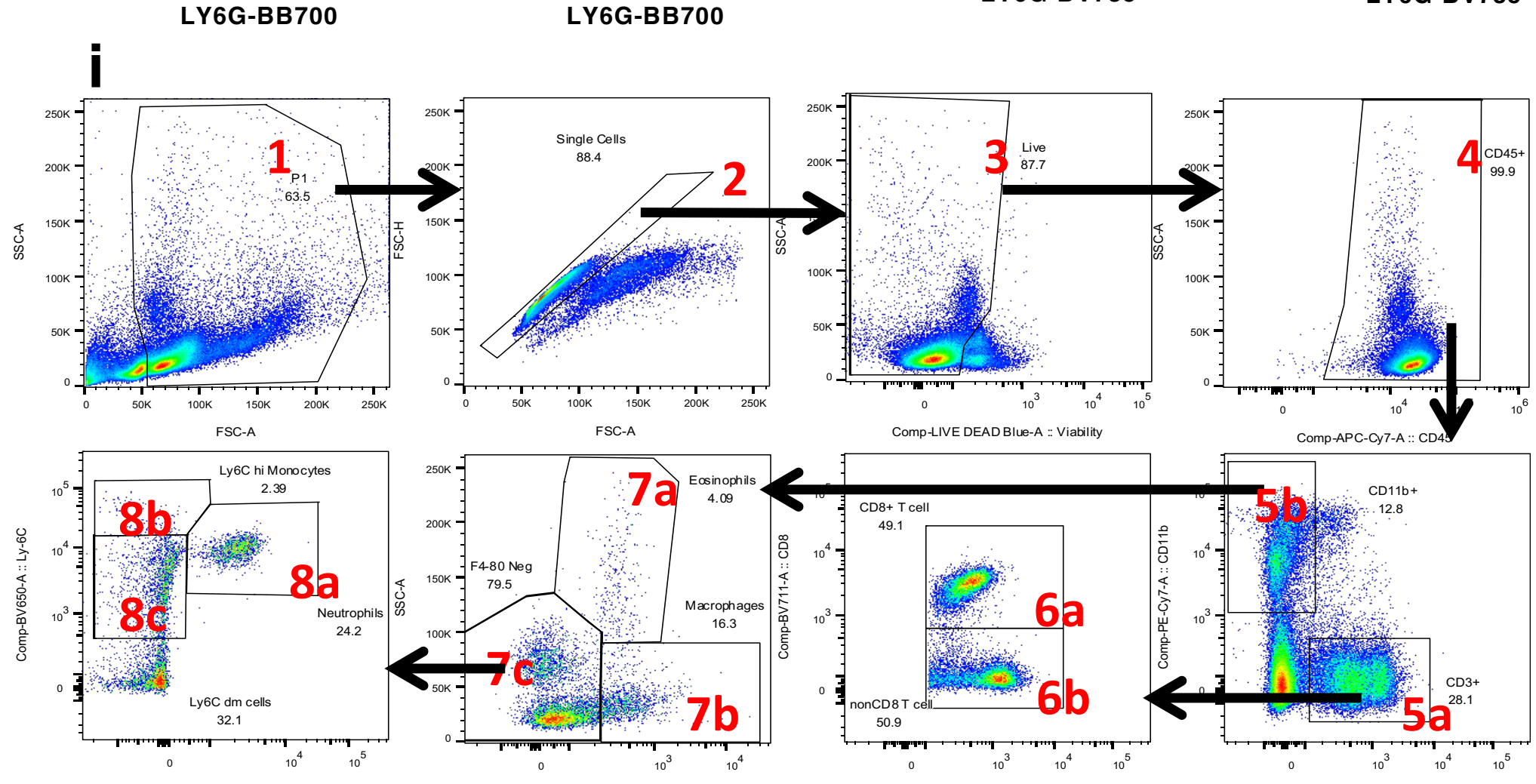

Marker	Cell Type	Antibody	Fluorochrome
Live/Dead			BUV496
CD45	TIL	30-F11	APC-Cy7
CD3	T cell	17A2	BV605
CD4	CD4+ T cell	RM4-5	BV750
CD8	CD8+ T cell	53-6.7	BV711
CD11b	MDSC	M1/70	PE-Cy7
Ly6C	M-MDSC	HK1.4	BV650
Ly6G	granulocytes	1A8	BV785
F4/80	macrophage	BM8	PE-Dazzle594

Supplementary Figure 7. ABL1/2 promote MDSC tumor infiltration. (a-e,h,i) Representative flow cytometry dot plots for data shown in Fig. 4a-e, h,i. **(f)** Supporting data for Fig. 4f,g. Tumor growth curves for Ly6C depletion study. Mean±SEM, n=7 mice/group. p-value is from a two-tailed, two sample t-test. **(i)** Antibody staining and gating strategy for flow cytometry (Cytek Aurora) analysis of spleen (used for Figs. 4e,i, 5g-i, 7h,i and Supplementary Figs. 7e,h, 8k,m, 9c-e).

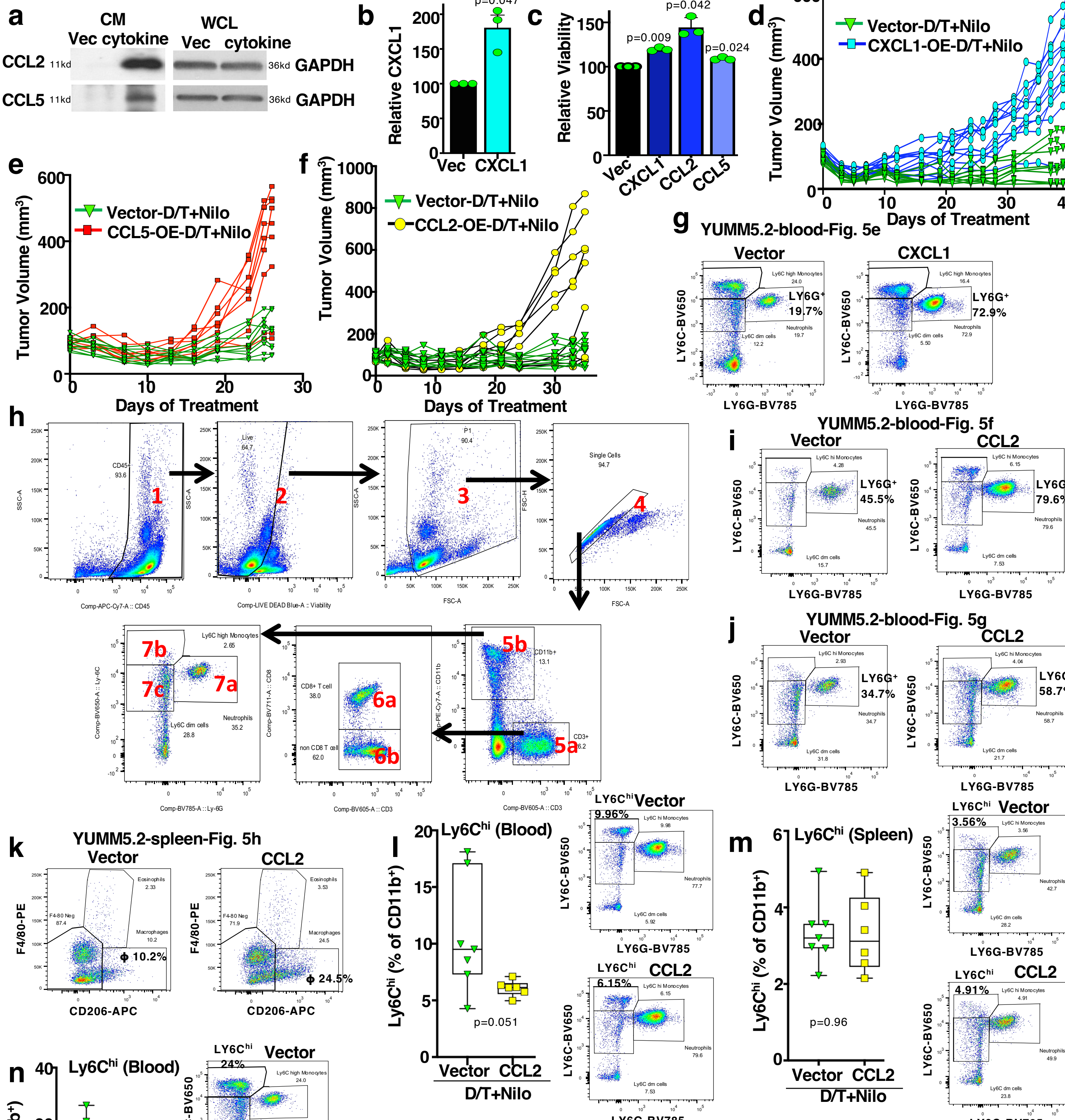

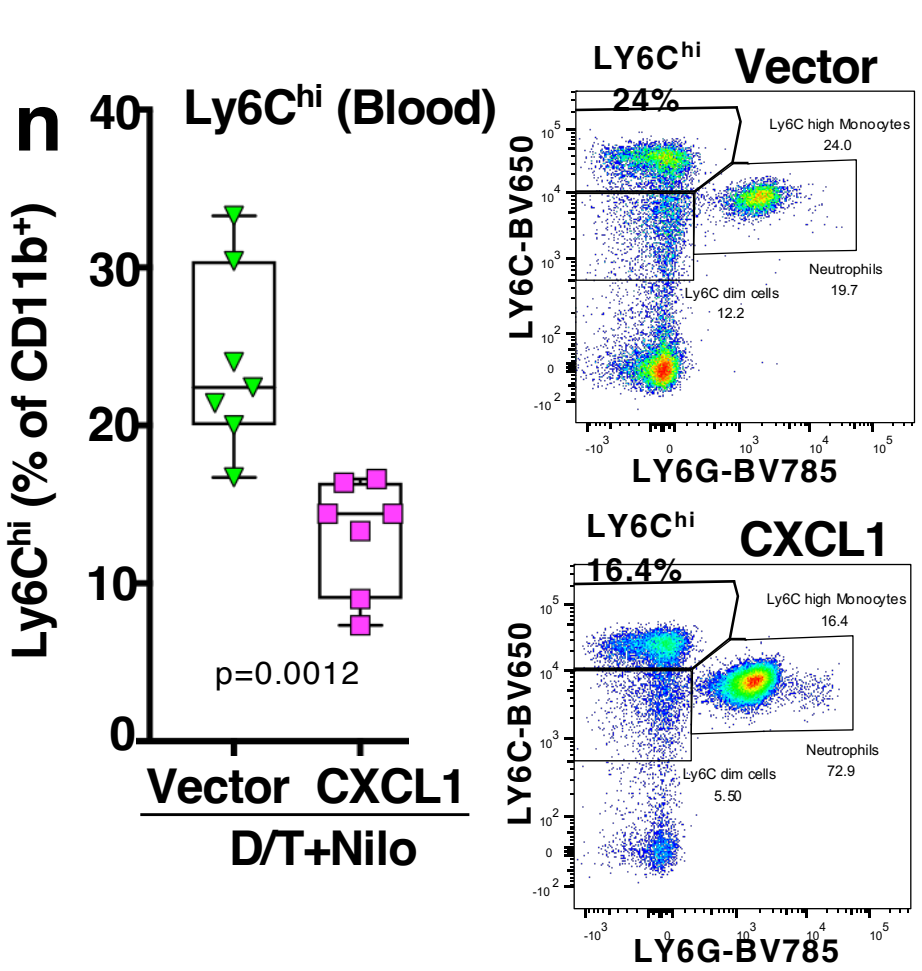

Supplementary Figure 8. Effects of cytokine overexpression. Supporting data for Fig. 5. Conditioned media (CM) from YUMM5.2 cells expressing (OE) vector or chemokines were subjected to western blot **(a)**, ELISA **(b)**, or CellTiter Glo (72h) survival assay **(c)**. Mean±SEM for 3 biological replicates (b,c). p-values are from 2-tailed single-sample t-tests using Holm's multiple comparison adjustment (c). For western blots, CM was loaded (left) and GAPDH (loading control for CM) was assessed in cell lysates (WCL). **(d-f)** Individual growth curves for data in Fig. 5a-c. **(g,i-k)** Representative dot plots for data in Fig. 5e-h. **(l-n)** Additional immune populations. Box-and-whisker plots indicate median, interquartile range (25th–75th percentiles), and whiskers extend to minimum-maximum values. Representative dot plots are to the right of graphs. **(h)** Gating strategy for flow cytometry of blood (Figs. 5e,f,7g; Supplementary Fig. 8g,i,j,l,n; 9a,b). Antibodies are in Supplementary Fig. 7i. p-values are two-tailed t-tests.

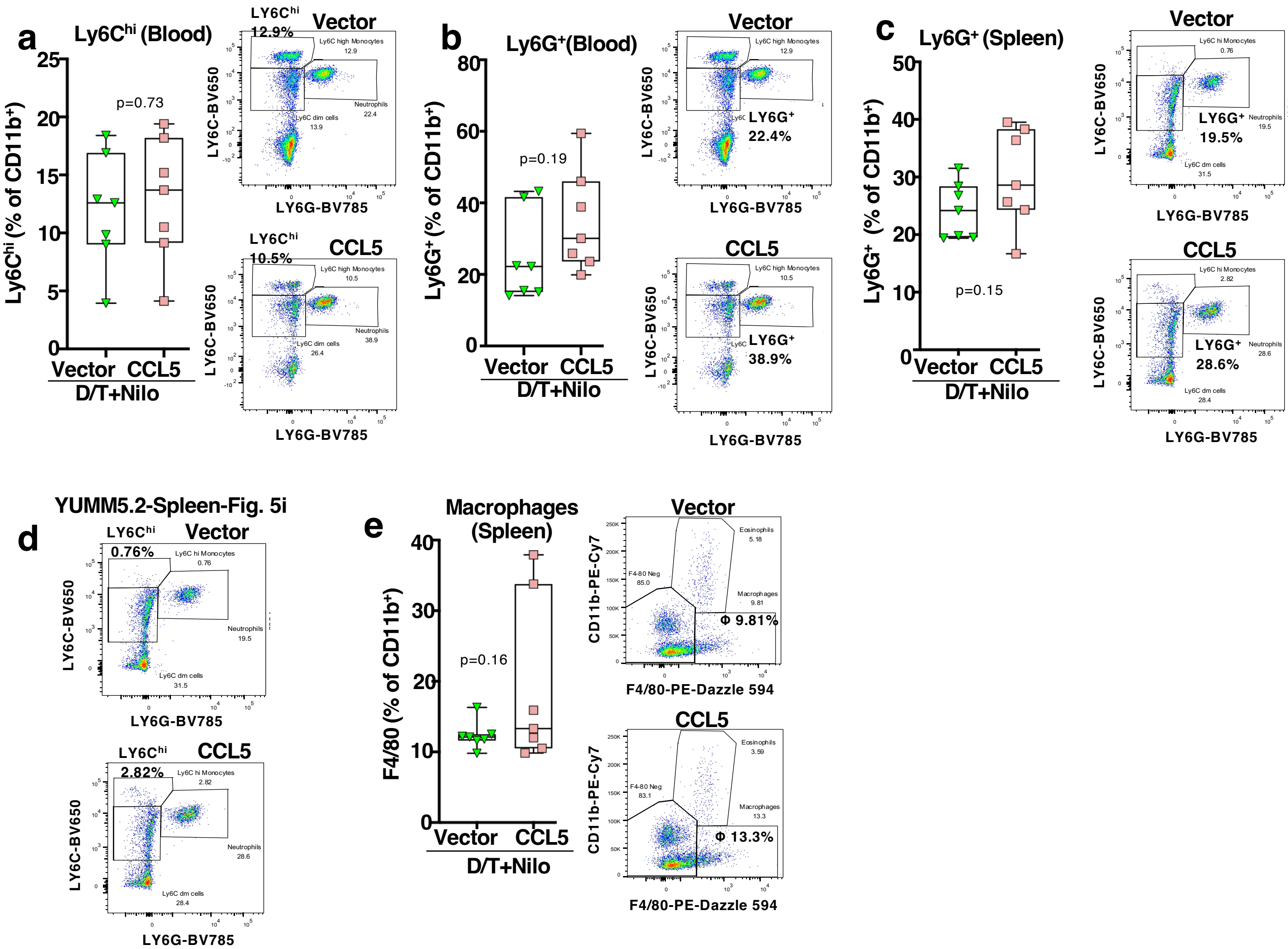

Supplementary Figure 9. CCL5 overexpression does not affect circulating and splenic LY6C^{hi} cells and splenic macrophages. Supporting data for Fig. 5. Antibody staining and subsequent flow cytometry of immune populations from peripheral blood or spleen from mice harboring YUMM5.2 tumors expressing vector or CCL5 and treated with D/T (dabrafenib/trametinib)+nilotinib. Immune cells were analyzed at the study endpoint. Gating strategies for blood **(a-b)** and spleen **(c-e)** are shown in Supplementary Fig. 8h and 7i, respectively. Box-and-whisker plots show the median (center line), interquartile range (box; 25th–75th percentiles), and whiskers extend to the minimum and maximum observed values. Representative dot plots are shown to the right of the graphs. p-values are from two-tailed, two sample t-tests.

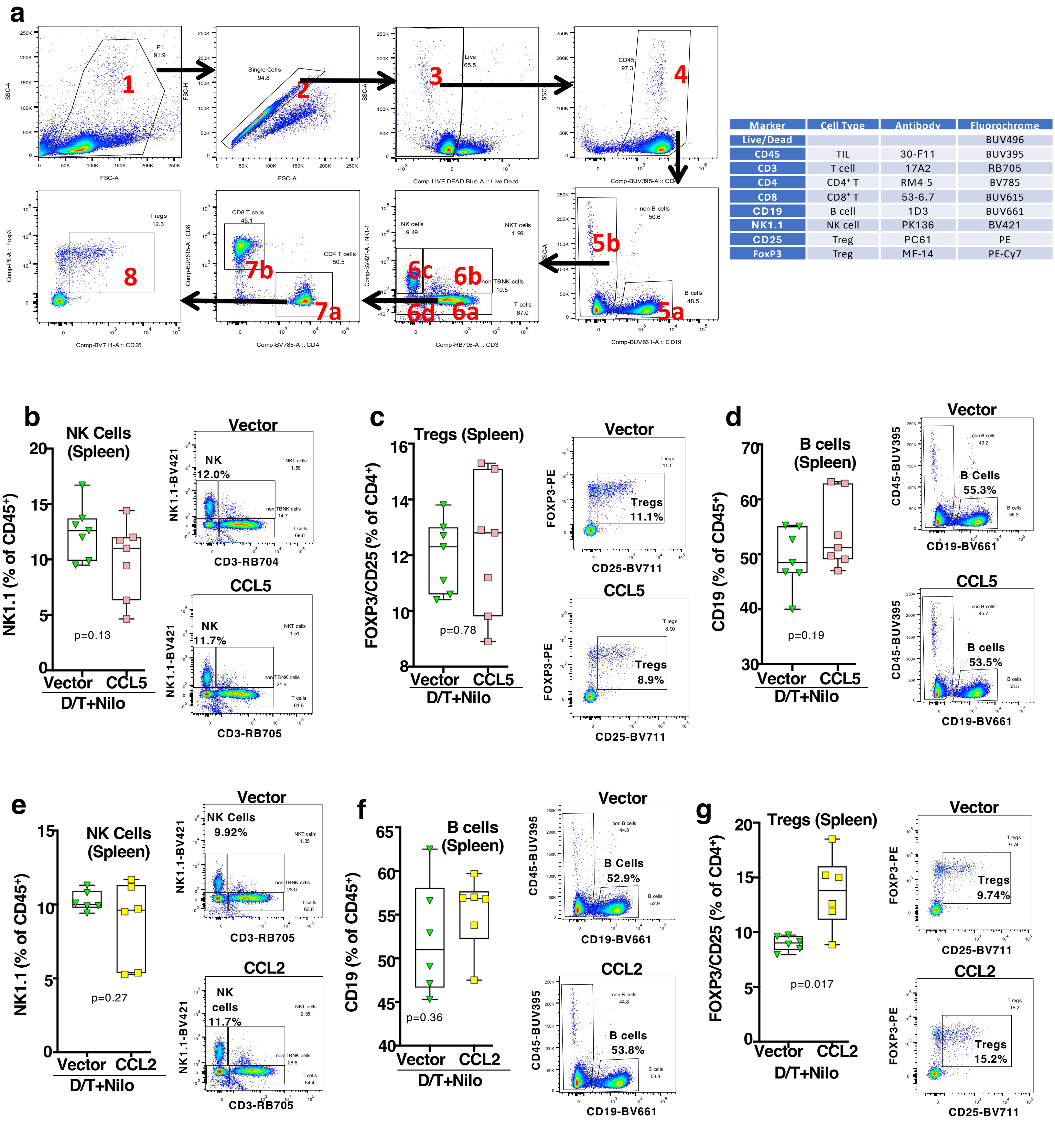

Marker	Cell Type	Antibody	Fluorochrome
Live/Dead			BUV496
CD45	TIL	30-F11	BUV395
CD3	T cell	17A2	RB705
CD4	CD4⁺ T	RM4-5	BV785
CD8	CD8⁺ T	53-6.7	BUV615
CD19	B cell	1D3	BUV661
NK1.1	NK cell	PK136	BV421
CD25	Treg	PC61	PE
FoxP3	Treg	MF-14	PE-Cy7

Supplementary Figure 10. Effects of cytokine overexpression on immune populations. (a) Antibody staining and gating strategies for flow cytometry (Cytek Aurora) analysis of spleens from mice harboring YUMM5.2 tumors expressing vector, CCL2, or CCL5 and treated D/T (dabrafenib/trametinib)+nilotinib (Nilo), assessing NK, B cell and Treg populations. **(b-g)** Box-and-whisker plots of immune cells from flow cytometry studies (a). Boxes show the median (center line), interquartile range (box; 25th–75th percentiles), and whiskers extend to the minimum and maximum observed values. Representative dot plots are to the right of the graphs. p-values are from two-tailed two sample t-tests.

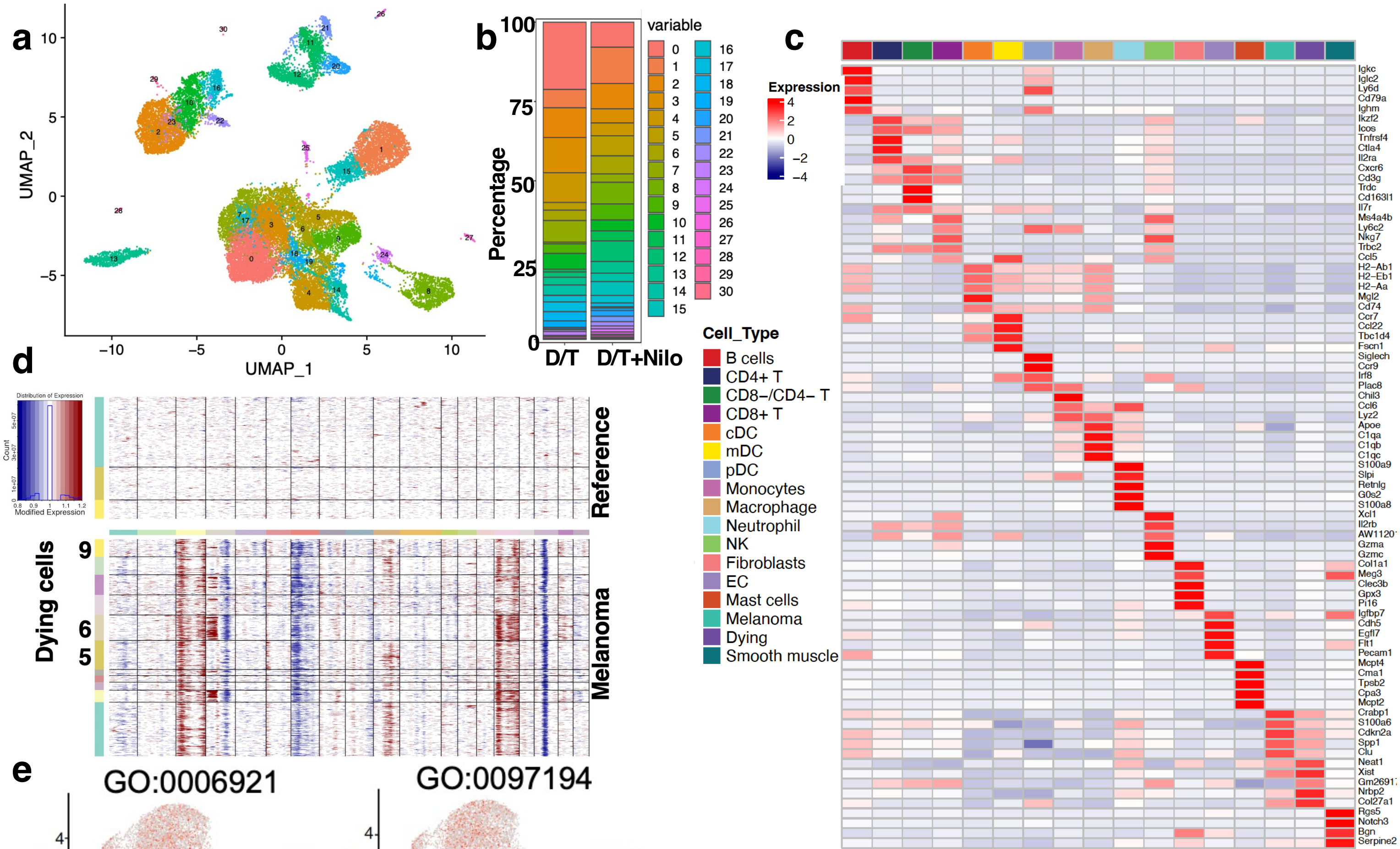

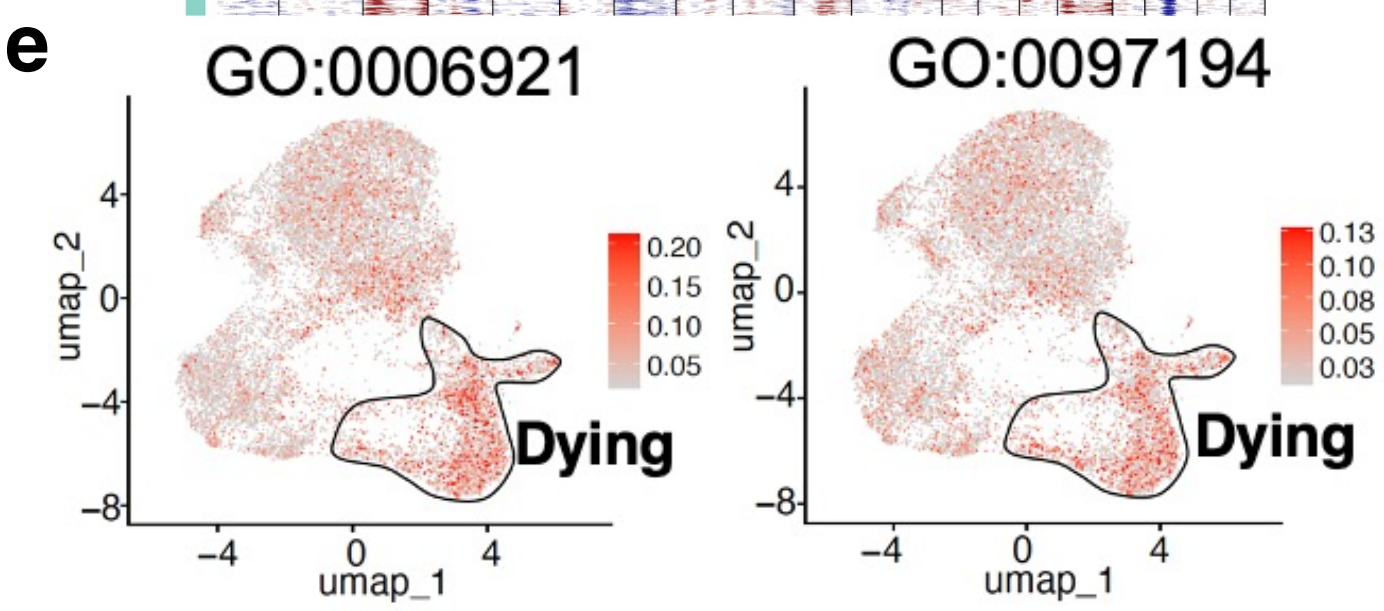

f

Fetit et al. Neutrophil Gene Signatures

"Healthy"	"Tumor"	"Metastasis"
MMP8	TXNIP	CDKN1A
IFITM6	RIPOR2	PPIA
S100A6	CXCR2	IFITM1
LYZ1	FKPB5	TAGIN2
LYZ2	CEBPD	ISG15
CTLA2A	STK17B	GNGT2
FPR2	SMAP2	CCL4
G0S2	CTSS	RPS271
	JAML	LER3
	IGHM	CCL3
	CD72	WFDC17
		THBS1
		PTMA

Wu et al. Neutrophil Gene Signatures

"HLA-DR+"	"VEGFR+"
PTPRCAP	CLCN7
IGKV3-20	MIF
HLA-DPB1	CCL3
HLA-DPA1	SLC3A2
AHNAK	PI3
HLA-DQA1	P4HA1
CD52	VEGFA
CD99	LGALS3
CRIP	RALGDS
HLA-DRB1	ENO1
NME2	C15orf48
CCL5	SC25A13
S100A10	CDKN1A
HLA-DQB1	CCRL2
IL32	CCL3L1
PPDPF	CXCL2
EZR	CD68
NR4A1	PLIN2
H1FX	LIMS1
COX7c	BHLHE40

Supplementary Figure 11. Immune populations identified by single-cell RNA sequencing (scRNAseq). Supporting data for Fig. 6. Mice harboring small YUMM5.2 tumors were treated with dabrafenib/trametinib (D/T) or D/T+nilotinib (Nilo), and tumors subjected to scRNAseq. **(a)** Unsupervised cluster analysis. **(b)** Relative distribution of the 31 clusters in the treatment groups. **(c)** Identification of cell populations using an integrated analysis. The top 5 genes that mark each population are in dark red. **(d)** Melanoma clusters (bottom panel) were identified based on chromosomal amplification and deletion patterns inferred from InferCNV using myeloid populations (upper panel) as the diploid reference. The dying malignant subset comprises clusters 5, 6, and 9 **(a)**. **(e)** Feature plots of module scores for apoptosis-related gene ontology (GO) pathways in melanoma cells. Dying melanoma cells have elevated cellular component disassembly (left) and execution phase of apoptosis (right) pathway activity. **(f)** Neutrophil gene signatures from Fetit et al. and Wu et al. used for analysis in Fig. 6f.

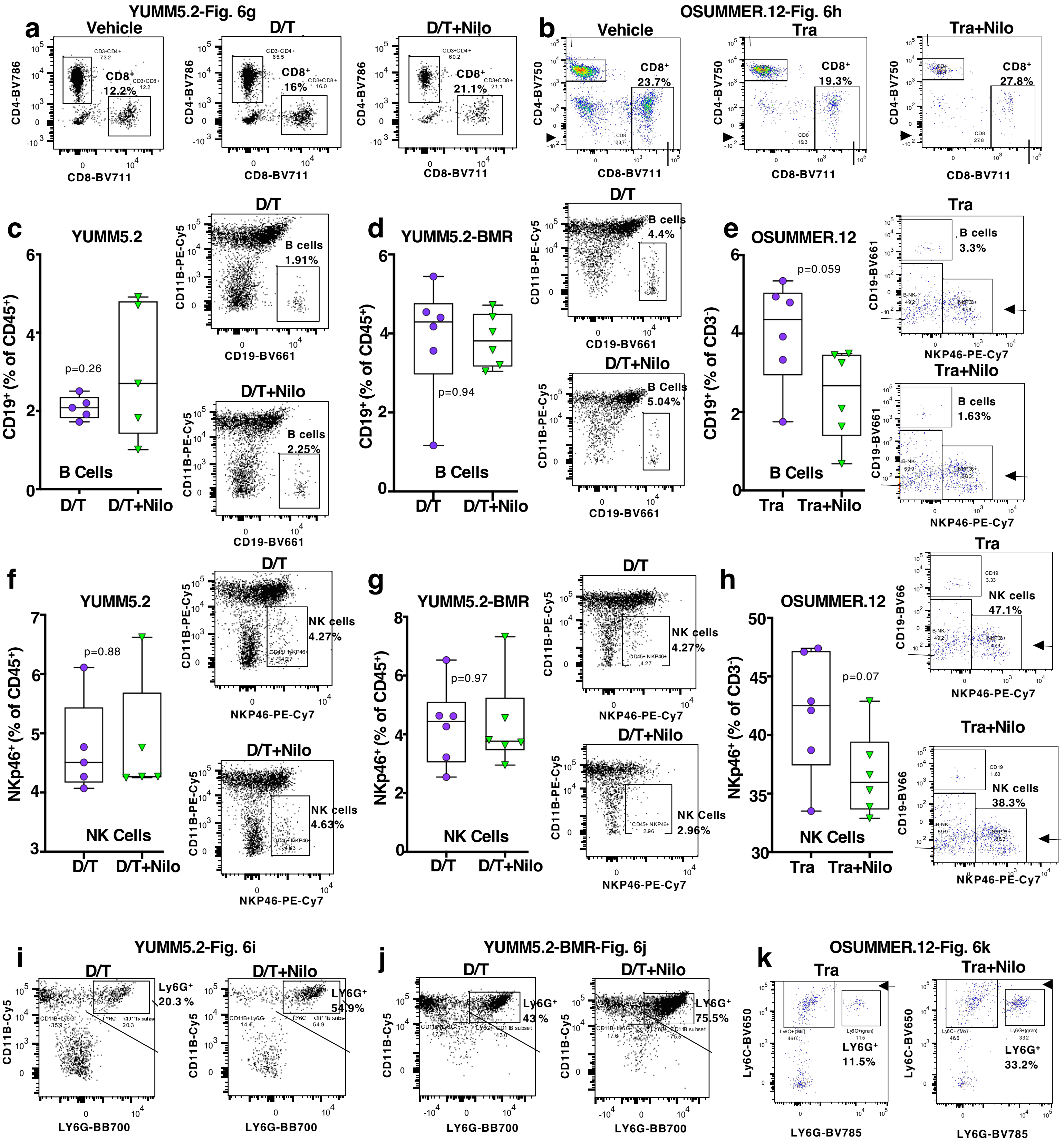

Supplementary Figure 12. Nilotinib increases CD8+ T cell infiltration but does not impact B or NK cells by flow cytometry. (a,b, i-k) Representative flow cytometry dot plots for data shown in Fig. 6g-k. **(c-e,f-h)** Tumors from mice harboring syngeneic melanomas (YUMM5.2-*Braf*^{V600E}; OSUMMER.12-*Nras*^{Q61R}) were subjected to antibody staining and FACS analysis. See Supplementary Fig. 6 for staining and gating strategies. Box-and-whisker and representative flow cytometry plots showing the effects of nilotinib treatment on B cells (c-e), and NK cells (f-h). Box-and-whisker plots show the median (center line), interquartile range (box; 25th–75th percentiles), and whiskers extend to the minimum and maximum observed values. p-values are from two-tailed, two-sample t-tests. BMR=BRAFi/MEKi-resistant syngeneic model. Nilo=nilotinib; D/T=dabrafenib/trametinib; T=trametinib.

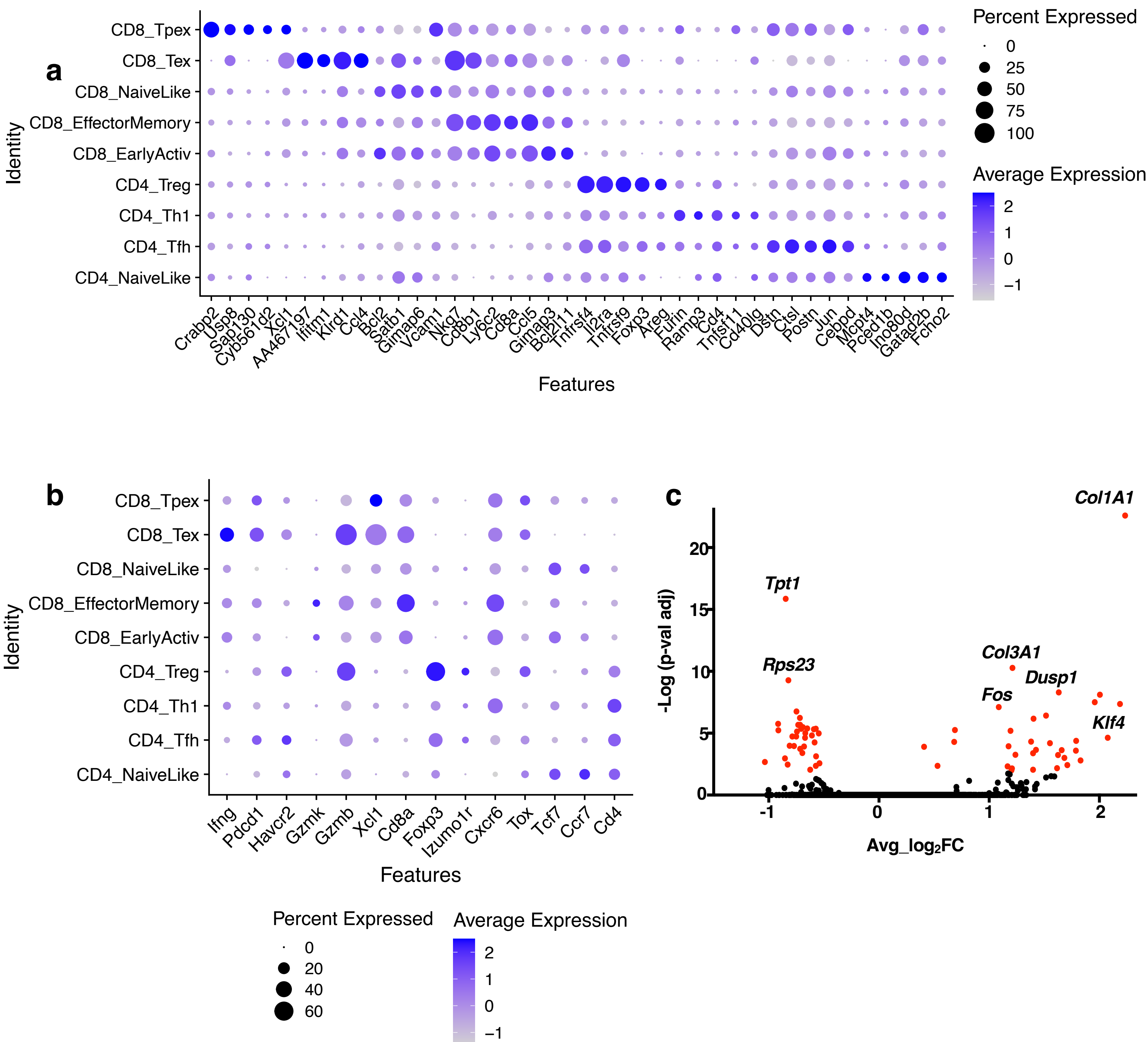

Supplementary Figure 13. T cell populations identified in mouse melanomas by scRNAseq. Supporting data for Fig. 7. **(a,b)** CD8⁺ and CD4⁺ clusters were subclassified using markers described in Andreatta et al. (2021). **(c)** Analysis of differentially expressed genes (D/T+nilotinib vs. D/T) in the CD8⁺ T cell population. See Supplementary Dataset 3, CD8 T cell Tab. Volcano plot. Red=significantly changed genes (adjusted p-value<0.05). D=dabrafenib, T=trametinib

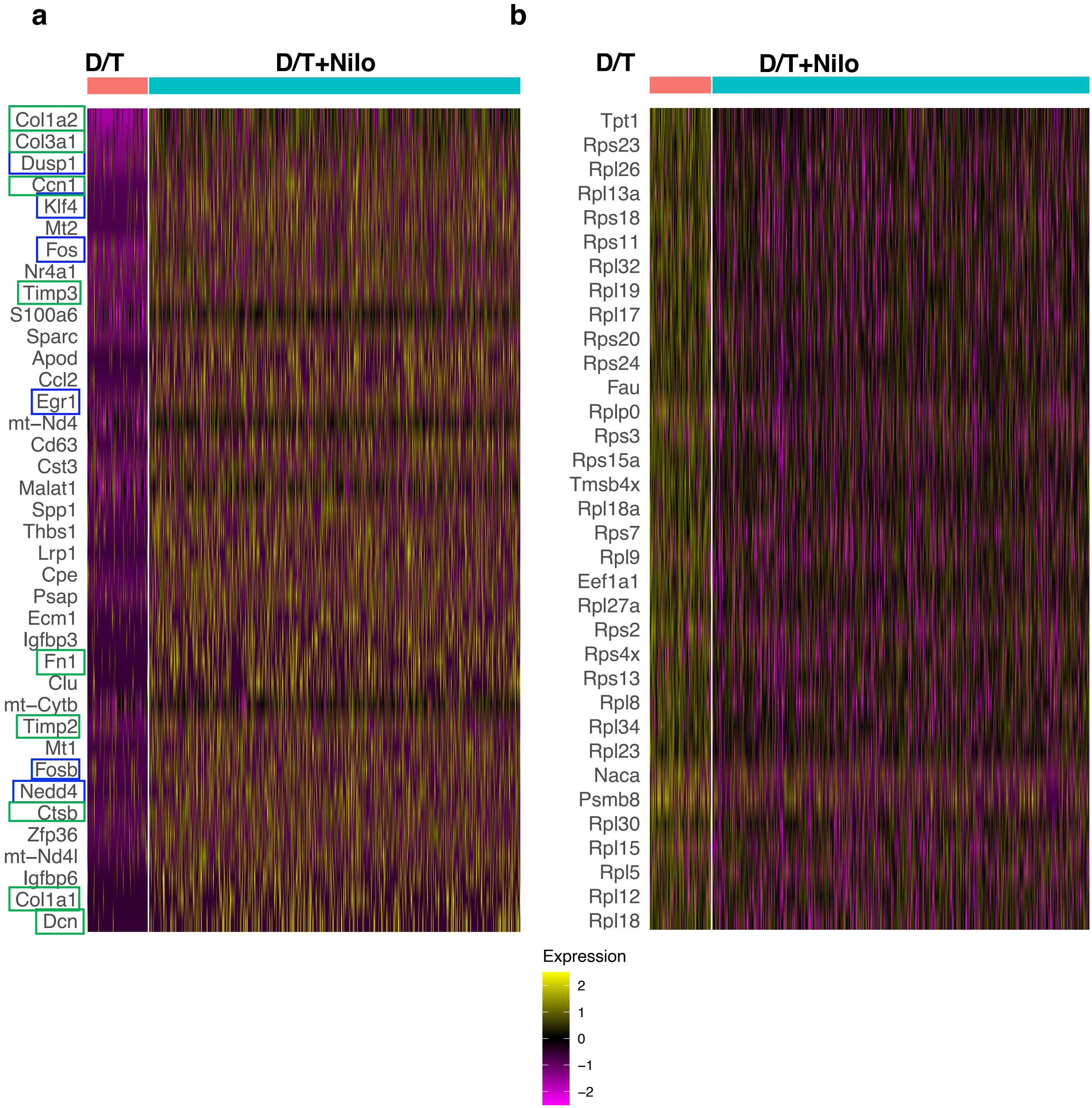

Supplementary Figure 14. Nilotinib treatment increases expression of genes involved in CD8⁺ T cell activation, proliferation, and migration. Heatmaps of the significantly upregulated **(a)** and downregulated **(b)** mRNAs (p-adj<0.05) arranged by p-value (lowest at the top) in CD8⁺ T cells from mice treated with D/T+nilotinib versus CD8⁺ T cells from mice treated with D/T alone. Blue boxes are proliferative genes, and green boxes are migratory genes. D/T=dabrafenib/trametinib (BRAFi/MEKi); Nilo=nilotinib. Raw data is in Supplementary Dataset 3, CD8⁺ T cell tab.

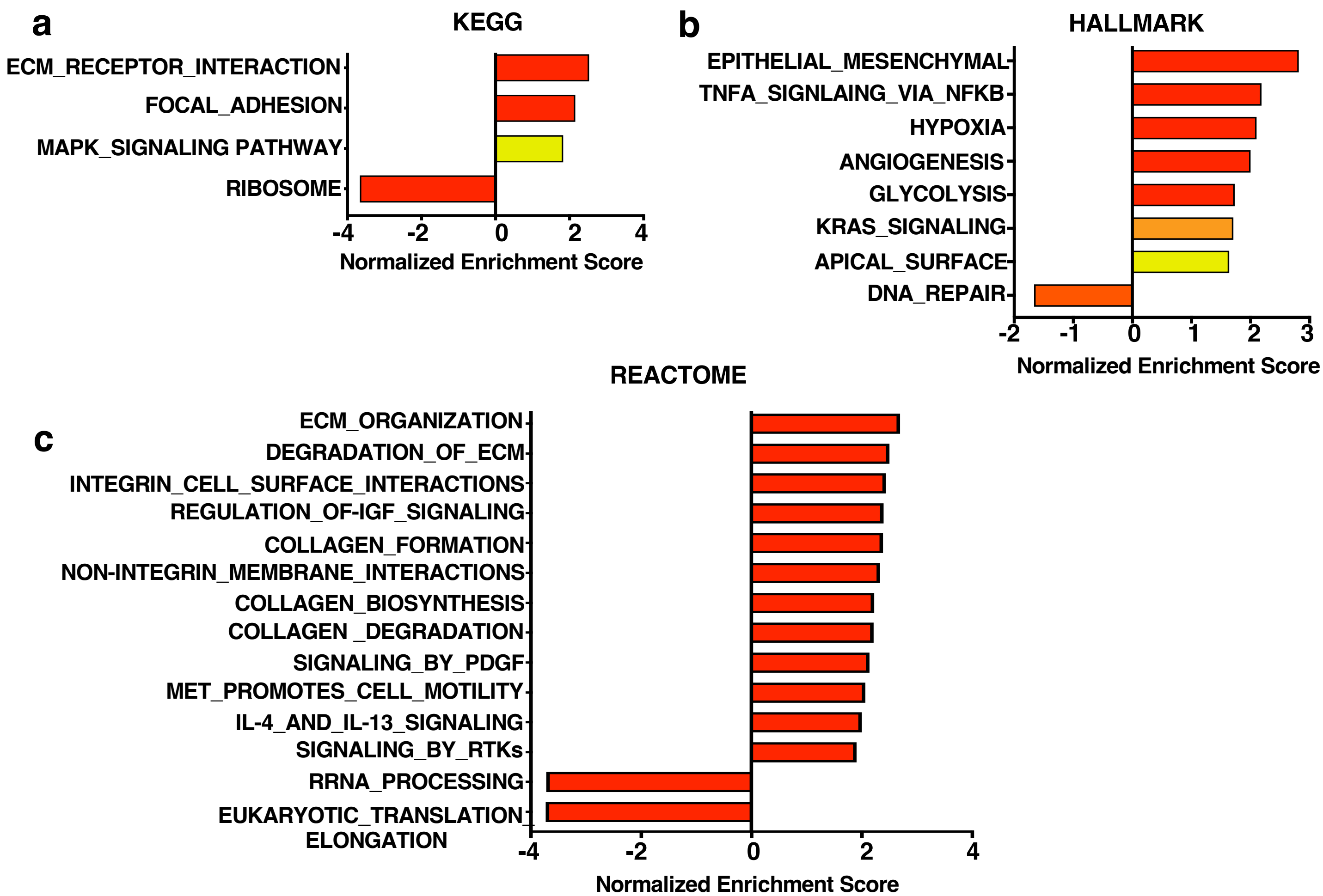

Supplementary Figure 15. Nilotinib treatment increases proliferative and migratory pathways in CD8⁺ T cells and represses translation. Geneset Enrichment Pathway (GSEA) analysis of differentially expressed pathways in CD8⁺ T cells comparing tumors from mice treated with D/T+nilotinib to those treated with D/T alone. **(a)** KEGG pathway analysis. **(b)** HALLMARK pathway analysis. **(c)** REACTOME pathway analysis. Graphed from data in Supplementary Dataset 6.

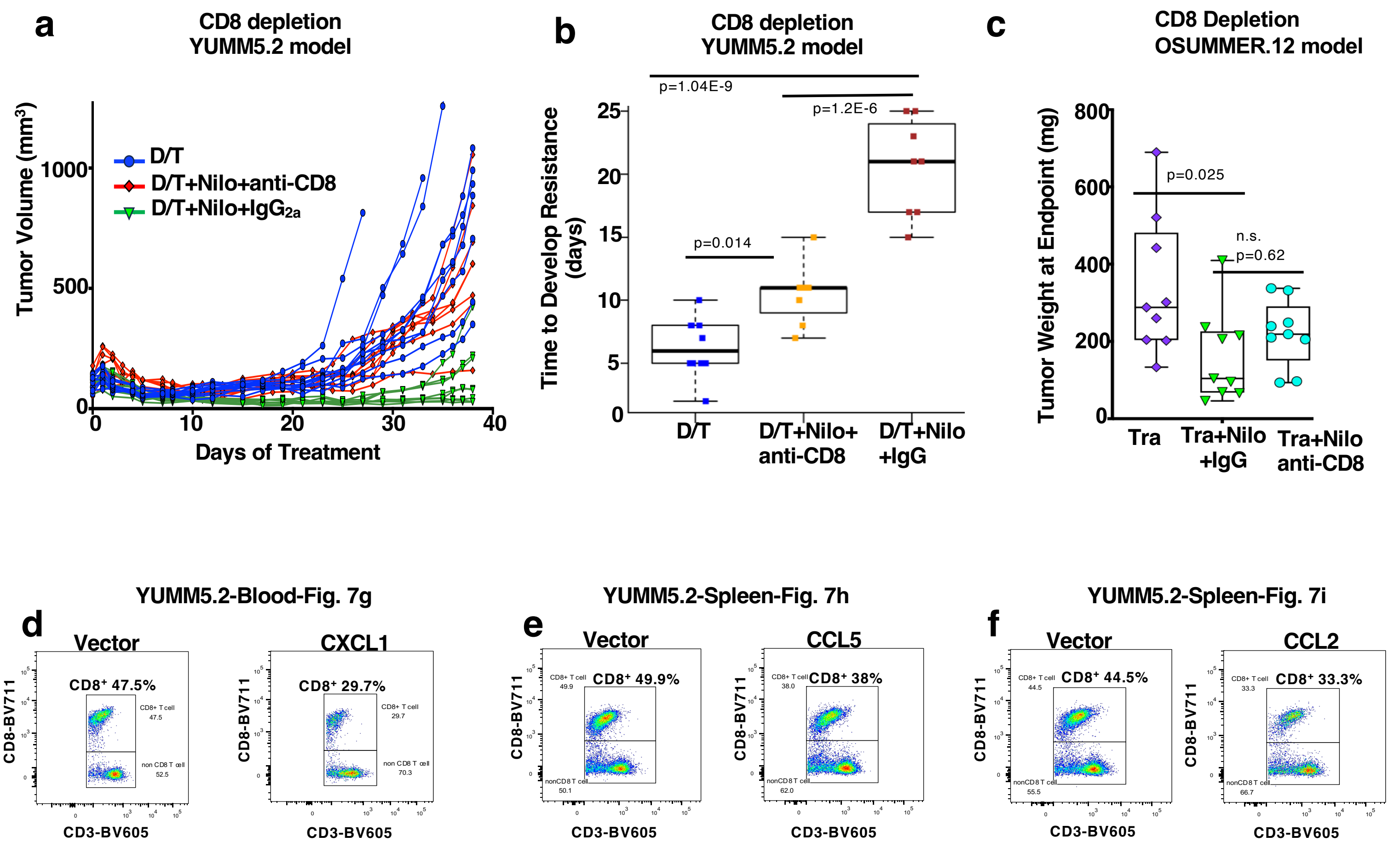

Supplementary Figure 16. CD8$^+$ T cells are required for nilotinib to prevent MAPKi resistance. Support for Fig. 7e,f. CD8$^+$ T cells were depleted (anti-CD8 or IgG$_{2a}$ control, beginning 3d prior to tumor injection, every 3d) from C57BL/6J mice harboring YUMM5.2 tumors and treated with D/T+Nilotinib. Mice treated with D/T served as a comparison group to be certain nilotinib+IgG was behaving as expected. **(a)** Individual mouse growth curves. **(b)** The time to develop resistance was estimated by recording the day at which tumors were at their smallest sizes (tumor growth accelerated after this day). Two-tailed, one-way ANOVA p=1.52e-09. Tukey posthoc multiple comparisons test p-values are shown. **(c)** C57BL/6 mice harboring OSUMMER.12 tumors were used for CD8 depletion studies as in **a,b** and Fig. 7e,f. Tumor weights at the study endpoints were plotted. p-values are from a one-way ANOVA and Tukey's multiple comparisons test. n.s.=non-significant. Box-and-whisker plots (b,c) show the median (center line), interquartile range (box; 25th–75th percentiles), and whiskers extend to the minimum and maximum observed values. **(d-f)** Representative flow cytometry dot plots for data shown in Fig. 7g-i. D/T=dabrafenib/trametinib; Tra=trametinib

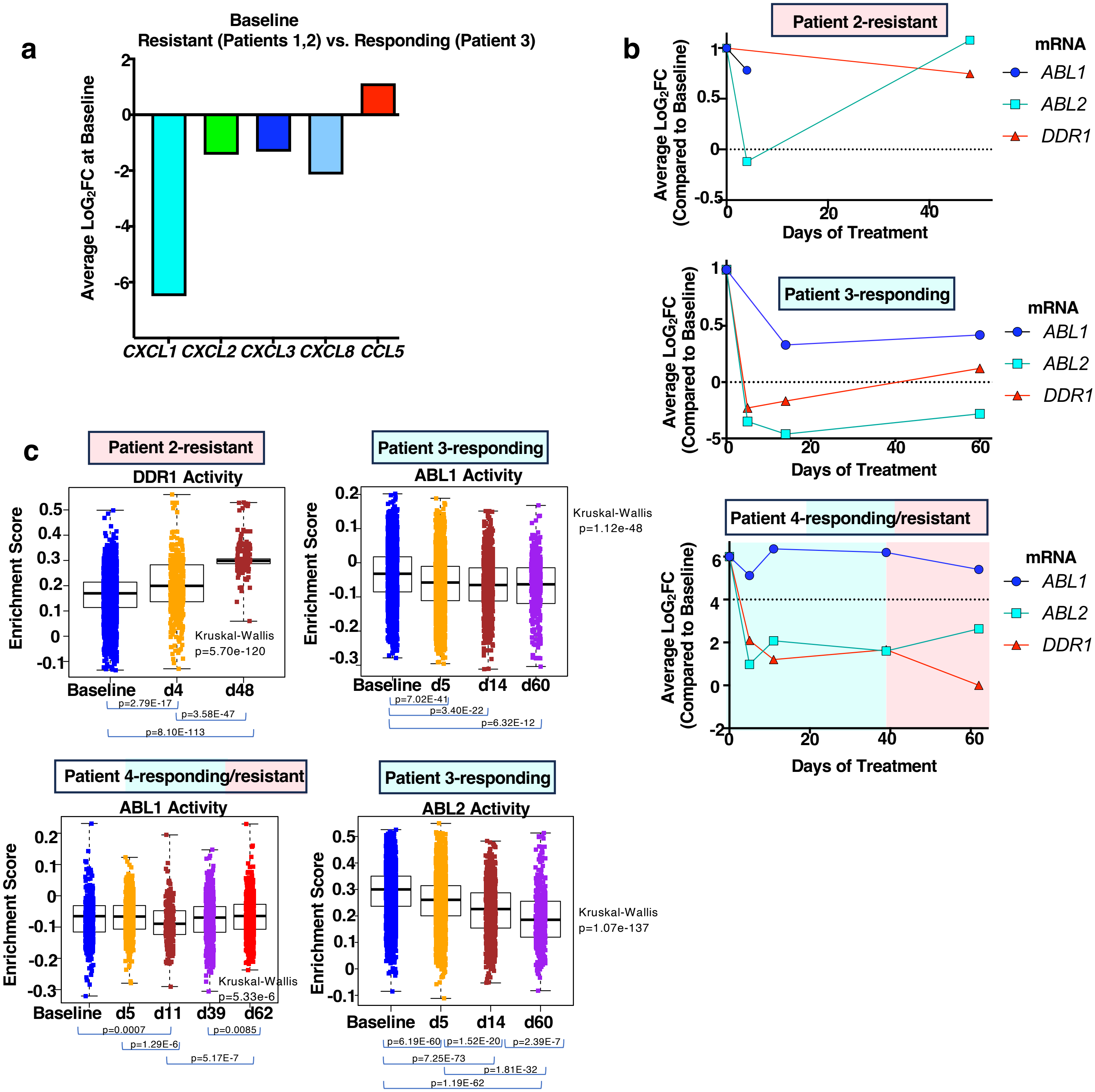

Supplementary Figure 17. Chemokine, ABL1/2, and DDR1 mRNA expression and activities in biopsies from patients prior to and during BRAFi/MEKi treatment. Support for Fig. 8. Single-cell RNAseq from patients described in Vasilevska et al. (Cohort 1) was analyzed for gene expression in the melanoma (malignant) cohort. Each patient's tumor was repeatedly sampled by fine-needle biopsy (see Fig. 8). **(a)** Comparison of CXCL expression in the baseline tumors from Patients 1,2 (resistant patients) compared to the tumor from Patient 3 (responding patient). **(b)** mRNA expression of ABL1, ABL2, and DDR1 comparing each post-treatment sample to baseline. **(c)** Sample-wise geneset enrichment analysis was used to infer ABL1, ABL2, and DDR1 kinase activities in the patient samples from Cohort 1 (see Fig. 8). Differences in cytokine expression across time points were assessed by Kruskal–Wallis tests. Pairwise comparisons were performed using two-tailed Wilcoxon rank-sum tests and adjusted with the Holm's method. Boxes show the median (center line), interquartile range (box; 25th–75th percentiles), and whiskers extend to the minimum and maximum observed values.

SUPPLEMENTARY TABLES

Table S1. Reagents Table

REAGENT or RESOURCE		SOURCE	IDENTIFIER
Antibodies	Dilution		
ABL1	1:1000	Santa Cruz Biotech (Santa Cruz, CA)	56887; clone 8E9; RRID:AB_781732
ABL2	1:1000	Millipore Sigma (St. Louis, MO)	5C6 RRID:AB_1839297
AKT (pS473)	1:1000	Cell Signaling	4060; RRID:AB_2315049
Beta-actin	1:40,000	Millipore Sigma	A5316; Clone AC-74; RRID:AB_2861213
Beta-tubulin	1:5000	Santa Cruz Biotech	66729; clone F-1; RRID:AB_ 2010699
mouse CCL2	1:500	R&D	AF-479-SP RRID: AB_354500
mouse CCL5	1:500	R&D	AF-478-SP RRID: AB_10080077
pCRKL (Y207)	1:1000	Cell Signaling	3181, 34940 RRID:AB_331068
pDDR1 (Y792)	1:1000	Cell Signaling	11994 RRID: AB_2797793
ERK (pT202/pY204)	1:3000	Promega/VWR	RB9249P; RRID:AB_430866
FOSL1 (pS265)	1:1000	Cell Signaling	3880; RRID:AB_2106922
Anti-mouse HRP	1:5000	ThermoFisher	PI31430; RRID:AB_228307
Anti-rabbit HRP	1:5000	Jackson ImmunoResearch	711-035-052
Anti-goat HRP	1:5000	Jackson ImmunoResearch	805035180
Cytokine antibody Arrays	N/A	Raybiotech	mouse C1000, C3 human C1000
Ultra-LEAF anti-mouse CD8a antibody, clone 53-6.7	200μg, i.p.	Biolegend	100764
Ultra-LEAF Rat IgG2a, k isotype control antibody	200μg, i.p.	Biolegend	400566
InVivo MAb anti-Ly6C	200μg, i.p.	BioXcell	BE0203
InVivo Mab anti-rat IgG2a	200μg, i.p.	BioXcell	BE0089
InVivo Pure pH7 dilution buffer		BioXcell	IP0070
Fc Blocking antibody (CD16/CD32)	1:50	Biolegend	101319
CD45-BB515	1:50	BD Biosciences	564585; clone HI30
CD45-APC-Cy7	1:50	Biolegend	103116; clone 30-F11
CD45-BUV395	1:50	BD Biosciences	564279; clone 30-F11
Live/Dead- Blue A-480	1:100	ThermoFisher	23105
Live/Dead-BUV496	1:100	ThermoFisher	L34962
Live/Dead-BV501	1:50	Biolegend	77143
CD3-BV605	1:50	BD Pharmingen	564009; clone 17A2
CD3-RB705	1:50	BD Biosciences	757248; clone 17A2
CD8-BV711	1:50	BD Pharmingen	563046; 53-6.7
CD8-BV711	1:50	Biolegend	100748; clone 53-6.7
CD8-BUV615	1:50	BD Biosciences	613004; clone 53-6.7
CD4-BV785	1:50	Biolegend	100551; clone RM4-5
CD4-BV750	1:50	Biolegend	100583, clone RM4-5
GR1-BV421	1:100	Biolegend	108445; clone RB6-8C5

CD11b-PE-Cy5	1:50	Biolegend	101210; clone M1/70
CD11b-PE-Cy7	1:50	Biolegend	101216; clone M1/70
Ly6C-BV650	1:50	Biolegend	128049; clone HK1.4
Ly6G-BB700	1:50	BD Biosciences	566435; clone 1A8
Ly6G-BV785	1:50	Biolegend	127645; clone 1A8
F4/80-PE-Dazzle594	1:50	Biolegend	123146; clone BM8
F4/80-PE	1:50	Biolegend	123110; clone BM8
CD206-APC	1:50	Biolegend	141707; clone C068C2
CD206-BV421	1:50	Biolegend	141717; clone C068C2
NKp46-PerCP-Fire780	1:50	Biolegend	137652, clone 29A1.4
NKp46-PE-Cy7	1:50	Biolegend	137618; clone 29A1.4
CD19-BUV661	1:50-100	BD Bioscience	565076; clone 1D3
NK1.1-BV421	1:50	Biolegend	108732; clone PK136
FOXP3-PE	1:50	Biolegend	126404; cloneMF-14
CD25-BV711	1:50	Biolegend	102049; clone PC61
Chemicals, peptides, and recombinant proteins			
Nilotinib 2nd generation ABL/DDR1b inhibitor (For *in vivo* mouse experiments)		Novartis (Basel, Switzerland)	MTA
Nilotinib (for *in vitro* experiments)		MedChemExpress	HY-10159A
GNF-5		Selleck Chem	S7526
Dabrafenib, BRAF inhibitor		Novartis (Basel, Switzerland)	MTA
Trametinib, MEK inhibitor		MedChemExpress	HY10999
DDR-IN-1, DDR1 inhibitor		MedChemExpress	13979
ECL		Pierce/ThermoFisher	PI32106
0.05% Crystal Violet Stain		Acros	EW-88226-98
methanol		VWR	BDH1135-19L
nitrocellulose		VWR	10063-173
polybrene		MilliporeSigma	H9268
hygromycin		MilliporeSigma	400051
puromycin		MilliporeSigma	P8833
G418 (geneticin)		Fisher	11811031
Commercial Kits			
CellTiter Glo		Promega/VWR	PAG7572
SYBR Green qPCR		BioRad	1725271
iScript cDNA synthesis kit		BioRad	1708891
Turbo DNase DNA-free kit		ThermoFisher	AM1907
RNAeasy		Qiagen	74104
mouse CXCL1 DuoSet ELISA		R&D Systems	DY453
Duoset Ancillary Reagent Kit (for ELISAs)		R&D Systems	DY008
Dead Cell Removal Kit (for scRNAseq)		Miltenyi	130-090-101
RBC Lysis kit (for scRNAseq)		Miltenyi	130-094-183
Tumor dissociation kit (10X dissociation buffer)		Miltenyi	130-096-730
GentleMACS C tubes		Miltenyi	130-093-237
LS Columns		Miltenyi	130-042-401
Pierce BCA Protein Assay		Fisher	A55865
Chromium Next GEM Single Cell 3' Gene Expression v3.1 kit		10X Genomics	PN-1000268
Deposited data			
RNAseq from *NRAS*-mutant melanoma patients treated with MEKi		Nagler et al. (2020) Lyon et al. (2023)	GSE198776

RNAseq from BRAF-mutant melanoma patients treated with MAPKi. Only resistant patient samples were used.	Hugo et al. (2015)	GSE65185
Single-cell RNAseq from Cohort 1 patient samples	Vasilevska et al. (2024)	GSE229908
Experimental models: Cell lines		
M14 (*BRAF*^{V600E})	NCI-60 panel	Tripathi et al. (2020)
M14-BMR (*BRAF*^{V600E})	Established in the Plattner lab	Tripathi et al. (2020)
WM164 (*BRAF*^{V600E})	Established by Meenhard Herlyn Lab	Tripathi et al. (2020)
SK-MEL-2 (*NRAS*^{Q61R})	NCI (Bethesda, MD)	NCI-60 panel, 2016
SK-MEL-2MR (*NRAS*^{Q61R})	Plattner Lab	Lyon et al.(2023)
SK-MEL-147 (*NRAS*^{Q61R})	Established by Taha Merghoub (Memorial Sloan Kettering)	This manuscript
YUMM5.2 (*Braf*^{V600E}/*Trp53*$^{-/-}$)	ATCC	Meeth et al. (2016)
YUMM5.2-BMR (*Braf*^{V600E}/*Trp53*$^{-/-}$)	This manuscript	This manuscript
YUMM1.7-*Braf*^{V600E}/*Pten*$^{-/-}$/*Cdkn2*$^{-/-}$	ATCC	Meeth et al. (2016)
YUMM1.7-BMR (*Braf*^{V600E}/*Pten*$^{-/-}$/*Cdkn2*$^{-/-}$)	This manuscript	This manuscript
YUMM3.3-*Braf*^{V600E}/*Cdkn2*$^{-/-}$	ATCC	Meeth et al. (2016)
YUMM3.3-BMR (*Braf*^{V600E}/*CDKN2*$^{-/-}$)	This manuscript	This manuscript
Ma-NRAS-1007 (*Nras*^{Q61R})	Lionel Larue (Orsay, France) via Andrew Aplin (Philadelphia, PA)	Petit et al. (2019)
MaNras1-1007-MR (*Nras*^{Q61R})	This manuscript	This manuscript
MaNras2-1014 (*Nras*^{Q61R})	Lionel Larue (Orsay, France) via Andrew Aplin (Philadelphia, PA)	Petit et al. (2019)
MaNras2-1014-MR (*Nras*^{Q61R})	This manuscript	This manuscript
OSUMMER.12 (*Nras*^{Q61R})	ATCC	Murphy et al. (2022)
OSUMMER.12MR (*Nras*^{Q61R})	This manuscript	This manuscript
OSUMMER.13 (Nras^{Q61R})	ATCC	Murphy et al. (2022)
OSUMMER.13MR (Nras^{Q61R})	This manuscript	This manuscript
293T cells	ATCC	Tripathi et al. (2020)
Experimental models: Organisms/strains		
C57BL/6J mice	JAX	Stock #: 000664
Braf/Pten mice B6.CgTg(TyrCre/ERT2)13Bos *Braf*^{tm1Mmcm} *Pten*^{tm1Hwu}/ BosJ	JAX	Stock #: 13590
Oligonucleotides and qPCR primers		
5'GGGAAATTGCTACCTATGG3'	IPTG-inducible ABL1/2 shRNA	Construct and lentivirus made by Millipore Sigma Tripathi et al. (2018) Tripathi et el. (2020)
5'CAGCCACCTTCATTCCCCAA3'	forward primer human CCL2	IDT, This manuscript
5'GGACACTTGCTGCTGGTGAT3'	reverse primer human CCL2	IDT, This manuscript
5'CGTGCCCACATCAAGGAGTA3'	forward primer human CCL5	IDT, This manuscript
5'TCGGGTGACAAAGACGACTG3'	reverse primer human CCL5	IDT, This manuscript

5'CAGGGAATTCACCCCAAGAACA3'	forward primer human CXCL1	IDT, This manuscript
5'GGATGCAGGATTGAGGCAAGC3'	reverse primer human CXCL1	IDT, This manuscript
5'CTTGTCTCAACCCCGCATCG3'	forward primer human CXCL2	IDT, This manuscript
5'TTGGATTTGCCATTTTTCAGCATC3'	reverse primer human CXCL2	IDT, This manuscript
5'CTCCAAACCTTTCCACCCCA3'	forward primer human CXCL8	IDT, This manuscript
5'TTCCTTGGGGTCCAGACAGA3'	reverse primer human CXCL8	IDT, This manuscript
5'TGGAATCTTCTCCTGGGGGT3'	forward primer human IL-6	IDT, This manuscript
5'CTTCGGTCCAGTTGCCTTCT3'	reverse primer human IL-6	IDT, This manuscript
5'CAGGTCCCTGTCATGCTTCT3'	forward primer mouse CCL2	IDT, This manuscript
5'GTGGGGCGTTAACTGCATCT3'	reverse primer mouse CCL2	IDT, This manuscript
5'GTGCCCACGTCAAGGAGTAT3'	forward primer mouse CCL5	IDT, This manuscript
5'CTCTGGGTTGGCACACACTT3'	reverse primer mouse CCL5	IDT, This manuscript
5'GACCATGGCTGGGATTCACC3'	forward primer mouse CXCL1	IDT, This manuscript
5'GACTTCGGTTTGGGTGCAGT3'	reverse primer mouse CXCL1	IDT, This manuscript
5'GAAGACCCTGCCAAGGGTTG3'	forward primer mouse CXCL2	IDT, This manuscript
5'AGGCAAACTTTTTGACCGCC3'	reverse primer mouse CXCL2	IDT, This manuscript
5'CGAAAGCATCTTGAGAGGAACA3'	forward primer human RPS13	IDT, Tripathi et al. (2020)
5'GCACCACGTCCAATGACAT3'	reverse primer human RPS13	IDT, Tripathi et al. (2020)
5'TCGTTCTCTCTTTGCTGCCA3'	forward primer mouse RPS13	IDT, This manuscript
5'CAACGTGGGGACGCTAC3'	reverse primer mouse RPS13	IDT, This manuscript
5'GGGTGGAAAACAGGGTGCTA3'	forward primer1 human ABL1 5'UTR	IDT, Tripathi et al. (2018)
5'GATGGAAAGGACATGCCCCA3'	reverse primer1 human ABL1 5'UTR	IDT, Tripathi et al. (2018)
5'GAGCCTGGCCTACAACAAGT3'	forward primer2 human ABL1 coding	This manuscript
5'CCTGGGACAGGTCAATTCCC5'	reverse primer2 human ABL1 coding	This manuscript
5'CAAGGCAGTAGTGGACAGGG3'	forward primer1 human ABL2 5' UTR	IDT, Tripathi et al. (2018)
5'ATTCCAGGTAGGGATGGGCT3'	reverse primer1 human ABL2 5'UTR	IDT, Tripathi et al. (2018)
5'AGGCTTCTTTACACCACGCT3'	forward primer2 human ABL2 coding	This manuscript
5'CCTGGGACAGGTCAATTCCC3'	reverse primer2 human ABL2 coding	This manuscript
5'GACTGGTTCGCTTCTACCCC3'	forward primer human DDR1	IDT, This manuscript

5' AGACAGGAGTCCATCCCTCC3'	reverse primer human DDR1	IDT, This manuscript
Recombinant DNA		
LV-218-mouse CCL2	GeneCopoeia	IRES2-mCherry-IRES-puro-EV-Mm05119-LV213
LV-218 empty vector	GeneCopoeia	EX-NEG-LV-213
LV-213-mouse CCL5	GeneCopoeia	IRES-Neo-EX-Mm34471-LV218
LV-218 empty vector	GeneCopoeia	EX-NEG-LV218
LV-220-mouse CXCL1	GeneCopoeia	IRES2-hygro-Mm02868-LV-220
LV-220 empty vector	GeneCopoeia	EX-NEG-LV220
pMD2.G	Addgene	12259
pRSV-Rev	Addgene	12253
pMDLg/pRRE	Addgene	12251
MIGR1-ABL1-WT		Plattner et al. (2004), Tripathi et al. (2018)
MIGR1-ABL1-PP		Plattner et al. (2004), Tripathi et al. (2018)
MIGR-ABL1 (K290R)		Plattner et al. (2004)
pcDNA-ABL2-WT		Plattner et al. (2004)
pcDNA-ABL2-(K337R)		Plattner et al. (2004)
pcDNA-ABL2-PP		Plattner et al. (2004)
siRNA and shRNA		
ABL1 siRNA	ThermoFisher	Assay #1336
ABL1 siRNA	ThermoFisher	Assay #866
ABL2 siRNA	ThermoFisher	Assay #1478
ABL2 siRNA	ThermoFisher	Assay #872
DDR1 siRNA	HorizonDiscovery	J-003111-12-0005
Software and algorithms		
SAS (v9.4)		www.sas.com
GSVA (v1.50.0)		10.18129/B9.bioc.GSVA
Cell Ranger (v6.0.0)		www.10xgenomics.com
scDBlFinder (v1.14.0)		10.18129/B9.bioc.scDblFinder
Seurat (v4.4.0)		satijalab.org/seurat
InferCNV (1.20.0)		https://github.com/broadinstitute/inferCNV
ProjectTILs (v3.0)		github.com/carmonalab/ProjecTILs
R (v4.3.1)		https://cran.r-project.org
babelgene		https://github.com/igordot/babelgene
fgsea (1.20.0)		https://github.com/alserglab/fgsea
scran		https://bioconductor.org/packages/scran/
ImageJ64 (v1.54g)	NIH	https://imagej.nih.gov/ij/
GraphPad Prism (v10,v11)	GraphPad	https://www.graphpad.com
Vassar single sample t-test		http://vassarstats.net/t_single.html

Biorender	https://biorender.com	Created in BioRender. Plattner, R. (2026) https://BioRender.com/414v3et
Primer Blast	NCBI	https://www.ncbi.nlm.nih.gov/tools/primer-blast/
CFX Manager 3.0	BioRad	
FlowJo (v10.10.0)	BD Biosciences	
SpectroFlo 3.3.0	BD Biosciences	
FACSDiva (v9.1)	BD Biosciences	
Cell Culture Reagents		
Fetal Bovine Serum	Gibco/ThermoFisher	10437028
DMEM	Corning/VWR	45000-304
RPMI	Corning/VWR	45000-396
L-15	ThermoFisher	41300-039
MCDB153	MilliporeSigma	M7403
Ham's F12	Corning/VWR<br>SigmaAldrich	10-080-CV<br>10-080-CV
Glutamine	Corning/VWR	45000-676
Trypsin-EDTA	Corning/VWR	45000-660
HEPES	Fisher	11344-041
DMEM/F12 Media	Corning/VWR	10-090-CV
MEM NEAA (non-essential amino acids)	Gibco/Fisher	11140-050
insulin	MilliporeSigma	I6634-1G
HBSS	Corning/VWR	21-022CM
Lipofectamine 2000	ThermoFisher	11668-027
PEG400	ThermoFisher	AAB219920B
Pluronic F127	Abcam	ab285413
Vitamin E TPGS	Fisher	NC9999736
IPTG	MilliporeSigma	I6758
Type IV collagenase	Worthington Biochemical	L004189
haluronidase	MP Biomedical	0210074091
DNase I	MP Biomedical	0210057580
propidium iodide/RNase staining solution	Fisher/BD Biosciences	BDB550825

LEGENDS FOR DATASETS (Excel Files)

Dataset 1. IPA genesets used for single-sample enrichment analysis.

Dataset 2. Marker genes for cell types in single-cell RNA sequencing data. Data from this table was used to generate Supplementary Fig. 9c. Tab 1. B cells. Tab 2. CD4$^+$ T cells. Tab 3. CD4/CD8-negative T cells. Tab 4. CD8$^+$ T cells. Tab 5. classical DCs (cDCs). Tab 6. myeloid dendritic cells (mDCs). Tab 7. plasmacytoid dendritic cells (pDCs). Tab 8. monocytes. Tab 9. macrophages. Tab 10. neutrophils. Tab 11. neutrophils sorted by fold change. Tab 12. NK cells. Tab 13. Fibroblasts. Tab 14. endothelial cells. Tab 15. Mast cells. Tab 16. Melanoma cells. Tab 17. Dying melanoma cells. Tab 18. smooth muscle cells.

Dataset 3. Differential expression analysis in single-cell RNA sequencing data comparing D/T+nilotinib versus D/T-treated tumors. Differentially expressed genes comparing mice treated with D/T+nilotinib to those treated with D/T alone. Tab 1. All immune cells p-values. Tab 2. Differentially expressed genes in CD4$^+$ T cells. Positive values indicate that the genes are upregulated in nilotinib-treated animals. Tab 3. Differentially expressed genes in CD8$^+$ T cells. Tab 4. Differentially expressed genes in neutrophils. Tab 5. Differentially expressed genes in melanoma cells. Tab 6. Differentially expressed genes in the dying melanoma population.

Dataset 4. Marker genes for T cell subtypes in single-cell RNA sequencing data. Data from this table was used to generate Fig. 7b. Tab 1. CD8 Effector Memory. Tab 2. $CD8^+$ Tpex. Tab 3. $CD8^+$ Tex. Tab 4. $CD8^+$ Early Active. Tab 5. $CD8^+$ Naive-like Tab 6. $CD4^+$ Tregs. Tab 7. $CD4^+$ Th1. Tab 8. $CD4^+$ Naive-like. Tab 9. $CD4^+$ Tfh.

Dataset 5. Differential expression analysis of $CD4^+$ and $CD8^+$ T cell populations by single-cell RNA sequencing. Tab 1. $CD8^+$ effector memory cells. Tab 2. $CD8^+$ Early Active cells. Tab 3. $CD8^+$ Tpex cells. Tab 4. $CD8^+$ Naive-like cells. Tab 5. $CD4^+$ Tregs. Tab 6. $CD4^+$ Th1 cells. Tab 7. $CD4^+$ Naive-like cells. Tab 8. $CD8^+$ T cell subset p-values. Tab 9. $CD4^+$ T cell subset p-values.

Dataset 6. Geneset enrichment analysis (GSEA) of $CD4^+$ and $CD8^+$ T cell populations by single-cell RNA sequencing. Tab 1. GSEA KEGG Pathway analysis of $CD8^+$ T cells; Tab 2. GSEA HALLMARK Pathway ($CD8^+$ T cells); Tab 3. GSEA REACTOME Pathway ($CD8^+$ T cells)

Dataset 7. Differential expression analysis of data from Cohort 1 (Vasilevska et al. 2024). Tab 1=comparison of Patient 3 (baseline) to Patients 1,2 (baseline). Tabs 2-5. Comparison of post-treatment (PT) samples to baseline. Tab 2.Patient 1; Tab 3=Patient 2; Tab 4. Patient 3; Tab 5. Patient 4.
