# Supplementary material for: Targeting ABL kinases overcomes melanoma immune evasion following resistance to MAPK inhibition
Source: Nat Commun. 2026 Aug 21;17:9995. doi: 10.1038/s41467-026-76864-y (PMC13590603; doi:10.1038/s41467-026-76864-y)
Supplement: Supplementary file 2 [file 41467_2026_76864_MOESM2_ESM.pdf]

nature portfolio

Peer Review File

Targeting ABL kinases overcomes melanoma immune evasion following resistance to MAPK inhibition

Corresponding Author: Dr Rina Plattner

This file contains all reviewer reports in order by version, followed by all author rebuttals in order by version.

Version 0:

Reviewer comments:

Reviewer #1

(Remarks to the Author)
Tripathi R. et al. demonstrated that ABL1/2 kinases contribute to MAPK inhibitor resistance in metastatic melanoma by promoting both tumor cell survival and immune suppression through cytokine secretion. They showed that targeting ABL1/2 reduces immunosuppressive myeloid cell infiltration and enhances CD8⁺ T cell activity, making ABL kinases promising therapeutic targets. While the manuscript presents extensive in vitro and in vivo data, the current evidence is not sufficient to fully support the authors' conclusions.

Major points
1. To support the in vivo significance of cytokines derived from drug-resistant tumor cells, please perform in vivo experiments similar to those shown in Figures 5a–c, using BMR cells with knockouts of CXCL1, CCL5, or CCL2.
2. Figure 1:
Please include a comparison between parental and resistant cell lines to clarify whether cytokine secretion is elevated in resistant cells.
Additionally, similar to Figure 1f, please demonstrate whether ABL overexpression (ABL-OE) alone is sufficient to increase cytokine secretion in BRAF-mutant cells.2.
3. Figure 2:
Add qRT-PCR data from parental cells for comparison.
Modify Figure 2F to improve clarity and make the data easier to interpret.3.
4. Figure 3:
Include data from conditions without drug treatment to directly support the claim of drug resistance.
5. Figure 4:
Although nilotinib reduces M-MDSC populations, Figure 4a and 4b show no difference in M-MDSC infiltration between YUMM5.2 and YUMM5.2-BMR. Please explain or discuss the mechanism underlying resistance in this context.5.
6. Figure 5:
Include Ly6C^hi population data alongside Ly6G⁺ data in Figures 5e–i to better support the proposed MDSC-related resistance mechanism.
7. Supplemental Figure 2a and 2b:
Add data from parental cells with and without D/T treatment.
Also include control data without D/T treatment for BMR and MR cells.
8: Supplemental Figure 2e/f:
The authors previously reported that ABL1/2 induces pERK via the CRAF-ERK pathway, but nilotinib only faintly reduces pERK in these figures. Please discuss alternative mechanisms that may influence cytokine secretion beyond the ERK pathway.

Minor points:
1. Please clearly label the cell lines used in each figure. For example, Figure 4b likely uses YUMM5.2-BMR, although the legend states YUMM5.2, which is confusing.

Reviewer #2

(Remarks to the Author)
This is a comprehensive and well-executed study that provides important insights into how the melanoma secretome contributes to tumour microenvironment (TME) remodelling and influences responses to targeted and immune-based therapies. The cytokine overexpression experiments in mouse models are particularly compelling and support the mechanistic framework proposed by the authors.

However, several issues limit the clarity and interpretability of the findings. There is substantial background information embedded within the Results section, which makes critical data difficult to follow; much of this contextual narrative would be more appropriately placed in the Introduction or Discussion. In addition, although the dependence on $CD8^+$ T cells is expected, and the Ly6 depletion data should include tumour volume figure as in Figure S13 – how does the tumour volume curves compare to DT-Nilo. Did the authors develop DTNilo resistance models?

A further important omission is the lack of investigation into how cell-cycle status may influence the observed transcriptomic and cytokine changes. Nilotinib is known to induce cell-cycle arrest, raising the possibility that alterations in the secretome may reflect secondary effects of cell-cycle perturbation rather than direct consequences of ABL1/2 inhibition. In Figure 1f, for instance, it is unclear whether the observed cytokine alterations are independent of cell-cycle effects. The authors should determine whether ABL1/2 knockdown induces cell-cycle arrest, and if so, whether this contributes to the cytokine expression changes.
The manuscript does not provide the gene signatures used to compute GSVA activity scores for ABL1, ABL2, and DDR1. As these represent arbitrary scoring systems, the full signatures must be included as Supplementary Data. Some evidence of validation—for example, changes in signature scores in control vs. siABL cells—would further support the robustness of this approach.
Addressing these comments will be essential to fully interpret the mechanism proposed by the authors.

Additional minor comments
1. Supplementary Figure 1 – absolute quantification required.
The data in Supplementary Figure 1 are presented as relative levels; however, absolute quantification (e.g., ng/mL) should be provided to allow full interpretation of cytokine abundance and biological relevance.
2. Figure 1c is described as "trametinib ± nilotinib," but the figure panels show shVector, shAA, and D/T conditions. The legend and panel labels are inconsistent with the stated experimental conditions.
3. Figure 1d references siABL1 in the displayed data, but this is not described in the figure legend.A clear, consistent description of the experimental design and conditions in Figure 1 is required.
4. Figure legends require substantial improvement.
Many legends lack sufficient detail and include undefined acronyms (e.g., BMR, MR, DDR in Fig. S2). Even if defined in the main text, each legend should be fully self-contained. All abbreviations must be defined, and the legend should concisely explain what each figure shows, including experimental context and key outcomes.
5. Normalisation of array data requires detailed description
The method used to normalise cytokine array data is not described but is critical for reproducibility. The authors should detail how exposure times for chemiluminescence were controlled, whether the same acquisition settings were used across all arrays, and whether internal control proteins were used to select images for quantification. Without this, the reliability of quantitative comparisons is difficult to assess.

Reviewer #3

(Remarks to the Author)
The study by Tripathi et al. investigates the role of ABL1/2 kinases in mediating immune evasion and resistance to MAPKi in melanoma. The authors report that nilotinib, an ABL1/2 inhibitor, can overcome MAPKi resistance by promoting $CD8^+$ T cell infiltration and reducing myeloid-derived suppressor cell (MDSCs) infiltration. Mechanistically, they show that ABL1/2 kinases drive the secretion of immunosuppressive chemokines, including CXCL1, CCL2, and CCL5, which attract MDSCs into the tumor microenvironment. Nilotinib treatment can reduce the accumulation of $Ly6G^+$ and Ly6Chi MDSCs, thereby improving tumor control/immune response and preventing resistance. These effects depend on $CD8^+$ T cells, as their depletion abrogates the protective effect of nilotinib. The authors also attempt to validate their findings in melanoma patients, demonstrating that CXCL1 and CCL5 levels correlate with resistance to MAPKi. This supports the notion that ABL1/2-driven chemokine secretion contributes to therapeutic escape in patients. Altogether, the study proposes that targeting ABL1/2 kinases may have dual therapeutic benefits: direct inhibition of tumor growth and modulation of the immune microenvironment to overcome resistance.
Overall, the study addresses a relevant and timely question, and the findings are potentially significant. However, several aspects of the manuscript require clarification, additional data, and improved presentation. Some sections are difficult to follow, and improving the clarity and flow of the text would enhance overall readability. Therefore, the study appears preliminary in its current form. Below are my detailed comments:
1. The activation of ABL1/2 and DDR1 upon the development of resistance to D/T should be validated in both murine and human BRAF- and NRAS-mutant cells used in this study.
2. Cell viability assays for BMR and MR human cells, with and without nivolumab, are missing and should be included.
3. Figures 1a and 1c do not include CCL5 and CCL2. These cytokines appear to be relevant only in NRAS-mutant human cells. The authors should at least explain why the expression of these cytokines is restricted to NRAS-mutant cells.
4. Figures 1d and 1e are potentially misleading. The text states that D/T treatment was applied to BRAF-mutant cells and

trametinib (T) to NRAS-mutant cells following ABL and DDR depletion. These treatment conditions should be clearly indicated in the figures.
5. Increasing the signal intensity in cytokine arrays reveals additional cytokines in Figure 1c and Suppl. Figure 3b-d. Because the signal is enhanced to detect specific cytokines, the authors should also consider and report the newly detected ones.
6. What is the basal level of cytokines in the different BRAF/NRAS mutant cell lines?
7. Data presentation in Figure 1 and 2 should be better organized (e.g. Fig. 2 uses too many discordant colors, reducing visual clarity).
8. The correlation analysis performed on TCGA samples provides limited insight into MAPKi resistance, which is the main focus of Figures 1 and 2. The authors should repeat the analysis in datasets from MAPKi-resistant patients.
9. Do the Figure 2 panels refer to the same experimental conditions used for the cytokine secretion assays? The fig. legends suggest otherwise, and the rationale for using different settings should be clarified. As currently presented, RNA expression is measured after 24 h of nilotinib treatment, whereas cytokine secretion is assessed after 12 h, which appears counterintuitive.
10. In Suppl. Figure 2C, it is unclear why BMR cells, although resistant to MAPKi, continue to grow under nilotinib treatment. Also, the rationale for using the GNF5 inhibitor instead of nilotinib in Suppl. Figure 2d should be explained, and why is it not effective? In NRAS-mutant murine cell lines, ABL inhibition alone appears ineffective, further clarification is needed.
11. In Suppl. Figure 3, cytokine expression in OSUMMER cells is not clearly represented. Some dots highlighted in squares do not correspond to the cytokines indicated. Panels a–d are confusing, and the array image overexposed may lead to misinterpretation of cytokine secretion patterns.
12. In Fig. 2 a, b and Suppl. Figure 3 e-h, downregulation at the mRNA level of some cytokines (Ccl5 in BRAF- and NRAS-mutant cells, Cxcl5 in NRAS-mutant cells) is not observed upon Nilotinib, despite their reduced secretion (Figure 1 and Suppl. Figure 3 b, c). Conversely, cytokine Cxcl5 downregulation observed by RNA-seq in BRAF-mutant cells (Suppl. Figure 3i) is not secreted in a consistent manner (Suppl. Figure 3a,b). Overall, the focus should be on secreted cytokines found in both human and mouse cells, and their relationship with immune cell infiltration, rather than on cytokine expression regulation, as the cytokine expression and secretion are not consistent in many cases. Moreover, it would be valuable to investigate how ABL1/2 mechanistically drive cytokine secretion. Is cytokine release dependent solely on MAPK pathway activation, or are additional signaling pathways involved?
13. The authors should assess ABL activity in mouse tumors following the different treatments, at least in some models. Moreover, all tumor growth data should be presented consistently, either as fold change in tumor volume or as absolute volume over time.
14. The authors demonstrate that the combination of D/T and nilotinib reduces MDSC infiltration leading to better tumor control and overcoming resistance to MAPKi. In that case it would be interesting to explore how exactly ABL1/2 inhibition influences the differentiation or recruitment of MDSCs. Are there any specific signaling pathways or transcription factors that mediate these changes?
15. In this study the authors focus on specific immune cell populations like MDSCs and CD8+ T cells. But how might CXCL1, CCL2, and CCL5 expression affect other tumor-infiltrating immune populations (e.g., NK cells, Tregs etc.)? CCL5 is a cytokine that has been associated with the accumulation of Tregs.
16. In Figure 5a-d, tumors derived from mice treated with D/T+nilotinib were too small to be analyzed via flowcytometric analysis, so blood and spleen were used as proxies for tumor immune infiltration. MDSCs are immune cells that can suppress the anti-tumor immune responses. But MDSCs in the blood or spleen may not behave the same as MDSCs infiltrating the tumor side. They may differ in their trafficking and in their survival. So, by measuring MDSCs in the blood/spleen may not accurately reflect local immunosuppression inside the tumor. This limitation should be clearly acknowledged.
17. Paragraphs describing Figure 6 should be merged. Panels 6f–h and 6g–i should be combined to facilitate direct comparison between vehicle, D/T (or T), and D/T (or T) + nilotinib treatments.
18. The study demonstrates that nilotinib treatment in combination with MAPKi leads to increased infiltration of cytotoxic CD8+ T cells and N1-like neutrophils, with the latter identified based on transcriptional profiles and expression of activation markers (Cebpb, Slpi). However, while CD8+ T cell depletion abolishes the anti-tumorigenic effect of nilotinib, the functional contribution of the N1 neutrophils remains unclear. Are these N1-like neutrophils truly cytotoxic in vivo, or do they reflect only a transcriptional signature without functional relevance? Further experimental evidence to validate their cytotoxic potential should be provided. Is the effect of N1-like neutrophils entirely dependent on CD8+ T cells?
19. The study primarily assesses short-term (4-day) treatment effects. Would CD8+ T cell and N1 neutrophil infiltration or function persist upon prolonged treatment?
20. The legend corresponding to Figure 7g–i is missing and should be added.

Version 1:

Reviewer comments:

Reviewer #1

(Remarks to the Author)
The authors have clarified my concerns and are congratulated to carry on this study.

Reviewer #2

(Remarks to the Author)
The authors have adequately addressed all of my comments and have substantially improved the manuscript. I have no

additional comments.

Reviewer #3

(Remarks to the Author)
My comments on the revised manuscript and the authors' responses are provided below:

R1. The activation of ABL1/2 in BRAF-mut cells appears modest and not fully convincing in Suppl. Fig. 2. The reproducibility of this observation is unclear and requires further validation. As the main effect of inhibiting ABL kinases is here described in murine models, a consistent signal of their activation is expected. Moreover, the ability of nilotinib to suppress this pathway should be confirmed in murine cell lines.

R7, Figure 2. The green dots are not optimal for visualization and should be replaced with a more suitable color palette. Colors should also be harmonized across panels to improve clarity, contrast, and overall visual consistency.

R9. The explanation provided remains insufficient. RT–qPCR analyses should be performed at earlier time points, in line with the cytokine secretion assays, to ensure temporal alignment and to support a more accurate interpretation of the data.

R14,18,19. The authors have not addressed the concerns raised, as no additional experiments or analyses have been provided. Overall, the responses would benefit from a more thorough and constructive approach.

Paragraph "ABL1/2 activities correlate with cytokine expression in patient samples" (page 6): The data support an association rather than a causal relationship. The text should be revised accordingly to avoid overinterpretation.
Paragraphs "Nilotinib impacts infiltration of T cell subsets" and "Nilotinib treatment influences CD8+ T cell expression profiles" (page 12, 13): these sections could be merged into a single, more concise paragraph to improve flow and coherence.
Paragraph "CXCL1 expression and ABL1/2 activities are elevated during BRAFi/MEKi resistance in patients" (page 14): This section lacks clarity and does not consistently refer to the relevant figures. Figure citations should be clearly indicated, and the text revised.

General comment: A thorough revision of the language is recommended throughout the manuscript, including the Discussion.

Version 2:

Reviewer comments:

Reviewer #3

(Remarks to the Author)
I do not have further comments, the authors have addressed all my concerns.

Open Access This Peer Review File is licensed under a Creative Commons Attribution 4.0 International License, which permits use, sharing, adaptation, distribution and reproduction in any medium or format, as long as you give appropriate credit to the original author(s) and the source, provide a link to the Creative Commons license, and indicate if changes were made.
In cases where reviewers are anonymous, credit should be given to 'Anonymous Referee' and the source.
The images or other third party material in this Peer Review File are included in the article's Creative Commons license, unless indicated otherwise in a credit line to the material. If material is not included in the article's Creative Commons license and your intended use is not permitted by statutory regulation or exceeds the permitted use, you will need to obtain permission directly from the copyright holder.
To view a copy of this license, visit https://creativecommons.org/licenses/by/4.0/

RESPONSE TO REVIEWERS' COMMENTS

We thank the reviewers for their careful evaluation of our manuscript. We were pleased that the reviewers noted that the manuscript was "comprehensive", included "extensive data", and investigated an "important and timely question". We respond in full to all Reviewer comments and suggestions below.

Reviewer #1.
Major points
1. To support the in vivo significance of cytokines derived from drug-resistant tumor cells, please perform in vivo experiments similar to those shown in Figures 5a–c, using BMR cells with knockouts of CXCL1, CCL5, or CCL2.

We attempted to silence CXCL1, CCL2 or CCL5 in YUMM5.2-BMR cells using lentiviral shRNAs. We found it very difficult to get efficient knockdown for CXCL1 or CCL2, observing <50% knockdown with two independent shRNAs (see Appendix Fig. 1A-C). Moreover, although we were initially able to efficiently silence CCL5 and made a stable cell line, the knockdown was dramatically lost over time such that after expansion and preparation for injection into animals, the gene was either no longer silenced or the knockdown was extremely inefficient (see Appendix Fig. 1A-C). In addition, interestingly, CCL2 expression became dramatically elevated in cells expressing CCL5 shRNA, indicating compensation (Appendix, Fig. 1D). Since there was a selective disadvantage to losing expression of these cytokines, *in vitro*, it was not possible to perform the suggested experiment. That being said, the proposed experiment will only test whether the cytokines are required for resistance using cells that acquired resistance, *in vitro* and additionally will not test the ABL1/2 dependence. The experiment we already performed (rescue experiment; Fig. 5) shows that re-expression of these cytokines in parental cells rescues the ability of nilotinib to reverse resistance that develops, ***in vivo,*** as well as MDSC infiltration. We believe that the experiment we already performed tests the more relevant question which is whether the cytokines are critical for the onset of resistance, *in vivo*, and additionally whether these cytokines are required for the ABL1/2-driven effects on resistance and MDSC infiltration.

2. Figure 1 and 2.
Please include a comparison between parental and resistant cell lines to clarify whether cytokine secretion is elevated in resistant cells. Add qRT-PCR data from parental cells for comparison.
Additionally, similar to Figure 1f, please demonstrate whether ABL overexpression (ABL-OE) alone is sufficient to increase cytokine secretion in BRAF-mutant cells.

We include new data (new Figs. 1h,i and 2f,g) comparing cytokine expression between parental and resistant cells using cytokine arrays and RT-qPCR, and added a BRAF-mutant cell line engineered to express constitutively active ABL1 and ABL2 (new Fig. 1f).

Modify Figure 2F to improve clarity and make the data easier to interpret.

Since we now show RT-qPCR comparing parental and resistant cell lines (new Fig. 2f,g), we removed the old Fig. 2f (RNA sequencing).

4. Figure 3.
Include data from conditions without drug treatment to directly support the claim of drug resistance.

We added these data to the tumor graphs in Fig. 3.

5. Figure 4:
Although nilotinib reduces M-MDSC populations, Figure 4a and 4b show no difference in M-MDSC infiltration between YUMM5.2 and YUMM5.2-BMR. Please explain or discuss the mechanism underlying resistance in this context.5.

These two experiments were performed at different times and so, the percentages cannot be directly compared. There are a number of variables that change from experiment to experiment, which makes it difficult to compare percentages from one experiment to the other (e.g. mice, tumor growth rate, etc.). Unfortunately,

we cannot perform these experiments at the same time as the YUMM5.2-BMR tumors grow much more slowly in mice than the parental line.

6. Figure 5:
Include Ly6C^hi population data alongside Ly6G$^+$ data in Figures 5e–i to better support the proposed MDSC-related resistance mechanism.

These data were added to new Supplementary Fig. 8.

7. Supplemental Figure 2a and 2b:
Add data from parental cells with and without D/T treatment (colony assays).

These data were added to new Supplementary Fig. 2b,c.

Also include control data without D/T treatment for BMR and MR cells.

Resistant cells are maintained in D/T (BMR; *BRAF*-mutant) or trametinib (MR; *NRAS*-mutant). Many resistant cell lines become addicted to the drugs, and drug removal can cause cell death.[1] Moreover, the drugs are needed to maintain resistance as reversion can occur if the drugs are removed. Due to these two points, we no longer remove BRAFi and/or MEKi from the media of resistant cells.

8: Supplemental Figure 2e/f:
The authors previously reported that ABL1/2 induces pERK via the CRAF-ERK pathway, but nilotinib only faintly reduces pERK in these figures. Please discuss alternative mechanisms that may influence cytokine secretion beyond the ERK pathway.

Addition of nilotinib to D/T or trametinib induces dramatic reduction of ERK reactivation in all cell lines (new Supplementary Fig. 2f), indicating that nilotinib reverses MAPKi resistance, which is consistent with our published data in human cells.[2,3] We also observe reduction in pAKT in the presence of nilotinib (+trametinib or D/T) in these mouse lines (new Supplementary Fig. 2), which indicates that ABL1/2 may also promote resistance by driving activation of this pathway as well. Nilotinib alone (without MAPKi) has little effect as the presence of MAPKi is what drives ABL1/2 activation and resistance signaling. In order to reduce confusion and since we no longer remove MAPKi from the media of our resistant cells, the nilotinib alone data (no MAPKi) was removed from this figure.

Minor points:
1. Please clearly label the cell lines used in each figure. For example, Figure 4b likely uses YUMM5.2-BMR, although the legend states YUMM5.2, which is confusing.

We added cell line labels to all figures containing data from multiple cell lines, and made sure that the legends are consistent with the labeling of the figures.

Reviewer #2.
1. There is substantial background information embedded within the Results section, which makes critical data difficult to follow; much of this contextual narrative would be more appropriately placed in the Introduction or Discussion.

We moved these narratives from the "Results" section to the "Introduction" and "Discussion".

2. In addition, although the dependence on CD8$^+$ T cells is expected, and the Ly6 depletion data should include tumour volume figure as in Figure S13 – how does the tumour volume curves compare to DT-Nilo. Did the authors develop DTNilo resistance models?

The tumor curves have been added to the new Supplementary Fig.7b. The effects of Ly6C depletion are less dramatic than what we observe for nilotinib. This is likely because it is notoriously difficult to deplete Ly6C^{hi} (or Ly6G$^+$) cells due to rapid regeneration from the bone marrow and reduced marker cell surface expression in

immature cells, which leads to escape from depletion.[4] Indeed, we only observe 50% of Ly6C cells depleted (see new Fig. 4e).

We did not develop D/T+nilotinib resistance models as we are investigating mechanisms governing BRAFi/MEKi resistance rather than resistance to nilotinib treatment.

3. A further important omission is the lack of investigation into how cell-cycle status may influence the observed transcriptomic and cytokine changes. Nilotinib is known to induce cell-cycle arrest, raising the possibility that alterations in the secretome may reflect secondary effects of cell-cycle perturbation rather than direct consequences of ABL1/2 inhibition. In Figure 1f, for instance, it is unclear whether the observed cytokine alterations are independent of cell-cycle effects. The authors should determine whether ABL1/2 knockdown induces cell-cycle arrest, and if so, whether this contributes to the cytokine expression changes.

Cytokine array screening was performed after short-term serum-starvation and nilotinib treatment (12-16h). The manufacturer of the cytokine arrays does not recommend using serum in the media as its presence causes high background. Conversely, RT-qPCR was assessed in serum conditions 24h after nilotinib treatment or 72h after siRNA transfection or 6 days after IPTG induction (for ABL1/2 shRNA). The timepoints for cytokine array screens and qPCR were chosen in order to maximize ABL1/2 inhibition while also assessing changes prior to ABL1/2 si/shRNA or inhibitors causing cell death. Indeed, these timepoints result in >90% viable cells at the time of harvest. This point was clarified in the manuscript (see pg.5).

We examined the percentage of cells in various stages of the cell cycle using the same conditions we used for cytokine arrays and RT-qPCR studies (above). We observed only small changes in cell cycle distribution (see Supplementary Fig. 1g,k) after silencing/inhibiting ABL1/2 at the timepoints indicated above, and the treatments/transfection did not prevent cycling as evidenced by the presence of S-phase cells. Thus, the ABL1/2-dependent effects on cytokine secretion and mRNA expression that we observe are independent of their effects on the cell cycle.

4. The manuscript does not provide the gene signatures used to compute GSVA activity scores for ABL1, ABL2, and DDR1. As these represent arbitrary scoring systems, the full signatures must be included as Supplementary Data. Some evidence of validation—for example, changes in signature scores in control vs. siABL cells—would further support the robustness of this approach.

The target genelists are now provided in Supplementary Dataset 1. Moreover, we used RT-qPCR to validate genes from the ABL1 and ABL2 gene signatures in cells expressing ABL1/2 shRNA. These data are now included in Fig. 2l.

Additional minor comments

1. Supplementary Figure 1 – absolute quantification required.

The data in Supplementary Figure 1 are presented as relative levels; however, absolute quantification (e.g., ng/mL) should be provided to allow full interpretation of cytokine abundance and biological relevance.

These are semi-quantitative membrane cytokine antibody arrays from Raybiotech that use chemiluminescence for detection. As indicated by Technical Support at Raybiotech, there is no way to express the data in ng/ml as standards are not included with these arrays, and the data can only be presented as relative values. The method for exposure and quantitation is now detailed in the Methods section (pg. 23).

Comments related to the Figure Legends.

1. Figure 1c is described as "trametinib ± nilotinib," but the figure panels show shVector, shAA, and D/T conditions. The legend and panel labels are inconsistent with the stated experimental conditions.

Figure 1d references siABL1 in the displayed data, but this is not described in the figure legend. A clear, consistent description of the experimental design and conditions in Figure 1 is required.

Many legends lack sufficient detail and include undefined acronyms (e.g., BMR, MR, DDR in Fig. S2). Even if defined in the main text, each legend should be fully self-contained. All abbreviations must be defined, and the legend should concisely explain what each figure shows, including experimental context and key outcomes.

Figure legends require substantial improvement.

We apologize for the lack of clarity in the figure legends. The legends in the revised manuscript have now been altered to increase clarity, all abbreviations are now defined, and experimental conditions have been clarified along with key outcomes.

2. Normalisation of array data requires detailed description
The method used to normalise cytokine array data is not described but is critical for reproducibility. The authors should detail how exposure times for chemiluminescence were controlled, whether the same acquisition settings were used across all arrays, and whether internal control proteins were used to select images for quantification. Without this, the reliability of quantitative comparisons is difficult to assess.

As described above, the method for normalization is now included in the methods section (pg. 23). Membranes to be compared were processed at the same time, exposed to film simultaneously, and quantitated via ImageJ comparing duplicate spot intensities to control spots. The control spots use for quantitation are now clearly marked in all figures. Moreover, original (uncropped) blots are shown in the Source Data File.

Reviewer #3.
1. The activation of ABL1/2 and DDR1 upon the development of resistance to D/T should be validated in both murine and human BRAF- and NRAS-mutant cells used in this study.

For the human cell lines, this data was already shown in our two manuscripts,[2,3] (referenced in the Results Section, pgs. 3, 7), and thus, cannot be repeated here. We provide new data for the mouse cell lines in new Supplementary Fig. 2a.

2. Cell viability assays for BMR and MR human cells, with and without nilotinib, are missing and should be included.

This data for the human cells was already shown in our two manuscripts,[2,3] and thus, cannot be reproduced in this manuscript. We reference the data in the Results section (pg. 7).

3. Figures 1a and 1c do not include CCL5 and CCL2. These cytokines appear to be relevant only in NRAS-mutant human cells. The authors should at least explain why the expression of these cytokines is restricted to NRAS-mutant cells.

CCL2 is massively expressed in M14-BMR; and thus, it is difficult to assess changes with nilotinib or shRNA treatment (Fig. 1a,c) as the film exposure times are very short. We replaced the exposure shown with a shorter exposure (for D/T vs. D/T+nilotinib) so one can see that there is a reduction in CCL2; however, given the short exposure time, the change in expression is likely under-represented. Indeed, CCL2 is highly overexpressed in M14-BMR compared to the parental cell line (see new Fig. 1h). CCL5 is expressed at a lower level, and is reduced by nilotinib treatment (see new darker exposure and quantitation in Supplementary Fig. 1a). Importantly, nilotinib reduces CCL2 and CCL5 in multiple *BRAF*-mutant mouse cell lines (see Supplementary Fig. 3). So, while we agree that ABL1/2 appear to have more dramatic effects on CCL2/CCL5 in *NRAS*-mutant cell lines, BRAF-mutant lines are also impacted, which is why we chose to follow-up on these changes in the manuscript. Indeed, CCL2 and CCL5 secretion are required for nilotinib to prevent resistance in the YUMM5.2 BRAF-mutant mouse model (see Fig. 5a-c). These points were added to the manuscript (see pg. 5).

4. Figures 1d and 1e are potentially misleading. The text states that D/T treatment was applied to BRAF-mutant cells and trametinib (T) to NRAS-mutant cells following ABL and DDR depletion. These treatment conditions should be clearly indicated in the figures.

Resistant cells are maintained in the drug(s) that were used to create resistance for all experiments. *BRAF*-mutant BRAFi/MEKi-resistant cells (BMR) were maintained in D/T, whereas *NRAS*-mutant MEKi resistant cells were maintained in trametinib. The drugs are needed to maintain resistance as reversion or cell death can occur if the drugs are removed. The indicated figure legend was amended to more accurately reflect the conditions used.

5. Increasing the signal intensity in cytokine arrays reveals additional cytokines in Figure 1c and Suppl. Figure 3b-d. Because the signal is enhanced to detect specific cytokines, the authors should also consider and report the newly detected ones.

We did not include all of the cytokine data that were changed as we chose to focus this manuscript on specific cytokines that were consistently changed across multiple human and mouse cell lines. We included the rest of the cytokine changes for the reviewer in Appendix, Fig. A2 and would be happy to include the data if the reviewer wants it included. However, we believe that including all of these other secreted factors would reduce the focus and increase the complexity of the manuscript, thereby making the manuscript more difficult for readers to follow.

6. What is the basal level of cytokines in the different BRAF/NRAS mutant cell lines?

We have now compared cytokine secretion (via antibody arrays) from parental and resistant cells to determine the basal cytokine expression in parental cells relative to resistant cells. The data is shown in the new Fig. 1h,i and Fig. 2f,g.

7. Data presentation in Figure 1 and 2 should be better organized (e.g. Fig. 2 uses too many discordant colors, reducing visual clarity).

The Figures were reorganized and the color intensity was reduced in order to increase visual clarity.

8. The correlation analysis performed on TCGA samples provides limited insight into MAPKi resistance, which is the main focus of Figures 1 and 2. The authors should repeat the analysis in datasets from MAPKi-resistant patients.

We already had included data from a small *NRAS*-mutant MEKi-treated patient dataset in the prior version of the manuscript (n=23, new Fig. 2j). We now include additional data from a dataset of patients with BRAF-mutant melanomas that were treated with MAPKi, and we limited our analyses to only those patients who demonstrated resistance (see Fig. 2k and method on pgs. 24-5). Moreover, we have an entire additional figure devoted to assessing cytokine expression and ABL/DDR1 activation in patients whose BRAF-mutant tumors were sampled prior to and after resistance (see Fig. 8).

9. Do the Figure 2 panels refer to the same experimental conditions used for the cytokine secretion assays? The fig. legends suggest otherwise, and the rationale for using different settings should be clarified. As currently presented, RNA expression is measured after 24 h of nilotinib treatment, whereas cytokine secretion is assessed after 12 h, which appears counterintuitive.

Cytokine array screening was performed after short-term serum-starvation and nilotinib treatment (12-16h). The manufacturer of the cytokine arrays does not recommend using serum in the media as its presence causes high background. Conversely, RT-qPCR was assessed in serum conditions 24h after nilotinib treatment or 72h after siRNA transfection or 6 days after IPTG induction (for ABL1/2 shRNA). The timepoints for cytokine array screens and qPCR were chosen in order to maximize ABL1/2 inhibition while also assessing changes prior to ABL1/2 si/shRNA or inhibitors causing cell death. Indeed, these timepoints result in >90% viable cells at the time of harvest. This point was clarified in the manuscript (see pg.5).

10. In Suppl. Figure 2C, it is unclear why BMR cells, although resistant to MAPKi, continue to grow under nilotinib treatment. Also, the rationale for using the GNF5 inhibitor instead of nilotinib in Suppl. Figure 2d should be explained, and why is it not effective? In NRAS-mutant murine cell lines, ABL inhibition alone appears ineffective, further clarification is needed.

We are sorry that the rationale for these experiments was not clear. In the introduction of the manuscript (pg. 3), we describe our previous published data showing that MEKi resistance in *NRAS*-mutant melanoma is driven by ABL1/2 and DDR1,[2] whereas ABL1/2 alone drive BRAFi/MEKi resistance in BRAF-mutant melanoma.[3] This information has now been added to the Results section (pg. 7). So, for the *NRAS*-mutant mouse melanoma lines, we checked whether, like the human lines, both ABL1/2 and DDR1 are required for resistance using combinations of ABL1/2- specific (GNF-5) and DDR1-specific inhibitors. Indeed, we found this to be the case (Supplementary Fig. 2f).

Nilotinib dramatically kills BMR and MR cells in the presence of D/T or trametinib (Supplementary Fig. 2b,c). Since we maintain our resistant cells in D/T or trametinib, we no longer routinely test the effects of nilotinib in the absence of D/T or trametinib as removing the drugs causes reversion of resistance or in some cases, causes cell death due to drug addiction. That being said, we did include one piece of data where D/T was removed two days prior to plating for the assay in order to assess the effects of nilotinib alone (Supplementary Fig. 2d). Nilotinib has no effect on its own, and only acts in concert with D/T or trametinib, and thus, is effective in reversing D/T (or trametinib) resistance.

11. In Suppl. Figure 3, cytokine expression in OSUMMER cells is not clearly represented. Some dots highlighted in squares do not correspond to the cytokines indicated. Panels a–d are confusing, and the array image overexposed may lead to misinterpretation of cytokine secretion patterns.

Unfortunately, the OSUMMER membranes had more background, which sometimes happens with these arrays, and thus, the dark exposure is dirty. In the revised manuscript, we removed the dark exposure and only show the light exposure which has less background. We also reorganized the figure to make it easier to follow and made sure that squares highlight the correct dots.

12. In Fig. 2 a, b and Suppl. Figure 3 e-h, downregulation at the mRNA level of some cytokines (Ccl5 in BRAF- and NRAS-mutant cells, Cxcl5 in NRAS-mutant cells) is not observed upon Nilotinib, despite their reduced secretion (Figure 1 and Suppl. Figure 3 b, c). Conversely, cytokine Cxcl5 downregulation observed by RNA-seq in BRAF-mutant cells (Suppl. Figure 3i) is not secreted in a consistent manner (Suppl. Figure 3a,b). Overall, the focus should be on secreted cytokines found in both human and mouse cells, and their relationship with immune cell infiltration, rather than on cytokine expression regulation, as the cytokine expression and secretion are not consistent in many cases.

We focused the manuscript on CXCR2 ligands (CXCL1, CXCL2, CXCL5, CXCL8) as well as on CCL2 and CCL5 because these cytokines are changed in human and mouse lines. Different cell lines appear to express different CXCR2 ligands. Since the ligands bind the same receptor on the surface of immune cells, the effect on the immune system would be predicted to be similar for all cell lines regardless of the CXCR2 ligand that is upregulated.

Nilotinib and ABL1/2 shRNAs have identical effects on the mRNA levels of CXCL1, CXCL8, IL-6 and CCL2. The only effects that are different are for CXCL2 (M14-BMR cells) and CCL5 (M14-BMR and SK-MEL-2MR). In these cases, nilotinib increases rather than decreases CXCL2/CCL5 mRNA expression. In new data shown in Fig. 2h-left, we show that nilotinib treatment increases ABL1 and ABL2 expression, and pCRKL (ABL1/2 substrate) blots show that these kinases are inactive due to the presence of nilotinib. Thus, we hypothesized that ABL1 and ABL2 induce CXCL2 and/or CCL5 expression in a kinase-independent manner. Indeed, kinase-dead forms (KR) of ABL1 and ABL2 were able to induce CXCL2 mRNA expression similar to the kinase active forms (see Fig. 2h-right), indicating that they drive CXCL2 expression in a kinase-independent manner. Interestingly, expression of kinase-active or kinase-dead forms of ABL1 and ABL2 was not sufficient to induce CCL5 mRNA indicating that ABL1/2 likely cooperate with another pathway to drive CCL5 mRNA expression.

RNA sequencing data has now been removed from the manuscript and is replaced with RT-qPCR data comparing cytokine expression in parental and resistant cells (Fig. 2f,g).

Moreover, it would be valuable to investigate how ABL1/2 mechanistically drive cytokine secretion. Is cytokine release dependent solely on MAPK pathway activation, or are additional signaling pathways involved?

Based on our previous data,[5] we believe that ABL1/2 likely promote expression of transcription factors that drive cytokine expression. However, determining which combination of transcription factors are involved and how ABL1/2 impacts their expression/activation could take several years to unravel as we would need to do a transcription factor screen, functional assays and ChIP assays to prove how ABL1/2 drive cytokine expression. Thus, this line of investigation, while important, we believe is beyond the scope of this initial paper. However, we do provide a discussion of some possibilities in the Discussion of the revised manuscript (pg. 17).

13. The authors should assess ABL activity in mouse tumors following the different treatments, at least in some models.

For our *in vivo* experiments, the mice were not all sacrificed at the same time since we wanted to have long-term follow-up information in order to determine how long ABL1/2 inhibition was effective. Thus, it wasn't possible to stain the tumors at the same timepoints to do a comparison. Moreover, for most of the mice, nilotinib is curative and there are no tumors present at the endpoint, and if we wait longer until the tumors eventually come back, we are assessing resistance to nilotinib rather than examining the ability of nilotinib to inhibit ABL1/2. It also is not possible to stain tumors with phospho-ABL antibody as the commercially available antibodies crossreact with phosphorylated receptor tyrosine kinases. In human tissue, we previously stained tumors with antibody to pCRKL (ABL1/2 substrate) as a readout of ABL1/2 activity;[6-8] however, while this antibody works consistently on human tissue, our Biospecimen facility was unable to get it to work consistently on mouse tissue. In our final attempt to address this question, we performed single-sample enrichment analysis on the melanoma cells from our single-cell RNAseq in order to estimate combined ABL1 and ABL2 "activities" based on the intersection of their downstream target genesets. After only 4 days treatment of mice with nilotinib, the combined activity of ABL1 and ABL2 was significantly downregulated in melanoma cells that were beginning to die (Fig. 6c and Supplementary Fig. 9e,f).

Moreover, all tumor growth data should be presented consistently, either as fold change in tumor volume or as absolute volume over time.

We changed the graphs so that all data are presented as tumor volume over time.

14. The authors demonstrate that the combination of D/T and nilotinib reduces MDSC infiltration leading to better tumor control and overcoming resistance to MAPKi. In that case it would be interesting to explore how exactly ABL1/2 inhibition influences the differentiation or recruitment of MDSCs. Are there any specific signaling pathways or transcription factors that mediate these changes?

While we agree that this will be an interesting study, and one we plan to do in the future, unraveling the mechanism for how ABL1/2 impacts MDSCs is likely to be a multi-year project, and thus, an investigation that we feel is beyond the scope of this initial manuscript, which is already long and complex.

15. In this study the authors focus on specific immune cell populations like MDSCs and $CD8^+$ T cells. But how might CXCL1, CCL2, and CCL5 expression affect other tumor-infiltrating immune populations (e.g., NK cells, Tregs etc.)? CCL5 is a cytokine that has been associated with the accumulation of Tregs.

The effect of overexpressing the cytokines on macrophage, NK cells, Tregs and B cells is now shown in Supplementary Fig. 8n-t. We found that CCL2 but not CCL5 overexpression increased splenic Tregs; however, no other significant differences in other immune populations were noted.

16. In Figure 5a-d, tumors derived from mice treated with D/T+nilotinib were too small to be analyzed via flow cytometric analysis, so blood and spleen were used as proxies for tumor immune infiltration. MDSCs are immune cells that can suppress the anti-tumor immune responses. But MDSCs in the blood or spleen may not behave the same as MDSCs infiltrating the tumor side. They may differ in their trafficking and in their survival. So, by measuring MDSCs in the blood/spleen may not accurately reflect local immunosuppression inside the tumor. This limitation should be clearly acknowledged.

We included a discussion of this limitation in the "Discussion" section on pg.18.

17. Paragraphs describing Figure 6 should be merged. Panels 6f–h and 6g–i should be combined to facilitate direct comparison between vehicle, D/T (or T), and D/T (or T) + nilotinib treatments.

We combined the text sections (pg. 12) and the indicated figures (now Fig. 6g,h).

18. The study demonstrates that nilotinib treatment in combination with MAPKi leads to increased infiltration of cytotoxic CD8$^+$ T cells and N1-like neutrophils, with the latter identified based on transcriptional profiles and expression of activation markers (Cebpb, Slpi). However, while CD8+ T cell depletion abolishes the anti-tumorigenic effect of nilotinib, the functional contribution of the N1 neutrophils remains unclear. Are these N1-like neutrophils truly cytotoxic in vivo, or do they reflect only a transcriptional signature without functional relevance? Further experimental evidence to validate their cytotoxic potential should be provided. Is the effect of N1-like neutrophils entirely dependent on CD8$^+$ T cells?

The focus of this manuscript is on how ABL1/2-driven secretion impacts MDSC and CD8$^+$ T cell infiltration during resistance. While we did observe increased neutrophils (likely N1) in small early tumors from nilotinib-treated mice, this finding is not a major focus of this paper. Therefore, we removed the mention of neutrophils from the section title and merged the paragraphs describing Fig. 6. Moreover, since only small tumors contain this N1 neutrophil population, determining whether they are functionally cytotoxic will not be trivial as we likely will not be able to isolate enough neutrophils to perform functional assays, and in all likelihood, the isolation procedure itself will kill these sensitive cells.

19. The study primarily assesses short-term (4-day) treatment effects. Would CD8$^+$ T cell and N1 neutrophil infiltration or function persist upon prolonged treatment?

In order to observe how the drugs impact initial immune infiltration, we performed short-term treatments. Long-term assays are shown for cytokine rescue studies. However, as noted above, for long-term assays, nilotinib treatment shrinks tumors until almost no tumor tissue is visible. It is impossible to perform flow cytometry on these small or non-existent tumors as there are not enough TILs to analyze. Moreover, as we described above, N1 neutrophil infiltration only occurs in smaller tumors with short drug treatment times. Once tumors get larger, the Ly6G$^+$ population becomes immunosuppressive rather than cytotoxic.

20. The legend corresponding to Figure 7g–i is missing and should be added.
We apologize for this mistake. The information has now been added to the figure legend.

References

1. Hong A, Moriceau G, Sun L, Lomeli S, Piva M, Damoiseaux R, *et al.* Exploiting Drug Addiction Mechanisms to Select against MAPKi-Resistant Melanoma. Cancer Discov **2018**;8:74-93

2. Lyon A, Tripathi R, Meeks C, He D, Wu Y, Liu J, *et al.* ABL1/2 and DDR1 drive MEKi resistance in NRAS-mutant melanomas by stabilizing RAF/MYC/ETS1 and promoting RAF homodimerization. Cancers (Basel) **2023**;15:954

3. Tripathi R, Liu Z, Jain A, Lyon A, Meeks C, Richards D, *et al.* Combating acquired resistance to MAPK inhibitors in melanoma by targeting ABL1/2-mediated reactivation of MEK/ERK/MYC signaling. Nature Communications **2020**;11:5463

4. Boivin G, Faget J, Ancey PB, Gkasti A, Mussard J, Engblom C, *et al.* Durable and controlled depletion of neutrophils in mice. Nat Commun **2020**;11:2762

5. Tripathi R, Fiore LS, Richards DL, Yang Y, Liu J, Wang C, *et al.* Abl and Arg mediate cysteine cathepsin secretion to facilitate melanoma invasion and metastasis. Sci Signal **2018**;11

6. Fiore LS, Ganguly S, Sledziona J, Cibull ML, Wang C, Richards DL, *et al.* c-Abl and Arg induce cathepsin-mediated lysosomal degradation of the NM23-H1 metastasis suppressor in invasive cancer. Oncogene **2014**;33:4508-20

7. Ganguly SS, Fiore LS, Sims JT, Friend JW, Srinivasan D, Thacker MA*, et al.* c-Abl and Arg are activated in human primary melanomas, promote melanoma cell invasion via distinct pathways, and drive metastatic progression. Oncogene **2012**;31:1804-16

8. He C, Plattner R, Rangnekar V, Zhou B, Liu C, Stewart RL*, et al.* Potential protein markers for breast cancer recurrence: a retrospective cohort study. Cancer Causes Control **2019**;30:41-51

RESPONSE TO REVIEWERS' COMMENTS

Responses to Reviewer #3

Reviewer #3 (Remarks to the Author):

R1. The activation of ABL1/2 in BRAF-mut cells appears modest and not fully convincing in Suppl. Fig. 2. The reproducibility of this observation is unclear and requires further validation. As the main effect of inhibiting ABL kinases is here described in murine models, a consistent signal of their activation is expected. Moreover, the ability of nilotinib to suppress this pathway should be confirmed in murine cell lines.

We replaced pCRKL blots for BRAF-mutant cell lines in Supplementary Fig. 2a with other replicates, and additional experiments are shown in Supplementary Fig. 2f and in the Source File. The effect of nilotinib on pCRKL phosphorylation has been included in Supplementary Fig. 2f.

R7, Figure 2. The green dots are not optimal for visualization and should be replaced with a more suitable color palette. Colors should also be harmonized across panels to improve clarity, contrast, and overall visual consistency.

Thank you for the suggestion. The editor has advised that she will help us revise the figures with a more suitable and more accessible palette, per journal requirements, should the manuscript be accepted for publication.

R9. The explanation provided remains insufficient. RT–qPCR analyses should be performed at earlier time points, in line with the cytokine secretion assays, to ensure temporal alignment and to support a more accurate interpretation of the data.

We performed nilotinib treatment for 12h, and found that the results were very similar to the results we observed following 24h treatment (see Appendix).

R14,18,19. The authors have not addressed the concerns raised, as no additional experiments or analyses have been provided. Overall, the responses would benefit from a more thorough and constructive approach.

R14. We show in the manuscript that ABL1/2 drive expression and secretion of chemokines CXCL1, CCL2 and CCL5, which are critical for ABL1/2 to promote MAPKi resistance and to induce MDSC tumor infiltration. Future experiments will unravel exactly how ABL1/2 impact chemokine secretion, assessing how they promote chemokine expression, stability and the secretion process itself. We also will determine how ABL1/2-driven chemokine secretion impacts MDSC function. These important questions, which require substantial additional experimentation, are likely to take several years to fully address. Therefore, as noted in our previous response, and supported by the editor's assessment, we believe that these studies fall beyond the scope of the current manuscript.

R18. We already showed the effect of long-term treatment of nilotinib on immune populations in the spleen (chemokine overexpression studies). As we indicated in the prior submission, for long-term assays, nilotinib treatment shrinks tumors until no tumor tissue is visible. It is impossible to perform flow cytometry on these small or non-existent tumors as there are few/no immune cells to analyze.

R19. Neutrophils are notoriously fragile, have a short life-span (6-8h), and frequently do not survive isolation procedures from blood and bone marrow, which is orders of magnitude easier than isolation from tumors, which requires incubation with proteases, and mechanical disruption. Indeed, single-cell RNAseq studies demonstrate that neutrophils are under-represented due to loss during isolation procedures.[1] Moreover, even if live functional neutrophils could be isolated, they often become activated during the separation process, which alters their phenotype and functional response for subsequent ex vivo assays.[1] We contacted a laboratory that specializes in isolating neutrophils from bone marrow, and asked if they could perform these assays for us. They said that it

is too difficult to isolate neutrophils from tumors, and also indicated that even if they were able to isolate functional neutrophils, the tumor size where we observe these neutrophils (100-150mm^3) is not large enough to be able to isolate enough neutrophils for functional assays as due to their fragility, it is difficult to get high yields. Thus, it is not possible for us to perform these experiments at this time.

However, we now include a new analysis on our single-cell RNAseq data to further support our findings. We reclustered the neutrophils into populations that are associated with "worst" and "best" prognoses.[2] We found that the majority of neutrophils isolated from the small D/T+nilotinib tumors have an HLA^+ phenotype, which is associated with T cell activation, and a "best" prognosis for cancer patients (Fig. 6f, right), and very few have a $VEGFR^+$ signature associated with the "worst" prognosis. These results further support the clustering data using signatures from Fetit et al. and indicate that the neutrophils likely have cytotoxic rather than immunosuppressive function.

We hope that these additional data are sufficient. If not, we would be happy to remove the neutrophil data from the manuscript if the reviewer (and/or the editor) do not believe that inclusion of these data add to the story.

Paragraph "ABL1/2 activities correlate with cytokine expression in patient samples" (page 6): The data support an association rather than a causal relationship. The text should be revised accordingly to avoid overinterpretation.

The text was revised.

Paragraphs "Nilotinib impacts infiltration of T cell subsets" and "Nilotinib treatment influences CD8+ T cell expression profiles" (page 12, 13): these sections could be merged into a single, more concise paragraph to improve flow and coherence.

The text was revised.

Paragraph "CXCL1 expression and ABL1/2 activities are elevated during BRAFi/MEKi resistance in patients" (page 14): This section lacks clarity and does not consistently refer to the relevant figures. Figure citations should be clearly indicated, and the text revised.

The text was revised to include more citations of the figures.

General comment: A thorough revision of the language is recommended throughout the manuscript, including the Discussion.

We double-checked the language in the manuscript. The editor indicated that any remaining issues will be resolved editorially once/if the manuscript is accepted.

References:

1. Salcher S, Sturm G, Horvath L, Untergasser G, Kuempers C, Fotakis G*, et al.* High-resolution single-cell atlas reveals diversity and plasticity of tissue-resident neutrophils in non-small cell lung cancer. Cancer Cell **2022**;40:1503-20 e8

2. Wu Y, Ma J, Yang X, Nan F, Zhang T, Ji S*, et al.* Neutrophil profiling illuminates anti-tumor antigen-presenting potency. Cell **2024**;187:1422-39 e24

RESPONSE TO REVIEWERS' COMMENTS

Responses to Reviewer #3.

We thank the reviewer for his/her thorough and insightful review of our manuscript. We are pleased that our manuscript is now acceptable for publication, in principle.
