# Supplementary material for: Targeting ABL kinases overcomes melanoma immune evasion following resistance to MAPK inhibition
Source: Nat Commun. 2026 Aug 21;17:9995. doi: 10.1038/s41467-026-76864-y (PMC13590603; doi:10.1038/s41467-026-76864-y)
Supplement: Supplementary file 3 [file 41467_2026_76864_MOESM3_ESM.pdf]

nature portfolio

Corresponding author(s): Plattner, Rina

Last updated by author(s): Jul 26, 2026

Reporting Summary

Nature Portfolio wishes to improve the reproducibility of the work that we publish. This form provides structure for consistency and transparency in reporting. For further information on Nature Portfolio policies, see our Editorial Policies and the Editorial Policy Checklist.

Statistics

For all statistical analyses, confirm that the following items are present in the figure legend, table legend, main text, or Methods section.

n/a	Confirmed	
☐	☒	The exact sample size (*n*) for each experimental group/condition, given as a discrete number and unit of measurement
☐	☒	A statement on whether measurements were taken from distinct samples or whether the same sample was measured repeatedly
☐	☒	The statistical test(s) used AND whether they are one- or two-sided<br>*Only common tests should be described solely by name; describe more complex techniques in the Methods section.*
☐	☒	A description of all covariates tested
☐	☒	A description of any assumptions or corrections, such as tests of normality and adjustment for multiple comparisons
☐	☒	A full description of the statistical parameters including central tendency (e.g. means) or other basic estimates (e.g. regression coefficient) AND variation (e.g. standard deviation) or associated estimates of uncertainty (e.g. confidence intervals)
☐	☒	For null hypothesis testing, the test statistic (e.g. *F*, *t*, *r*) with confidence intervals, effect sizes, degrees of freedom and *P* value noted<br>*Give P values as exact values whenever suitable.*
☒	☐	For Bayesian analysis, information on the choice of priors and Markov chain Monte Carlo settings
☒	☐	For hierarchical and complex designs, identification of the appropriate level for tests and full reporting of outcomes
☒	☐	Estimates of effect sizes (e.g. Cohen's *d*, Pearson's *r*), indicating how they were calculated

Our web collection on statistics for biologists contains articles on many of the points above.

Software and code

Policy information about availability of computer code

Data collection

TCGA-SKCM gene expression data was obtained from GDC portal in Nov. 2019, and the corresponding clinical data was downloaded from cBioPortal. Data from MEKi-treated NRAS-mutant melanomas (Nagler et al.) were obtained from Adi Nagel and Yardena Samuels in Dec. 2023. The data have since been deposited (GSE198776). Data from patients with BRAF-mutant, MAPKi-resistant tumors from GSE65185 data were downloaded (GEO) in 2019. Data from Vasilevska et al. provided by authors, Drs. Cheng and Levesque have been deposited in GEO (GSE229908). Single-cell RNA sequencing generated in this manuscript have been included as Supplementary Material, and the raw files have been deposited to GEO (GSE325262).

Data analysis

Statistical Analyses. In vitro and in vivo studies. One-Way Analysis of Variance (ANOVA; >2 comparisons), two sample (comparisons between two groups) or one sample t-tests (comparisons to normalized controls) were used to analyze in vitro experiments (using GraphPad Prism or Vassar Website). The Holm's method for multiple comparisons adjustment was used for one- and two-sample tests and Tukey's multiple comparisons test used for one-way ANOVAs. In vivo studies were analyzed with the linear mixed model (SAS, v9.4) to compare log-transformed tumor volumes between treatment groups. For survival analyses, Kaplan-Meier and Logrank tests (R, v4.3.1) were used to compare time to tumor doubling. In all cases, two-tailed p values were reported (p<0.05 was considered statistically significant).

Sample-wise Geneset Enrichment Analysis. The following datasets were utilized for the analyses. 1) TCGA melanoma database (n=471); 2) samples from patients with NRAS-mutant melanomas treated with MEKi (n=23; GSE198776);28 and 3) samples from patients with BRAF-mutant melanomas treated with MAPKi (GSE65185)29 using only samples from patients demonstrating resistance to the MAPKi regimen, and removing samples from patients who were not treated with MAPKi or were sensitive to the regimen. Moreover, replicates from the same patients were removed by taking an average, leaving a final sample size of n=24. Sample-wise gene set enrichment analyses methods, such as those implemented in Gene Set Variation Analysis (GSVA v1.50.0) package, transform gene expression data into pathway-centric scores at the single-sample level, where an enrichment score is computed to quantify the pathway activity for each pathway in each sample. Using GSVA and the Ingenuity Pathway Analysis (IPA) database (genes and their associated downstream targets integrated from published data;

Supplementary Dataset 1),26 we calculated enrichment scores to quantify kinase activities in individual melanomas based on RNAseq data, where a high positive enrichment score indicates that the target geneset (kinase activity) is highly upregulated within a particular sample. The calculated enrichment scores were then used for Spearman's correlation analyses to assess the correlations between ABL kinase activities and cytokine mRNA expression. Some of the targets in the IPA genelists utilized for performing the single-sample enrichment analysis (see Supplementary Dataset 1) were validated by RT-qPCR using M14-BMR cells engineered to express either non-targeting shRNA (shNT) or shRNA targeting ABL1 and ABL2 (see Supplementary Table 1).

Single-Cell RNA sequencing (scRNAseq). Single-cell gene expression libraries were mapped to the mm10 mouse reference (10X Genomics pre-built reference, mm10-2020-A) using Cell Ranger (v6.0.0, 10X Genomics). For each scRNAseq library, the Cell Ranger filtered matrix was subjected to QC filtering. Based on the distribution of library complexity, low quality cells with ≤200 expressed genes, ≤500 unique molecular barcodes or ≥ 20% mitochondrial transcripts were removed. Doublets were detected and filtered using scDblFinder (v1.14.0)66 with default parameters. Cells that passed the QC from D/T and D/T+nilotinib groups were integrated using Seurat (4.4.0) SCT-based integration workflow.67 Each dataset was individually normalized and scaled using the SCTransform function.68 The top 2,000 most variable features across datasets were subsequently used to identify integration anchors and merge the datasets into an integrated object. The shared nearest neighbor (SNN) graph was constructed based on the first 50 principal components. Cell clusters were then identified by applying unsupervised clustering using FindClusters function with Louvain algorithm (resolution 0.5) resulting in 31 clusters. The Uniform Manifold Approximation and Projection (UMAP) on the 50 principal components was generated for a two-dimensional embedding of the integrated scRNA-seq datasets. Cluster marker genes and differential gene expression analyses between the two experimental groups was performed using a Wilcoxon rank-sum test. The cell types were annotated according to expression of canonical markers in Supplementary Fig. 11c. Malignant melanoma cells were identified based on the widespread chromosomal amplification and deletion patterns inferred using InferCNV (1.20.0) (Supplementary Fig. 11d). Dying melanoma cells were defined as melanoma cells with copy number variations, and an increased apoptosis-like state as indicated by elevated activity of apoptosis-related pathways (Supplementary Fig. 11e). Gene signatures from healthy, tumor-associated, or metastasis-associated neutrophils were obtained from Fetit et al.41 Signatures for HLA-DR+CD74+ and VEGFA+SPP1+ neutrophils42 were derived from the human pan-cancer neutrophil atlas, defined as the top 20 marker genes of each cluster. Human gene symbols were converted to mouse orthologs using babelgene (https://github.com/igordot/babelgene). To quantify the activity of specific gene signatures, we calculated the module scores at the single cell level using the AddModuleScore function. Geneset enrichment analysis was performed using the fgsea (1.20.0) R package69 based on the ranked gene list from the differential analysis. The two-sample binomial test was used to compare the distribution of cell clusters between the 2 groups. T cell subtype annotation. To assess differences in T cell states between D/T and D/T+nilotinib treatments, we projected our scRNA-seq data onto a mouse tumor-infiltrating lymphocyte (TIL) reference atlas using the ProjecTILs R package (v3.0).44 The reference atlas defines nine distinct T cell states based on murine tumor scRNA-seq data. A normalized expression matrix was used as input, and non-T cells were excluded prior to projection. Query cells were aligned to the reference UMAP embedding using the make.projection function with default parameters, and T cell states were assigned via cellstate.predict using a nearest-neighbor classifier. Projected cells were visualized on the reference UMAP using plot.projection. T cell annotations were further validated by examining the expression of markers defined by the projectTILs reference atlas.

Analysis of scRNAseq from patient samples from Vasilevska et al (GSE229908).52 For each patient, pairwise differential expression analysis was performed for each post-treatment biopsy and baseline biopsy on the tumor cells using the findMarkers function from scran.70 Gene Set Variation Analysis (GSVA, v1.50.0) enrichment scores were computed based on the normalized scRNAseq expressions of the downstream target gene sets of ABL1, ABL2, and DDR1 within each patient, treating each malignant cell as a sample. Box plots of the computed GSVA enrichment scores were generated to compare the activities of ABL1, ABL2, and DDR1 among the timepoints. Overall differences in GSVA enrichment scores for ABL1, ABL2, and DDR1 across timepoints were assessed using non-parametric Kruskal–Wallis tests. When the overall test was significant, pairwise comparisons between timepoints were performed using non-parametric Wilcoxon rank-sum tests. Resulting p-values were adjusted for multiple comparisons using Holm's method. All details can be found in Supplementary Table 1.

For manuscripts utilizing custom algorithms or software that are central to the research but not yet described in published literature, software must be made available to editors and reviewers. We strongly encourage code deposition in a community repository (e.g. GitHub). See the Nature Portfolio guidelines for submitting code & software for further information.

Data

Policy information about availability of data

All manuscripts must include a data availability statement. This statement should provide the following information, where applicable:

- Accession codes, unique identifiers, or web links for publicly available datasets
- A description of any restrictions on data availability
- For clinical datasets or third party data, please ensure that the statement adheres to our policy

Source data are provided with this paper. Single-cell RNA sequencing generated in this manuscript have been included as Supplementary Material, and the raw files have been deposited to GEO (GSE325262). TCGA-SKCM gene expression data was obtained from GDC portal in Nov. 2019, and the corresponding clinical data was downloaded from cBioPortal. Data from MEKi-treated NRAS-mutant melanomas (Nagler et al.) were obtained from Adi Nagel and Yardena Samuels in Dec. 2023. The data have since been deposited (GSE198776). Data from patients with BRAF-mutant, MAPKi-resistant tumors from GSE65185 data were downloaded (GEO) in 2019. Data from Vasilevska et al., provided by authors, Drs. Cheng and Levesque, have been deposited in GEO (GSE229908).

Research involving human participants, their data, or biological material

Policy information about studies with human participants or human data. See also policy information about sex, gender (identity/presentation), and sexual orientation and race, ethnicity and racism.

Reporting on sex and gender	We utilized previously published clinical data.
Reporting on race, ethnicity, or other socially relevant groupings	N/A
Population characteristics	N/A
Recruitment	N/A

Ethics oversight	N/A

Note that full information on the approval of the study protocol must also be provided in the manuscript.

Field-specific reporting

Please select the one below that is the best fit for your research. If you are not sure, read the appropriate sections before making your selection.

- [x] Life sciences
- [ ] Behavioural & social sciences
- [ ] Ecological, evolutionary & environmental sciences

For a reference copy of the document with all sections, see nature.com/documents/nr-reporting-summary-flat.pdf

Life sciences study design

All studies must disclose on these points even when the disclosure is negative.

Sample size	In vitro assays were performed using >3 biological replicates. Individual replicates are shown as dots within bar graphs or box-and-whisker plots. For animal experiments, power analyses were performed prior to experiments. Ten mice/group (5 male/5 female) provides >95% power to detect differences in mean tumor volume at day 40 across all groups (ANOVA, 1% alpha; guided by Tripathi et al. 2020). This sample also provides >95% power to compare the proportion of relapsed mice in D/T vs. D/T+nilotinib groups (chi-square test, 5% alpha; based on Tripathi et al. 2020).
Data exclusions	Data >2 standard deviations from the mean were excluded. FACS analysis was limited to tumors of similar size. Samples were not utilized if low immune cell numbers were observed (<100 cells in a gated population). Animals who died of experiment-independent causes (e.g. gavage injury) at early time points were removed from the study.
Replication	In vitro experiments involve use of at least 3 biological replicates in addition to technical replicates except for colony assays and western blots for some cell lines shown in Supplementary Fig. 2b-f, which are representative of n=2 biological replicates. Replicates are shown in the Source File. Due to expense, antibody arrays are from n=1, but were repeated with multiple cell lines. Moreover, follow-up experiments (RT-qPCR) were performed to validate array results.
Randomization	Mice were randomized to treatment groups prior to treatment by personnel that were not involved in treatment of mice. Excel was used for randomization. Mice were ranked based on tumor size and every other mouse was assigned to the same group. This randomization process assured that the mean starting tumor sizes were equivalent/similar among the groups.
Blinding	FACS analysis was performed by Shared Resource personnel who were blinded to the experimental conditions. For all animal experiments, laboratory personnel were blind to the randomization process, which was performed by the PI.

Reporting for specific materials, systems and methods

We require information from authors about some types of materials, experimental systems and methods used in many studies. Here, indicate whether each material, system or method listed is relevant to your study. If you are not sure if a list item applies to your research, read the appropriate section before selecting a response.

Materials & experimental systems

n/a	Involved in the study	
[ ]	[x]	Antibodies
[ ]	[x]	Eukaryotic cell lines
[x]	[ ]	Palaeontology and archaeology
[ ]	[x]	Animals and other organisms
[ ]	[x]	Clinical data
[x]	[ ]	Dual use research of concern
[x]	[ ]	Plants

Methods

n/a	Involved in the study	
[x]	[ ]	ChIP-seq
[ ]	[x]	Flow cytometry
[x]	[ ]	MRI-based neuroimaging

Antibodies

Antibodies used	Please see Supplementary Table 1 for a list of antibodies, commercial vendors , catalog numbers and dilutions utilized.
Validation	All antibodies utilized are commercially available and were obtained from Cell Signaling, Sigma, Santa Cruz Biotechnology, etc. Antibody validation policies are present on the company websites. We also independently validate antibodies by utilizing them on lysates with knockdown of the protein of interest. In addition, RRID numbers for all antibodies can be found in Supplementary Table 1.

Eukaryotic cell lines

Policy information about cell lines and Sex and Gender in Research

Cell line source(s)	Please see Supplementary Table 1 for a list of cell lines and their sources. Cell lines were obtained from commercial sources or directly from the establishing laboratory. Mouse YUMM and OSUMMER lines as well as 293T cells were purchased from the ATCC. MaNras1-1007 and MaNRAS2-1014 were from Drs. Larue and Aplin. STR analysis was used to authenticate cell lines. Human MAPKi-resistant cell lines were previously described in our manuscripts (Tripathi et al; Lyon et al.; M14-BMR, SK-MEL-2MR). In this manuscript, mouse resistant lines were established by culturing in increasing doses of dabrafenib/ trametinib (BRAFi/MEKi; BRAF-mutant) or trametinib (NRAS-mutant).
Authentication	All cell lines were either purchased from ATCC or authenticated by STR analysis.
Mycoplasma contamination	Cell lines were tested for mycoplasma during the course of the experiments.
Commonly misidentified lines (See ICLAC register)	No misidentified cell lines have been utilized.

Animals and other research organisms

Policy information about studies involving animals; ARRIVE guidelines recommended for reporting animal research, and Sex and Gender in Research

Laboratory animals	Species and strains of mice are reported in Supplementary Table 1. All mice were purchased from JAX (C57BL/6J-stock #000664; BRAF/PTEN mice-stock #13590). All animals were housed in a pathogen-free/germ free/GF, with experimental and control animals co-housed. All mice utilized were 6-8 weeks of age.
Wild animals	N/A
Reporting on sex	The sex of the mice must be congruent with the cell line for syngeneic models in order to prevent immune rejection. Female mice were utilized by YUMM3.3 and OSUMMER.12 models, while male mice were used for YUMM5.2 and YUMM1.7 models. BRAF/PTEN mouse experiments utilized male (9) and female (15) mice.
Field-collected samples	None utilized
Ethics oversight	The University of Kentucky Institutional Animal Care and Use (IACUC) committee approved these studies: protocol-2020-3426

Note that full information on the approval of the study protocol must also be provided in the manuscript.

Clinical data

Policy information about clinical studies

All manuscripts should comply with the ICMJE guidelines for publication of clinical research and a completed CONSORT checklist must be included with all submissions.

Clinical trial registration	N/A
Study protocol	N/A
Data collection	N/A
Outcomes	N/A

Plants

Seed stocks	N/A
Novel plant genotypes	N/A
Authentication	N/A

Flow Cytometry

Plots

Confirm that:

- [x] The axis labels state the marker and fluorochrome used (e.g. CD4-FITC).
- [x] The axis scales are clearly visible. Include numbers along axes only for bottom left plot of group (a 'group' is an analysis of identical markers).
- [x] All plots are contour plots with outliers or pseudocolor plots.
- [x] A numerical value for number of cells or percentage (with statistics) is provided.

Methodology

Sample preparation	Melanomas from C57BL/6 mice harboring BRAF-mutant YUMM5.2 or NRAS-mutant OSUMMER.12 tumors, treated with D/T or Tra versus D/T+nilotinib or Tra+Nilo for 4 days, were harvested from euthanized mice, tumors fragmented and dissociated in digestion cocktail (Type IV collagenase-33.3mg/ml, haluronidase-3.3mg/ml, Type I DNase-0.33mg/ml) for 1h at 37oC, and passed through 70uM nylon mesh. Single cell suspensions were washed with PBS, cells counted (TC20, BioRad), incubated in FC blocking antibody and fixable live/dead stain (see Table S1) for 15' at 4oC.
Instrument	BD Symphony or Cytek Aurora
Software	FlowJo v10, v11
Cell population abundance	Single cell suspensions were washed with PBS, cells counted (TC20, BioRad, Hercules, CA), incubated in FC blocking antibody and fixable live/dead stain (see Table S1) for 15' at 4oC. Antibody cocktails were added (see Table S1) and incubated for 45'. Following washing, cells were fixed for 20' at 4oC, resuspended in FACS buffer, and analyzed, gating 0.1-1x10E6 live events or all events.
Gating strategy	Gating strategies are shown in Supplementary Figs. 6,7i, 8h, and 10a.

- [x] Tick this box to confirm that a figure exemplifying the gating strategy is provided in the Supplementary Information.
