# Supplementary material for: Targeting ABL kinases overcomes melanoma immune evasion following resistance to MAPK inhibition
Source: Nat Commun. 2026 Aug 21;17:9995. doi: 10.1038/s41467-026-76864-y (PMC13590603; doi:10.1038/s41467-026-76864-y)
Supplement: Supplementary file 4 [file 41467_2026_76864_MOESM4_ESM.pdf]

Description of Additional Supplementary Files

Supplementary Data 1
Description: IPA genesets used for single-sample enrichment analysis.

Supplementary Data 2
Description: Marker genes for cell types in single-cell RNA sequencing data. Data from this table was used to generate Supplementary Fig. 9c. Tab 1. B cells. Tab 2. CD4+ T cells. Tab 3. CD4/CD8-negative T cells. Tab 4. CD8+ T cells. Tab 5. classical DCs (cDCs). Tab 6. myeloid dendritic cells (mDCs). Tab 7. plasmacytoid dendritic cells (pDCs). Tab 8. monocytes. Tab 9. macrophages. Tab 10. neutrophils. Tab 11. neutrophils sorted by fold change. Tab 12. NK cells. Tab 13. Fibroblasts. Tab 14. endothelial cells. Tab 15. Mast cells. Tab 16. Melanoma cells. Tab 17. Dying melanoma cells. Tab 18. smooth muscle cells.

Supplementary Data 3
Description: Differential expression analysis in single-cell RNA sequencing data comparing D/T+nilotinib versus D/T-treated tumors. Differentially expressed genes comparing mice treated with D/T+nilotinib to those treated with D/T alone. Tab 1. All immune cells p-values. Tab 2. Differentially expressed genes in CD4+ T cells. Positive values indicate that the genes are upregulated in nilotinib-treated animals. Tab 3. Differentially expressed genes in CD8+ T cells. Tab 4. Differentially expressed genes in neutrophils. Tab 5. Differentially expressed genes in melanoma cells. Tab 6. Differentially expressed genes in the dying melanoma population.

Supplementary Data 4
Description: Marker genes for T cell subtypes in single-cell RNA sequencing data. Data from this table was used to generate Fig. 7b. Tab 1. CD8 Effector Memory. Tab 2. CD8+ Tpex. Tab 3. CD8+ Tex. Tab 4. CD8+ Early Active. Tab 5. CD8+ Naive-like Tab 6. CD4+ Tregs. Tab 7. CD4+ Th1. Tab 8. CD4+ Naive-like. Tab 9. CD4+ Tfh.

Supplementary Data 5
Description: Differential expression analysis of CD4+ and CD8+ T cell populations by single-cell RNA sequencing. Tab 1. CD8+ effector memory cells. Tab 2. CD8+ Early Active cells. Tab 3. CD8+ Tpex cells. Tab 4. CD8+ Naive-like cells. Tab 5. CD4+ Tregs. Tab 6. CD4+ Th1 cells. Tab 7. CD4+ Naive-like cells. Tab 8. CD8+ T cell subset p-values. Tab 9. CD4+ T cell subset p-values.

Supplementary Data 6
Description: Geneset enrichment analysis (GSEA) of CD4+ and CD8+ T cell populations by single-cell RNA sequencing. Tab 1. GSEA KEGG Pathway analysis of CD8+ T cells; Tab 2. GSEA HALLMARK Pathway (CD8+ T cells); Tab 3. GSEA REACTOME Pathway (CD8+ T cells).

Supplementary Data 7
Description: Differential expression analysis of data from Cohort 1 (Vasilevska et al. 2024). Tab 1=comparison of Patient 3 (baseline) to Patients 1,2 (baseline). Tabs 2-5. Comparison of post-treatment (PT) samples to baseline. Tab 2.Patient 1; Tab 3=Patient 2; Tab 4. Patient 3; Tab 5. Patient 4.
